# Altered cognitive performance is associated with auditory cortex functional connectivity in patients with unilateral sensorineural hearing loss

**DOI:** 10.3389/fnins.2026.1902588

**Published:** 2026-08-18

**Authors:** Junming Chen, Bochen Wang, Youjun Yu, Xiaowei Zhou, Yuanxin Zhao, Xiaoling Wen, Rongbin Qin, Yaowei Guan

**Affiliations:** 1 Department of Otorhinolaryngology—Head and Neck Surgery, Ear Medicine Center, The First People’s Hospital of Foshan, Foshan, China; 2 College of Electronic and Information Engineering, Guangdong Ocean University, Zhanjiang, China

**Keywords:** cognitive function, functional connectivity, mismatch negativity, partial granger causality analysis, unilateral sensorineural hearing loss

## Abstract

Sensorineural hearing loss (SNHL) has been suggested to be closely associated with cognitive decline, yet the underlying neural mechanisms remain largely unclear. This study sought to explore the potential association between cognitive function and auditory cortex functional connectivity in patients with unilateral sensorineural hearing loss (USNHL) and investigate the possible neural mechanisms underlying subtle cognitive decline. Sixty patients with moderate-to-severe USNHL (disease duration ≥3 years; 30 with right-sided USNHL and 30 with left-sided USNHL) and 30 age-matched normal-hearing controls were enrolled; all participants underwent Montreal Cognitive Assessment (MoCA) and oddball paradigm electroencephalography, mismatch negativity (MMN) amplitude and latency were compared, and standardized low-resolution electromagnetic tomography (sLORETA) combined with partial Granger causality (PGC) was applied to analyze directional functional connectivity among the superior temporal gyrus (STG), inferior frontal gyrus (IFG), parahippocampal gyrus (PHG), and posterior cingulate cortex (PCC), with correlation and serial mediation analyses conducted. Relative to controls, USNHL patients exhibited lower MoCA scores, reduced MMN amplitude, prolonged latency, decreased left STG → IFG/STG → PHG/PHG → PCC connectivity and enhanced left IFG → STG connectivity; left IFG → STG connectivity was negatively correlated with MoCA attention scores, whereas left PHG → PCC connectivity was positively correlated with MoCA memory scores, with more prominent abnormalities in patients with severe USNHL. Serial mediation analysis suggested that the link between hearing loss severity and cognitive alterations might be mediated by two domain-specific serial pathways, both initiated by prolonged MMN latency: an attention pathway (MMN latency → enhanced left IFG → STG connectivity) and a memory pathway (MMN latency → reduced left PHG → PCC connectivity), and USNHL patients displayed left hemispheric lateralization of PGC alterations. These findings imply that enhanced left IFG → STG and weakened left PHG → PCC connectivity may be related to attention and memory alterations respectively, which may represent neural correlates of cognitive decline in middle-aged adults with USNHL.

## Introduction

Sensorineural hearing loss (SNHL) has been identified as a risk factor for Alzheimer’s disease (AD), and subclinical hearing loss is also significantly associated with reduced cognitive function. Notably, AD progressively impairs cognitive function, physical function, and mental health, ultimately leading to loss of independent living ability and diminished quality of life (Park et al., 2022). Therefore, clarifying the mechanism by which SNHL is associated with cognitive alteration is highly important for the prevention and treatment of AD. Notably, unilateral sensorineural hearing loss (USNHL) is a common clinical subtype of SNHL, accounting for approximately 7.9% of all SNHL cases (Asp and Reinfeldt, 2020). Despite its high prevalence, most previous studies have focused on bilateral SNHL, leaving the neural mechanisms linking USNHL to cognitive decline unclear. USNHL provides a unique clinical model to dissect the specific associations between auditory deprivation and brain function: unlike bilateral SNHL, which causes global auditory input loss, USNHL leads to asymmetric auditory input, allowing us to isolate the effects of unilateral sensory deprivation on directional neural interactions within the auditory-cognitive network—without the confounding effects of bilateral auditory loss. This makes USNHL an ideal study population to explore the link between auditory network dysfunction and cognitive decline, as it enables the identification of hemisphere-specific neural changes that may be masked in bilateral SNHL. Thus, exploring the neuroelectrophysiological correlates of USNHL-induced cognitive alteration and clarifying the abnormal pattern of directional neural interactions within the auditory-cognitive network is of great significance for revealing the association between hearing loss and cognitive decline.

Using rs-EEG, Cai et al. (2019) found that USNHL patients showed significantly reduced lagged coherence between the central cingulate cortex and prefrontal regions, indicating disrupted communication within the cingulo-prefrontal executive control network. These findings were particularly notable because they demonstrated that connectivity abnormalities within the auditory-attention network and cingulo-prefrontal executive network are present even in the acute phase of unilateral auditory deprivation, prior to structural brain changes. Using rs-fMRI, Qiao et al. (2022) investigated intrinsic brain activity alterations in USNHL patients with duration exceeding 2 years. They found that patients exhibited prominent aberrations within core nodes of the default mode network (DMN). No significant intrinsic activity changes were detected in the STG, indicating that long-term monaural deprivation predominantly remodels higher-order cognitive association cortices such as PCC rather than primary auditory regions. Collectively, these findings converge to demonstrate that unilateral deafness induces a hierarchical, hemisphere-specific reorganization of auditory-cognitive networks that begins at early sensory processing stages and extends to affect large-scale network dynamics supporting attention, executive function, and memory. However, most studies focus on resting-state and undirected functional connectivity and cannot fully characterize the dynamic, directionally specific neural interactions underlying cognitive dysfunction.

Event-related potential (ERP) serves as an objective indicator for assessing central auditory processing and cognitive function, providing an ideal research tool for analyzing this neural mechanism (Doan et al., 2021). MMN is an endogenous ERP elicited by the presentation of a deviant auditory stimulus following repeated exposure to a standard stimulus, and it serves as a well-recognized objective neurophysiological marker for investigating cognitive function in both clinical and experimental settings (Chen et al., 2022). In the present study, MMN was identified as a negative component in the range of 150–300 ms, which was calculated by subtracting the deviant waveform from the standard waveform (Liang et al., 2014; Chen et al., 2022). Changes in MMN amplitude can reflect changes in central auditory processing functions such as auditory discrimination and sensory memory, and a reduced MMN amplitude indicates a decreased cognitive function in patients with SNHL (Pei et al., 2020). Wang et al. (2022) reported that when hearing is impaired, the quality of signals received by the auditory cortex decreases, leading to prolonged MMN latency and changes in MMN source localization. These changes indicate delayed information processing in the auditory cortex and subsequent central functional reorganization, which is associated with modified cognitive regulation. These results suggest that patients with SNHL exhibit disorders in central functional activities related to cognition during the MMN period. To explore which central activities are impaired during the MMN period and affect cognitive function, further studies were conducted. Converging evidence from source localization and effective connectivity studies in healthy and clinical populations indicates that MMN-related pre-attentive auditory-cognitive processing is supported by two functionally dissociated but interconnected hierarchical pathways.

The first is the auditory-attention pathway (bidirectional STG–IFG connections supporting deviance detection and attentional regulation). Recent studies have shown that MMN originates from the superior temporal gyrus (STG) and the inferior frontal gyrus (IFG) (Ozbayrak-Karapinar et al., 2025), with both temporal and frontal lobes participating in cognitive processing (Kim et al., 2020). Using event-related EEG and standardized low-resolution electromagnetic tomography (sLORETA) in healthy individuals, Kitaura et al. (2017) observed increased information flow between STG and IFG relative to the resting state in the left hemisphere. They presumed left STG-to-IFG connectivity as bottom-up transmission of auditory signals for cognitive processing, whereas the reverse IFG-to-STG pathway mediates top-down regulation, including result verification and information prioritization. By applying the auditory oddball paradigm and dynamic causal modeling (DCM), Larsen et al. (2024) further reported attenuated STG-driven influence on the IFG in the left hemisphere, reflecting reduced perceptual sensitivity to deviant stimuli and potentially long-term impairments in attention, memory, and other cognitive functions.

The second is the auditory-memory pathway (serial STG → PHG → PCC connections supporting memory encoding and integration). In addition, the STG relays auditory information to the parahippocampal gyrus (PHG) of the limbic system to mediate memory-related cognitive processing. Using event-related EEG, Liu et al. (2025) observed sequential activation of the STG and PHG following stimulation in healthy subjects in bilateral hemisphere. They proposed that the STG primarily analyses and categorizes early auditory signals, extracts basic acoustic features, and supplies raw perceptual information for subsequent high-level processing. Following early perceptual coding by the STG, the PHG is activated to retrieve sound-related memories and match perceptual inputs with memory stores, enabling the integration of auditory memory and semantic comprehension. Previous studies have demonstrated weakened STG–PHG functional connectivity in patients with SNHL. Li et al. (2025) applied resting-state EEG and sLORETA and identified reduced bilateral STG-PHG connectivity in children with congenital SNHL. They reported that diminished functional coupling between the PHG and auditory cortex disrupts auditory information-memory associations, impairing the binding of acoustic cues (e.g., language, environmental sounds) to corresponding memory representations and thus hindering learning and knowledge acquisition. Changes in PHG function alter the transmission of information to the posterior cingulate cortex (PCC), affecting memory-related cognitive activities. Steven Waterstone et al. (2020) analyzed resting-state EEG and sLORETA data from patients with subacute to chronic stroke and reported reduced bilateral PHG–PCC functional connectivity. The PCC, a core node of the default mode network (DMN), mediates memory retrieval and integration by combining PHG-encoded memory information with the brain’s preexisting knowledge network to support episodic memory recall and application. Decreased PHG-PCC functional connectivity indicates altered episodic memory function in these patients.

In recent years, functional magnetic resonance imaging (fMRI) has markedly advanced understanding of brain network plasticity and reorganization in SNHL, especially within the default mode network (DMN), frontoparietal control network, and auditory-related networks. However, most fMRI studies focus on undirected functional connectivity with limited temporal resolution and cannot fully characterize the dynamic, directionally specific neural interactions underlying cognitive dysfunction. EEG offers complementary advantages owing to its high temporal resolution for assessing directional effective connectivity, yet such studies remain limited. Collectively, these data suggest that SNHL may disrupt cognitive function by altering functional connectivity among the temporal lobe, frontal lobe, and limbic system. Given the high prevalence of USNHL and its unique value for investigating asymmetric auditory deprivation–induced neural changes without complete deafness, we formulated three hypotheses. First, unilateral auditory deprivation in USNHL may induce hemisphere-specific alterations in directional effective connectivity within both the auditory-attention and auditory-memory pathways. Second, the directional connectivity strength of the STG and IFG auditory-attention pathway may be correlated with individual attentional performance, while the connectivity strength of the STG, PHG and PCC auditory-memory pathway may be correlated with episodic memory performance. Third, altered MMN responses may mediate the associations between unilateral auditory deprivation and distinct cognitive decline through two separate auditory-cognitive neural pathways.

## Materials and methods

The study protocol was approved by the Institutional Review Board of the First People’s Hospital of Foshan, Foshan, China (see Supplementary materials for the ethical approval document), and written informed consent was obtained from all participants. The studies were conducted in accordance with the local legislation and institutional requirements. The participants provided their written informed consent to participate in this study.

### Grouping

Study group: Sixty patients with moderate-to-severe (Chadha et al., 2021) USNHL (30 with right SNHL and 30 with left SNHL) were selected. The inclusion criteria were as follows: aged 18–60 years; USNHL with a duration of at least 3 years; no history of hearing aid use; right-handedness; otoscopic examination showing a normal external auditory canal and tympanic membrane; pure-tone audiometry (PTA) showing an average hearing threshold of 35–80 dBHL in the range of 500–4,000 Hz; an acoustic immittance test revealing normal middle ear function; and no sustained tinnitus, vertigo, neurological or psychiatric diseases, or other systemic diseases. The exclusion criteria for patients were as follows: (1) clear local otological causes; (2) acoustic neuroma or another condition requiring surgical or other treatments; (3) severe internal disease, such as hypertension, diabetes, hyperlipidemia, menopausal syndrome, etc., not controlled within the normal range and requiring internal medical treatment; and (4) severe psychological disorder requiring professional psychological treatment. There was no significant difference in duration of hearing loss between the right and left SNHL patients (right SNHL: 4.7 ± 0.9 years; left SNHL: 4.3 ± 1.1; *t* = 1.5, *p* = 0.13). We selected adults aged 18–60 years as the study population to exclude the confounding effects of age-related neurodegeneration (e.g., brain atrophy, spontaneous decline of cognitive function) on brain functional connectivity and cognitive assessment, which can help us accurately identify the neural changes solely induced by USNHL. Although age-related hearing loss (ARHL) is more prevalent in older adults (≥65 years), the core neural mechanism of auditory deprivation-induced functional connectivity abnormalities in the auditory-cognitive network is consistent across different age groups (Gao et al., 2023). The ≥3-year disease duration threshold was chosen based on evidence that auditory deprivation-induced central plastic remodeling reaches a relatively stable phase after approximately 2–3 years of sensory deprivation (Firszt et al., 2018), ensuring that the observed connectivity alterations reflect established neuroadaptive changes. Patients with a history of hearing aid use were excluded to avoid confounding effects of intervention-induced plasticity, as hearing aid rehabilitation itself can reshape auditory-cognitive network connectivity and potentially mask or reverse deprivation-related alterations (Gao et al., 2023).

Control group: Thirty healthy volunteers aged 18–60 years were recruited. The inclusion criteria were as follows: age 18–60 years; right-handedness; otoscopic examination showing a normal external auditory canal and tympanic membrane; PTA showing an average hearing threshold < 20 dBHL in the range of 500–4,000 Hz; an acoustic immittance test showing normal middle ear function; and no history of noise exposure, tinnitus, vertigo, neurological or psychiatric diseases, hypertension, diabetes, cardiovascular diseases, or other systemic diseases.

Hearing asymmetry was quantified using the absolute PTA difference between the affected ear and the contralateral ear (Affected ear PTA − Contralateral ear PTA, dBHL). Participants were stratified into two subgroups by hearing loss severity: moderate USNHL (35–64 dBHL) and severe USNHL (65–79 dBHL). (Supplementary Tables S19–S24). This dichotomous split was adopted to ensure adequate statistical power per subgroup, and its robustness was further verified against both WHO 2021 and WHO 1997 grading standards, with all core findings remaining significant (effect size changes <5%; Supplementary Table S36).

### Montreal cognitive assessment (MoCA)

The Montreal Cognitive Assessment (MoCA), Chinese (Beijing) Version 7.1—the Mandarin Chinese adaptation of the original scale developed by Nasreddine et al. (2005)—was used to assess cognitive function. It evaluates seven core cognitive domains, including visuospatial/executive function (max 5 points), naming (max 3 points), short-term memory (max 5 points), attention (max 6 points), language (max 3 points), abstraction (max 2 points), and orientation (max 6 points). The scale consists of 14 standardized test items, with a total possible score of 30 points. A 1-point adjustment was added to the total score for participants with ≤12 years of formal education to correct for educational attainment bias. An adjusted total score < 26 was deemed cognitive decline. Adjusted total and subdomain scores were collected (Supplementary Tables S2, S8, S14).

### MMN

A 256-channel ERP cap (Net Amps 400, EGI, United States) was used. In the auditory oddball paradigm adopted in this study, the speech sounds/ba/and/da/were used as stimuli, and each block included 1,000 stimulations. The standard stimulus was/ba/, which was presented a total of 850 times (85%), and the deviant stimulus was/da/, which was presented a total of 150 times (15%). The paradigm parameters were consistent with those used in our previous study (Chen et al., 2022). The stimuli were controlled by Eprime 2.0 software (Psychology software tools, Inc., United States) and delivered through a loudspeaker placed 1 m in front of the subject’s head at a sound intensity of approximately 40 dB above the threshold of the worst ear or at a comfortable level (near both ears) under conditions without a hearing aid. Offline analysis was performed using Net Station 4.3 (EGI, United States) software. The recorded raw EEG data were processed as follows: filtering (0.1 ∼ 30 Hz), EEG segmentation (analysis time: −100 to + 600 ms), artifact removal (eye movement, eye opening, bad channel), bad electrode replacement, superposition average, reference electrode selection (using the nasal root reference electrode), and baseline correction with the pre-stimulus interval of −100 ms to 0 ms as the baseline. The peak negative values of MMN at the Fz electrodes in a defined time window were identified by a computer algorithm. The Fz electrode was selected for the analysis of MMN amplitude and latency for the following reasons: MMN is primarily generated by neural activity in the frontotemporal cortex, and is characterized by a fronto-central scalp distribution, with its maximal amplitude observed at the Fz electrode (Bissonnette et al., 2025). Yang et al. (2023)quantified MMN at midline frontocentral electrodes in older adults and demonstrated that Fz is a key midline electrode for detecting MMN alterations associated with cognitive decline.

### Functional connectivity analysis

GeoSource 3.0 software from the Net Station 4.3 analysis system (EGI, United States) was used. The finite difference model (FDM) was used for sLORETA analysis. We performed sLORETA analysis using EEG signals evoked by deviant stimuli. This occurs because the EEG signals evoked by deviant stimuli within the 100–300 ms time window, where mismatch negativity (MMN) is located, are closely associated with functions such as auditory processing, cognitive conflict detection, and memory trace updating. Tracing the source of EEG signals during this time window allows for the investigation of abnormal central nervous system activity in diseases related to MMN (McMackin et al., 2021). Cerebral cortical parcellation was performed using the Brodmann classification system (88 bilateral regions) (Hassan and Wendling, 2018), and functional connectivity was observed among the regions of interest (ROIs), including the bilateral STG (BA 22), PHG (BA 36), IFG (BA 46), and PCC (BA 23).

Neuroelectrophysiological data are susceptible to confounding effects from exogenous inputs and latent variables (e.g., indirect modulation from unrecorded brain regions). To address this limitation, additional auxiliary brain areas were considered to construct a multivariate analytical model, ensuring that inferences about causal relationships among target brain regions are not confounded by irrelevant factors. Three functional groups of auxiliary control regions were included to account for distinct confounding influences and validate pathway specificity: (1) DMN regions: medial prefrontal cortex (mPFC), angular gyrus (AG) (Su et al., 2025), and anterior cingulate cortex (ACC) (Leung et al., 2023), to control for global DMN fluctuations and intrinsic cognitive activity that may confound the causal relationships among core ROIs; (2) Frontal control region: dorsolateral prefrontal cortex (DLPFC), to account for general executive control and prefrontal modulation effects (Jaquerod et al., 2024); (3) Sensory and salience control regions: primary visual cortex (V1) (Javadzadeh and Hofer, 2022) and insula (INS, a core node of the salience network) (Zheng et al., 2025), to exclude non-specific widespread brain activation and interference from the salience network. All auxiliary control regions were incorporated into the multivariate analytical model to minimize confounding effects and ensure the specificity of the primary functional connectivity results (data with auxiliary control regions: Supplementary Tables S3, S5, S9, S11, S15, S17; additional PGC data without auxiliary control regions are provided for reference: Supplementary Tables S4, S6, S10, S12, S16, S18).

Initially, 2,447 dipoles were distributed across the cerebral cortex. The average dipole current density in each region was used as the representative dipole current density for that specific region. Signals were loaded via MATLAB R2018b software (MathWorks, USA), and the GCCA toolbox (Seth, 2010) was imported into the software to obtain nonlinear partial Granger causality (PGC) values between cerebral regions. Nonlinear PGC extends linear PGC to nonlinear systems, enabling robust causal inference in nonlinear neural dynamics (e.g., dipole current density fluctuations), which is suitable for event-related EEG analysis (Seth, 2010). In accordance with the calculation method proposed by Guo et al. (2008), the key procedures of partial Granger computation are as follows:

### Nonlinear autoregressive model for dipole current density time series

To quantify the directional, nonlinear causal influence between brain regions while accounting for confounding effects, we employed a nonlinear autoregressive modeling framework to compute PGC from dipole current density time series. The analysis proceeds in four key steps: (1) fitting restricted and full nonlinear autoregressive models; (2) estimating covariance matrices of prediction errors; (3) adjusting for confounders via conditional variance decomposition; and (4) calculating the final PGC index as the log ratio of residual variances.

Step 1: Nonlinear Autoregressive Models.

For any two regions of interest (ROIs) X(t) (target) and Y(t) (candidate causal region), with a third conditioning ROI Z(t) (confounder), we constructed two nested nonlinear autoregressive models using radial basis function (RBF) kernels to capture nonlinear dependencies:

1. Restricted Model (S): Predicts X(t) using only past values of X(t) and Z(t), excluding Y(t):


$$\left\{ \begin{array}{c} X(t)=\sum_{j=1}^{p}[w_{1j}.\Phi_{j}(X(t-j))+w_{2j}.\Phi_{j}(Z(t-j))]+u_{1t}\\ Z(t)=\sum_{j=1}^{p}[w_{3j}.\Phi_{j}(X(t-j))+w_{4j}.\Phi_{j}(Z(t-j))]+u_{2t} \end{array}\right.$$

2. Full Model (*Σ*): Predicts X(t) using past values of X(t), Y(t), and Z(t), incorporating the candidate causal region Y(t):


$$\left\{ \begin{array}{c} \begin{array}{l} X(t)=\sum_{j=1}^{p}[w_{5j}.\Phi_{j}(X(t-j))\\ +w_{6j}\;\Phi_{j}(Y(t-j)+w_{7j}.\Phi_{j}(Z(t-j))]+u_{3}(t) \end{array}\\ \begin{array}{l} Y(t)=\sum_{j=1}^{p}[w_{8j}.\Phi_{j}(X(t-j))\\ +w_{9j}\;\Phi_{j}(Y(t-j)+w_{10j}.\Phi_{j}(Z(t-j))]+u_{4}(t) \end{array}\\ \begin{array}{l} Z(t)=\sum_{j=1}^{p}[w_{11j}.\Phi_{j}(X(t-j))\\ +w_{12j}\;\Phi_{j}(Y(t-j)+w_{13j}.\Phi_{j}(Z(t-j))]+u_{5}(t) \end{array} \end{array}\right.$$

Model details:

X(t), Y(t), Z(t): Dipole current density time series of the target, candidate causal, and conditioning ROIs, respectively.

p: Optimal lag order (1–10 ms, corresponding to 1–10 time steps at 1,000 Hz sampling), determined individually for each participant using the Akaike Information Criterion (AIC).

$\Phi_{j}$ (.): RBF kernel function, defined as $\Phi_{j}(X(t-j)$ = exp. (− $\frac{{\left\|X(t-j)-{\bar{X}}_{j}\right\|}^{2}}{2\sigma_{x}^{2}}$ ) and $\Phi_{j}(Z(t-j)$ = exp. (− $\frac{{\left\|Z(t-j)-{\bar{Z}}_{j}\right\|}^{2}}{2\sigma_{z}^{2}}$ ). Here, ${\bar{X}}_{j}$ : Center of the j-th RBF kernel for X(t), set to the mean value of X(t-j) across all t. ${\bar{Z}}_{j}$ : Center of the j-th RBF kernel for Z(t), set to the mean value of Z(t-j). The kernel width was set to the within-ROI signal standard deviation, consistent with the default parameterization of the original nonlinear PGC framework (Guo et al., 2008; Marinazzo et al., 2008). Sensitivity analyses with 0.5 × and 1.5 × kernel widths yielded highly consistent results (effect size changes <5%), confirming the robustness of our findings (Supplementary Tables S37–S38).

$\sigma_{x}^{2}$ , $\;\sigma_{z}^{2}$ : Variance of X(t) and Z(t), respectively, calculated as $\sigma_{z}^{2}$ = $\frac{1}{N}$ $\sum_{t-1}^{N}{\left(X(t)-\mathrm{\mu x}\right)}^{2}$ (where μx is the mean of X(t).

$w_{1j}$ - $w_{4j}$ : Model coefficients, fitted via least-squares minimization of prediction error (residual sum of squares).

$u_{1t}$ , $\;u_{2t}$ : Prediction errors, representing unmodeled noise, exogenous inputs (e.g., background EEG artifacts), and latent variable influences.

j: Lag index (j = 1…, p), representing the j-th past time step (e.g., j = 1 corresponds to t-1, i.e., 1 ms before time t).

Step 2: Covariance Matrices of Prediction Errors.

From the residuals of the restricted and full models, we estimated two covariance matrices:

1. Restricted Model Covariance (S): Captures the variance–covariance structure of errors in the restricted model (without Y(t)):


$$S=(\begin{array}{cc} \mathrm{var}(u_{1}(t)) & \mathrm{cov}(u_{1}(t),u_{2}(t))\\ \mathrm{cov}(u_{2}(t),u_{1}(t)) & \mathrm{var}(u_{2}(t)) \end{array})=[\begin{array}{cc} S_{11} & S_{12}\\ S_{21} & S_{22.} \end{array}]$$

$S_{11}$ : Baseline prediction error variance of X(t) (without (Y(t)).

$S_{12}$ : Covariance between errors of X(t) and Z(t) (reflecting shared exogenous/latent confounders).

2. Full Model Covariance (*Σ*₁): Captures the variance–covariance structure of errors in the full model (with Y(t)):


$$\Sigma_{1}=(\begin{array}{cc} \mathrm{var}(u_{3}(t)) & \mathrm{cov}(u_{3}(t),u_{5}(t))\\ \mathrm{cov}(u_{5}(t),u_{3}(t)) & \mathrm{var}(u_{5}(t)) \end{array})=[\begin{array}{cc} \Sigma_{11} & \Sigma_{12}\\ \Sigma_{21} & \Sigma_{22} \end{array}]$$

$\Sigma_{11}$ : Prediction error variance of X(t) after incorporating Y(t).

$\Sigma_{12}$ : Covariance between errors of X(t) and Z(t) in the full model.

Step 3: Confounder Adjustment via Conditional Variance

To isolate the effect of Y(t) on X(t) while removing the confounding influence of Z(t), we computed conditional variances for both models:

Restricted Model Conditional Variance ( $R_{\mathrm{XX}|Z}^{\left(1\right)}$ )

Adjusts $S_{11}$ to remove confounding effects of Z(t) (shared exogenous/latent variables):


$$R_{\mathrm{XX}|Z}^{\left(1\right)}=S_{11}-S_{12}S_{22}^{-1}S_{21}$$

Full Model Conditional Variance ( $R_{\mathrm{XX}|Z}^{\left(2\right)}$ )

Adjusts $\Sigma_{11}$ to eliminate confounders in the full model: $R_{\mathrm{XX}|Z}^{\left(2\right)}=\Sigma_{11}-\Sigma_{12}\Sigma_{22}^{-1}\Sigma_{21}$

Step 4: Calculating the Nonlinear PGC Index

The PGC value quantifies the reduction in prediction error of X(t) after incorporating Y(t), adjusted for confounders. It is defined as the log ratio of the conditional variances:

$F1=\ln(\frac{S_{11}-S_{12}S_{22}^{-1}S_{21}}{\Sigma_{11}-\Sigma_{12}\Sigma_{22}^{-1}\Sigma_{21}}$ )

A positive F1 indicates that Y(t) improves the prediction of X(t) after controlling for Z(t), providing evidence of a nonlinear causal influence from Y to X.

A nonpositive F1 indicates no significant nonlinear causal influence from Y to X.

### Statistical analysis

All statistical analyses were performed using SPSS 22.0 (IBM Corp., Armonk, NY, USA). All tests were two-tailed with a prespecified significance level of *α* = 0.05. The Benjamini–Hochberg false discovery rate (FDR) method was uniformly applied for multiple comparison correction across all *post hoc* tests and correlation analyses (detailed statistical workflows, model validations, and complete test results are provided in Supplementary Tables S26–S40).

1 General statistical settings

Continuous variables are presented as means ± standard deviations for normally distributed data, or medians (interquartile range, IQR) for non-normally distributed data; categorical variables are presented as numbers (percentage). Normality and homogeneity of variance were assessed via Shapiro–Wilk and Levene’s tests, respectively (Supplementary Table S26-1).

2 Between-group comparisons

Consistent methods were applied for both lateralization (control, right/left-sided USNHL) and severity-stratified (control, moderate/severe USNHL) subgroup analyses.

- Cognitive function (MoCA): Non-normally distributed total and subdomain scores were analyzed via Kruskal–Wallis *H* test, with *post hoc* Dunn’s test and FDR correction (Supplementary Table S27).
- MMN parameters: Welch-James one-way ANCOVA (adjusted for age, sex, and education) was used for variables with violated homogeneity of variance (Supplementary Table S26-1). The homogeneity of regression slopes assumption was fully satisfied (Supplementary Table S26-1), with *post hoc* comparisons via Games–Howell test.

Directional functional connectivity (PGC): Random intercept-only mixed-effects ANCOVA was performed to test group, hemisphere, and Group × Hemisphere interaction effects on the four target pathways. This model accounted for intra-individual correlation of bilateral hemisphere data, with the optimal structure validated via likelihood ratio test (Supplementary Table S26-1). All models were adjusted for age, sex, and education, with separate analyses for lateralization and severity subgroups (Supplementary Table S28). Mauchly’s test confirmed sphericity of all within-subject factors, eliminating the need for degrees of freedom correction. Significant interactions were decomposed via two-way simple effect analysis, with post hoc Tukey’s HSD test and FDR correction (Supplementary Table S29). To explore potential stage-specific differences in directional coupling between early sensory processing and late cognitive integration, we further divided the MMN time window into two sub-epochs for exploratory analysis: an early sensory encoding phase (100–200 ms) and a late cognitive integration phase (200–300 ms). PGC values for core pathways were computed separately for each sub-window and compared across groups.

4 Correlation and predictive value analysis

Partial Spearman’s rank correlation (adjusted for age, sex, and education) was used to assess associations between PGC indices and MoCA scores. Hierarchical multiple linear regression was employed to verify the independent predictive value of core PGC indices for education-adjusted MoCA scores. All predictors were Z-score standardized to eliminate dimensional differences. Multicollinearity among predictors for hierarchical regression and mediation models was diagnosed using the variance inflation factor (VIF), with all VIF values < 2 confirming the absence of significant multicollinearity (detailed VIF results are presented in Supplementary Tables S26-2, S26-3). All core statistical assumptions were pre-tested and validated: basic analysis assumptions (Supplementary Table S26-1), detailed basic analysis assumptions for individual tests (Supplementary Table S26-1), hierarchical regression assumptions (Supplementary Table S26-2), and serial mediation assumptions (Supplementary Table S26-3).

Two nested models were constructed for each outcome: (1) Base Model (adjusted for age, sex, and education); and (2) Full Model (adding standardized left IFG → STG and left PHG → PCC PGC). Incremental variance explained (Δ*R*^2^) and squared semi-partial correlations (*sr*^2^) with 95% CIs were calculated to quantify predictor effects. Robust M-estimation regression was performed as a sensitivity analysis (Supplementary Table S33). FDR correction was applied for multiple comparisons across outcome models.

4 Mediation analysis and sensitivity analysis

All serial mediation analyses reported herein are exploratory associative analyses rather than causal tests. Given the cross-sectional study design, we cannot establish temporal precedence or causal directionalities among variables. Serial mediation analysis was re-fitted via a unified dual-pathway serial mediation model (PROCESS Macro Model 6), which simultaneously estimates the attention-domain (left IFG → STG PGC) and memory-domain (left PHG → PCC PGC) pathways, with 5,000 bias-corrected and accelerated bootstrap samples to assess indirect effects. All model assumptions were verified, with model fit meeting standard psychometric thresholds (full model outputs in Supplementary Table S32-1, S32-2, S32-3). Analyses were restricted to 60 unilateral SNHL patients aged 18–60 years to exclude age-related neurodegeneration and inter-group heterogeneity, with models adjusted for age and years of education. An additional sensitivity analysis was performed by adjusting the primary mixed-effects ANCOVA model for MoCA total score, confirming that PGC findings were independent of cognitive performance.

### Sample size and power analysis

The sample size was determined per retrospective case–control study design principles via *a priori* power analysis using PASS 15.0 software (NCSS, LLC, Kaysville, Utah, USA), strictly following the CONSORT guidelines for observational studies. Anchored to our primary outcome—the Group × Hemisphere interaction effect on core auditory-cognitive pathway directional functional connectivity tested by random intercept-only mixed-effects ANCOVA—we set a two-tailed *α* = 0.05, maximum *β* = 0.2 (corresponding to 80% statistical power), and an expected conservative large effect size of Cohen’s *f* = 0.4, consistent with prior peer-reviewed studies of SNHL-related auditory cortex functional connectivity(Chen et al., 2022; Gao et al., 2023). Accounting for an estimated 10% potential dropout rate, a sample of 30 participants per group (total *n* = 90) achieved > 0.99 statistical power for the primary outcome and > 0.90 power for secondary correlation and severity-stratified analyses. For the 60-patient mediation subsample, Monte Carlo power analysis for observed-variable serial mediation (Schoemann et al., 2017) demonstrated 83% power to detect the total indirect effect, with power for individual specific paths approaching the 80% threshold as expected for exploratory pathway analyses (average standardized *β* = 0.27 across significant paths, *α* = 0.05). All significant effects were supported by non-zero-crossing 95% BCa confidence intervals, meeting robustness standards for mechanistic observational studies. All participants had complete neuroimaging, electrophysiological, and cognitive data with no missing values (Supplementary Tables S34-1, S34-2, S35).

## Results

1 Baseline characteristics

No significant differences in age, sex, or years of education were observed across the control, right-sided USNHL, and left-sided USNHL groups (all *p* > 0.05, Table 1), nor between moderate and severe USNHL subgroups (all p > 0.05, Table 2); the two USNHL subgroups showed comparable affected ear PTA, contralateral ear PTA, and absolute hearing asymmetry (all *p* > 0.05, Supplementary Table S25), whereas the severe USNHL group had significantly greater hearing asymmetry than the moderate group did (*p* < 0.001, Table 2), and all baseline data satisfied the homogeneity of regression slopes assumption for subsequent covariance analyses.

2 Cognitive function (MoCA scores)

**Table 1:** Comparison of baseline characteristics and MoCA scores between the USNHL groups and the control group.

Variables	Right-sided SNHL group (*n* = 30)	Left-sided SNHL group (*n* = 30)	Control group (*n* = 30)	Test statistic	*p* value
Age (years, Mean ± SD)	47.4 ± 7.5	44.8 ± 6.7	45.79 ± 7.6	*F* = 0.96	*p* = 0.385
Sex (male/female, *n*)	15/15	14/16	17/13	*χ*^2^ = 0.62	0.66
Years of education (years, Mean ± SD)	13.2 ± 1.4	13.0 ± 1.2	13.3 ± 1.0	*F* = 0.58	0.73
Affected ear PTA, dBHL (Mean ± SD)	60.8 ± 11.9	61.4 ± 12.6	13.1 ± 1.3	*F* = 1124.3	<0.001
Contralateral ear PTA, dBHL (Mean ± SD)	16 ± 2	12 ± 2	13.9 ± 1.9	*F* = 124.37	<0.001
Hearing asymmetry difference (dB)	45.1 ± 11.2	49.3 ± 10.7	N/A	*t* = −1.50	0.138
MoCA Score (max = 30 points)	27 (25, 28)	28 (26, 28)	29 (28, 30)	*H* = 42.56	<0.001
Visuospatial/executive (max = 5)	5 (5, 5)	5 (5, 5)	5 (5, 5)	*H* = 0.00	1.00
Naming (max = 3)	3 (3, 3)	3 (3, 3)	3 (3, 3)	*H* = 0.00	1.00
Memory (max = 5)	3 (2, 4)	4 (3, 5)	4 (3, 5)	*H* = 28.3	<0.001
Attention (max = 6)	5 (4, 6)	6 (4, 6)	6 (6, 6)	*H* = 35.7	<0.001
Language (max = 3)	3 (3, 3)	3 (3, 3)	3 (3, 3)	*H* = 0.00	1.00
Abstraction (2)	2(2, 2)	2 (2, 2)	2 (2, 2)	*H* = 0.00	1.00
Orientation (6)	6(6, 6)	6 (6, 6)	6 (6, 6)	*H* = 0.00	1.00

*p < 0.05 vs control group. For the healthy control group, the affected ear PTA column presents the PTA of the right ear, and the contralateral ear PTA column presents the PTA of the left ear.

**Table 2:** Comparison of baseline characteristics and MoCA scores among the moderate USNHL group, severe USNHL group and the control group.

Variables	Control group (*n* = 30)	Moderate SNHL group (*n* = 29)	Severe SNHL group (*n* = 31)	Test statistic	*p* value
Age (years, Mean ± SD)	45.9 ± 7.6	45.2 ± 8.9	46.9 ± 8.2	*F* = 0.312	*p* = 0.733
Sex (male/female, *n*)	17/13	15/14	14/17	*χ*^2^ = 0.687	*p* = 0.709
Years of education (years, Mean ± SD)	13.3 ± 1.0	13.1 ± 1.3	13.0 ± 1.2	*F* = 0.601	*p* = 0.550
Affected ear PTA, dBHL (Mean ± SD)	13.1 ± 1.3	50.6 ± 7.8	71.8 ± 5.2	*F* = 1287.6	<0.001
Contralateral ear PTA, dBHL (Mean ± SD)	13.9 ± 1.9	14.0 ± 2.5	14.1 ± 2.7	*F* = 0.102	*p* = 0.903
Hearing asymmetry difference (dB)	N/A	36.7 ± 7.1	57.8 ± 5.9	*T* = −12.47	<0.001
MoCA score (max = 30 points)	29 (28, 30)	28 (27, 28)	26 (25, 27)	*H* = 58.23	<0.001
Memory (max = 5,)	4 (3, 5)	3 (3, 4)	3 (2, 4)	*H* = 32.45	<0.001
Attention (max = 6, median, IQR)	6 (6, 6)	5 (4, 6)	5 (4, 5)	*H* = 38.67	<0.001
Visuospatial/executive (max = 5)	5 (5, 5)	5 (5, 5)	5 (5, 5)	*H* = 0.00	1.00
Naming (max = 3)	3 (3, 3)	3 (3, 3)	3 (3, 3)	*H* = 0.00	1.00
Language (max = 3)	3 (3, 3)	3 (3, 3)	3 (3, 3)	*H* = 0.00	1.00
Abstraction (max = 2)	2 (2, 2)	2 (2, 2)	2 (2, 2)	*H* = 0.00	1.00
Orientation (max = 6)	6 (6, 6)	6 (6, 6)	6 (6, 6)	*H* = 0.00	1.00

Education-adjusted MoCA scores demonstrated significant cognitive alterations in patients with USNHL relative to healthy controls (Tables 1, 2; Supplementary Table S27). Both right-sided and left-sided USNHL patients had significantly lower total MoCA scores, memory subscores and attention subscores than controls, and no significant differences were detected between the two USNHL subgroups in any cognitive measure. Severity-stratified analysis identified a domain-specific graded pattern of cognitive decline, with total MoCA scores and attention subscores decreasing progressively as hearing loss worsened from moderate to severe, while memory subscores were comparably decreased in both moderate and severe USNHL patients compared with controls but did not differ significantly between the two severity groups. No significant intergroup variations were found in visuospatial/executive function, naming, language, abstraction or orientation across all comparisons (all *p* = 1.00, Supplementary Table S27).

3 Mismatch negativity (MMN) parameters

**Figure 1:**
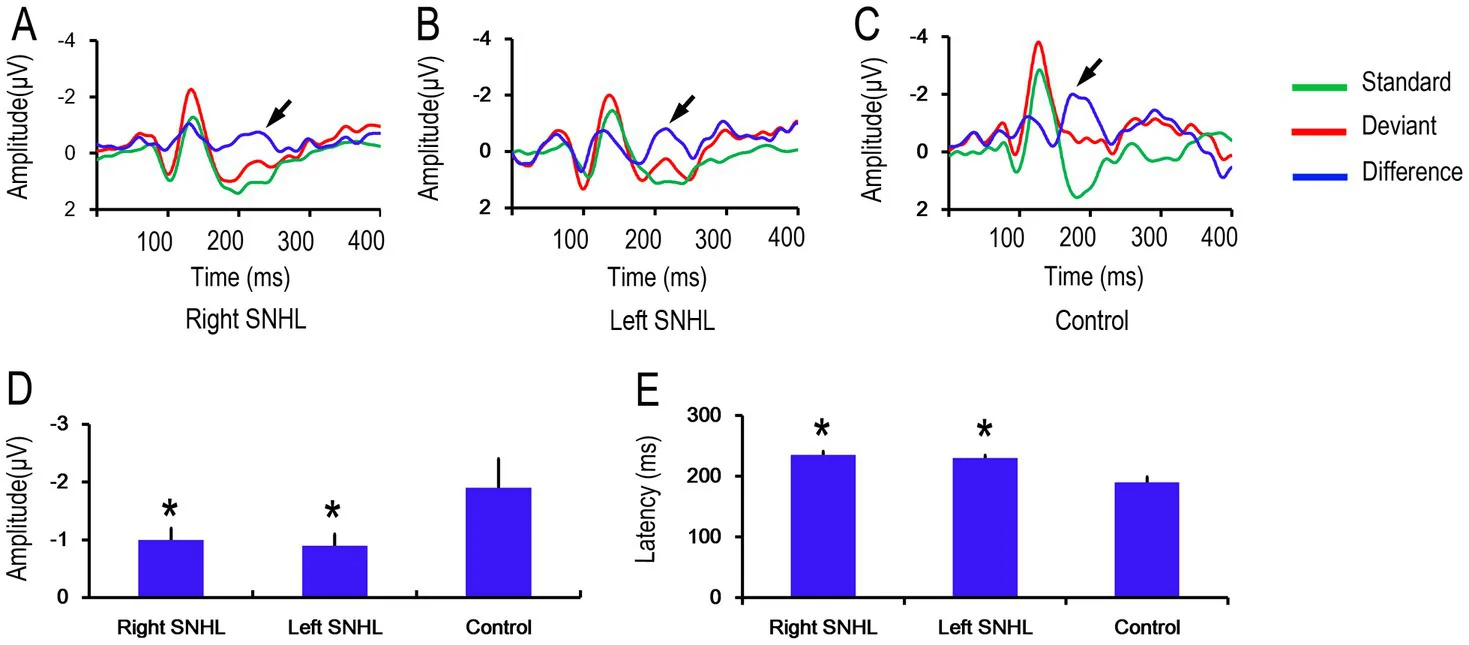
Grand-averaged MMN waveforms and group comparisons of MMN components at Fz in USNHL patients and controls. **(A–C)** Grand-averaged ERP waveforms at Fz for right-sided USNHL **(A)**, left-sided USNHL **(B)**, and controls **(C)**. Three waveforms: Standard (neural responses to repeated auditory stimuli), deviant (responses to infrequent deviant stimuli), and difference (deviant minus standard, to isolate MMN). The black arrow indicates the MMN peak. **(D, E)** MMN group comparisons: **(D)** amplitude, **(E)** latency. Error bars represent the standard error of the mean. *p < 0.001 vs. control group (post-hoc test). Grand-averaged MMN waveforms at Fz and group comparisons of MMN amplitude/latency in USNHL patients and controls. Panels A–C show standard, deviant, and difference ERP waveforms (right/left-sided USNHL, controls); the black arrow denotes the MMN peak. Panels D–E: USNHL groups exhibit lower MMN amplitude and prolonged latency vs. controls; Error bars represent SEM.

After adjusting for age, sex, and years of education, Welch-James ANCOVA revealed robust group differences in MMN indices at the Fz electrode (all *p* < 0.001, Figures 1A–E); both USNHL groups exhibited significantly reduced MMN amplitude (Figures 1A,B,D) and prolonged MMN latency (Figures 1A,B,E) compared with controls (Figure 1C,D,E) (all *p* < 0.001), with no significant differences between the two USNHL subgroups or between moderate and severe USNHL subgroups (all *p* > 0.05) (Supplementary Tables S1, S7, S13, Figure S1A–D).

4 Directional functional connectivity (PGC)

**Figure 2:**
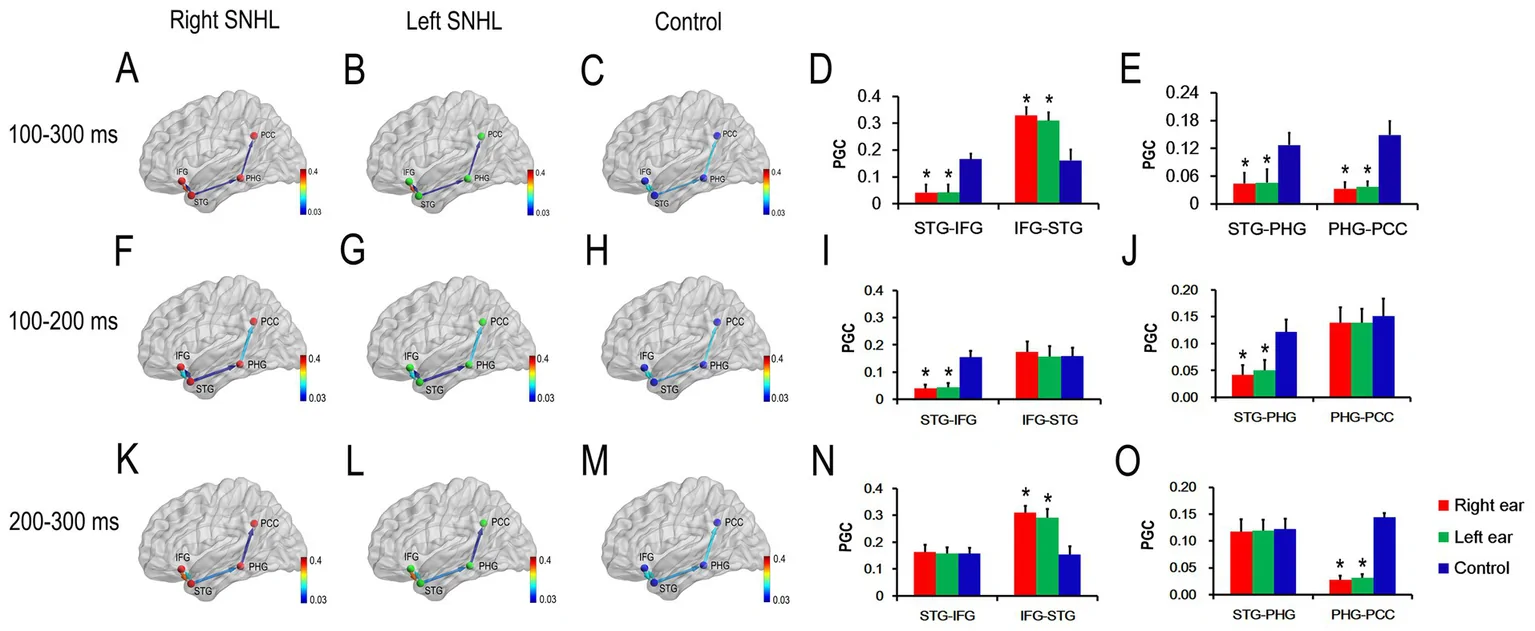
Time-resolved left-hemisphere PGC in USNHL patients stratified by affected ear side, across three post-stimulus time windows (100–300 ms, 100–200 ms, 200–300 ms). **(A–C, F–H, K–M)** 3D brain renderings of significant connections in right-sided USNHL, left-sided USNHL, and controls. Arrows indicate direction; color bars encode PGC magnitude (warmer = stronger). **(D, E, I–J, N–O)** Bar graphs comparing PGC values for STG–IFG and STG → PHG → PCC pathways. Data are mean ± SD. Red: Right USNHL; Green: Left USNHL; Blue: Controls. *p < 0.001 vs. controls. PGC, partial Granger causality; STG, superior temporal gyrus; IFG, inferior frontal gyrus; PHG, parahippocampal gyrus; PCC, posterior cingulate cortex. Three-row panel figure showing time-resolved left-hemisphere PGC in right-sided USNHL, left-sided USNHL, and controls across three post-stimulus time windows (100–300 ms, 100–200 ms, 200–300 ms). 3D brain renderings display significant directed connections; bar graphs compare PGC values for STG–IFG and STG → PHG → PCC pathways. Asterisks indicate *p* < 0.001 vs. controls.

**Figure 3:**
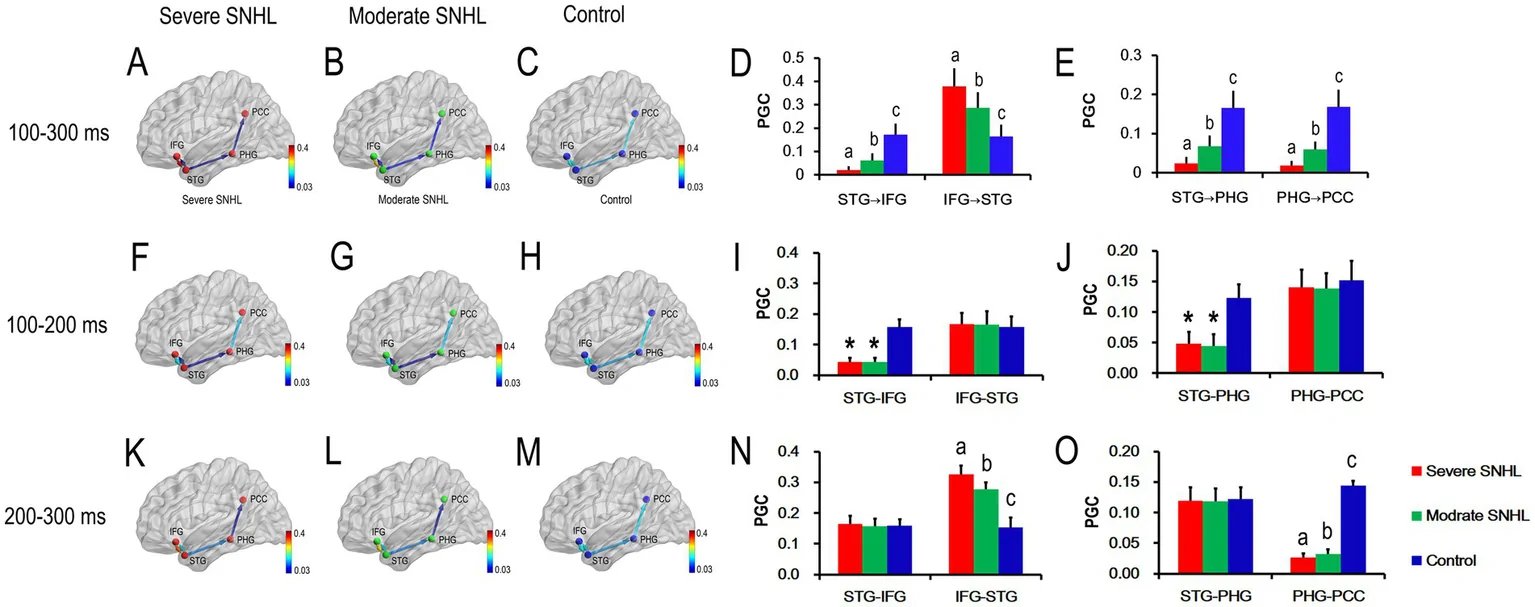
Time-resolved left-hemisphere PGC in USNHL patients stratified by hearing loss severity. Layout and time windows are identical to Figure 2. **(A–C, F–H, K–M)** 3D brain renderings of severe USNHL, moderate USNHL, and controls. Arrows and color bars as in Figure 2. **(D, E, I, J, N, O)** Bar graphs comparing core pathway PGC values. Data are mean ± SD. Red: Severe USNHL; Green: Moderate USNHL; Blue: Controls. Early window: *p < 0.001 vs. controls. Full and late windows: Different letters (a–c) indicate significant pairwise differences (*p* < 0.05). PGC, partial Granger causality; STG, superior temporal gyrus; IFG, inferior frontal gyrus; PHG, parahippocampal gyrus; PCC, posterior cingulate cortex. Three-row panel figure showing time-resolved left-hemisphere PGC in USNHL stratified by hearing loss severity (severe, moderate) and controls across three time windows. 3D brain renderings display significant connections; bar graphs compare core pathway PGC values. Asterisks and letter markers (a–c) indicate significant between-group differences.

Separate mixed-effects ANCOVA models were used for lateralization and severity-stratified subgroup analyses of directional functional connectivity (PGC), with all models including hemisphere as a within-subject factor, a random intercept for each subject, and adjustment for age, sex, and education; in lateralization subgroups, all four target PGC pathways (STG → IFG, IFG → STG, STG → PHG, PHG → PCC) showed significant main effects of group, hemisphere, and robust Group × Hemisphere interaction effects (all *p* < 0.001), whereas in severity-stratified subgroups, all four target PGC pathways exhibited significant group main effects (all *p* < 0.001) and robust Group × Hemisphere interaction effects (all *p* < 0.001) (Supplementary Table S28); *post hoc* simple effect analyses revealed that statistically significant between-group differences in PGC values were exclusively localized to the left hemisphere (all *p* < 0.001, Cohen’s *d* > 1.8), with no significant intergroup differences detected in the right hemisphere (all p > 0.05) (Supplementary Table S29, Figure S2 A–C); compared with controls (Figure 2C), USNHL patients showed significantly increased left IFG → STG PGC and reduced left STG → IFG, STG → PHG, and PHG → PCC PGC (all *p* < 0.001) (Figures 2A,B,D,E), with no significant differences between right-sided and left-sided USNHL subgroups (Table 3); left hemisphere abnormalities of all four pathways were more pronounced in severe USNHL patients (Figure 3A) than in moderate USNHL patients (Figure 3B) (all *p* < 0.001) (Table 4), with a graded pattern of abnormality across control (Figure 3C), moderate, and severe subgroups (Figures 3D,E) (Supplementary Table S29); USNHL patients showed a consistent left hemispheric lateralization pattern: for right-sided USNHL, all bottom-up pathways (STG → IFG, STG → PHG, PHG → PCC) had significantly lower PGC values in the contralateral (left) hemisphere, whereas the top-down IFG → STG pathway had significantly higher PGC values in the contralateral hemisphere; for left-sided USNHL, the opposite pattern was observed, with bottom-up pathways showing lower PGC values in the ipsilateral (left) hemisphere and the top-down pathway showing higher PGC values in the ipsilateral hemisphere (all *p* < 0.001) (Supplementary Table S29); sensitivity analysis with additional adjustment for MoCA total score confirmed the robustness of these core PGC findings independent of cognitive performance (all core effects remained significant, *p* < 0.001) (Supplementary Table S33). Complementarily, linear Granger causality models detected no significant between-group differences across any of the four pathways (all *p* > 0.05; Supplementary Table S40-1 to S40-3), indicating that the observed connectivity alterations in USNHL are primarily driven by nonlinear neural dynamics captured by the RBF-kernel PGC approach.

**Table 3:** Group comparisons of left-hemisphere PGC values stratified by lesion lateralization (100–300 ms post-stimulus).

PGC Pathway	HC Mean ± SD	R-USNHL Mean ± SD	L-USNHL Mean ± SD	*F*	*p*	HC vs. R-USNHL (95% CI)	HC vs. L-USNHL (95% CI)	R-USNHL vs. L-USNHL (95% CI)
STG → IFG	0.164 ± 0.022	0.044 ± 0.023	0.041 ± 0.019	143.85	<0.001	0.120 (0.108, 0.133)*	0.123 (0.110, 0.136)*	0.003 (−0.010, 0.016)
IFG → STG	0.161 ± 0.045	0.330 ± 0.069	0.317 ± 0.059	183.72	<0.001	−0.169 (−0.196, −0.142)*	−0.156 (−0.183, −0.129)*	0.013 (−0.014, 0.040)
STG → PHG	0.127 ± 0.026	0.049 ± 0.023	0.047 ± 0.020	119.74	<0.001	0.078 (0.068, 0.088)*	0.080 (0.070, 0.090)*	0.002 (−0.008, 0.012)
PHG → PCC	0.149 ± 0.031	0.033 ± 0.015	0.038 ± 0.013	247.69	<0.001	0.116 (0.106, 0.126)*	0.111 (0.101, 0.121)*	−0.005 (−0.015, 0.005)

HC, healthy controls; USNHL, unilateral sensorineural hearing loss; PGC, partial Granger causality; STG, superior temporal gyrus; IFG, inferior frontal gyrus; PHG, parahippocampal gyrus; PCC, posterior cingulate cortex; CI, confidence interval; FDR, false discovery rate. *p < 0.05.

**Table 4:** Group comparisons of left-hemisphere PGC values stratified by hearing loss severity (100–300 ms post-stimulus).

PGC Pathway	HC Mean ± SD	Moderate USNHL Mean ± SD	Severe USNHL Mean ± SD	*F*	*p*	HC vs. moderate (95% CI)	HC vs. severe (95% CI)	Moderate vs. severe (95% CI)
STG → IFG	0.164 ± 0.022	0.052 ± 0.018	0.021 ± 0.012	145.12	<0.001	0.112 (0.101, 0.124)*	0.143 (0.131, 0.155)*	0.031 (0.018, 0.044)*
IFG → STG	0.161 ± 0.045	0.256 ± 0.033	0.347 ± 0.031	189.15	<0.001	−0.095 (−0.118, −0.072)*	−0.186 (−0.209, −0.163)*	−0.091 (−0.112, −0.070)*
STG → PHG	0.127 ± 0.026	0.068 ± 0.019	0.035 ± 0.015	120.89	<0.001	0.059 (0.049, 0.069)*	0.092 (0.081, 0.103)*	0.033 (0.022, 0.044)*
PHG → PCC	0.149 ± 0.031	0.048 ± 0.010	0.011 ± 0.006	248.92	<0.001	0.101 (0.090, 0.112)*	0.138 (0.127, 0.149)*	0.037 (0.027, 0.047)*

HC, healthy controls; USNHL, unilateral sensorineural hearing loss; PGC, partial Granger causality; STG, superior temporal gyrus; IFG, inferior frontal gyrus; PHG, parahippocampal gyrus; PCC, posterior cingulate cortex; CI, confidence interval; FDR, false discovery rate. *p < 0.05.

In the early 100–200 ms window (Figures 2F–J), significant group differences were confined to bottom-up auditory pathways. Left STG → IFG and STG → PHG connectivity was markedly reduced in both RS-SNHL and LS-SNHL groups relative to HC (both *p* < 0.001) (Figures 2I–J), with no inter-side difference. Top-down IFG → STG and PHG → PCC connectivity did not differ across groups (Supplementary Table S39-3). Severity-stratified analyses revealed a stepwise gradient for both STG → IFG and STG → PHG (Figures 3F–J) (severe < moderate < HC; both *p* < 0.001, Supplementary Table S39-4). In the late 200–300 ms window (Figure 2K–O), the pattern reversed. Left IFG → STG connectivity was significantly elevated in SNHL patients (*p* < 0.001) (Figures 2O), whereas PHG → PCC connectivity was reduced (*p* < 0.001); both pathways showed severity-dependent gradients (Figure 3K–O) (HC < moderate < severe for IFG → STG; severe < moderate < HC for PHG → PCC, Supplementary Table S39-4). Bottom-up pathways (STG → IFG, STG → PHG) no longer differed among groups (Supplementary Table S39-3).

5 Correlation between PGC indices and MoCA scores

**Figure 4:**
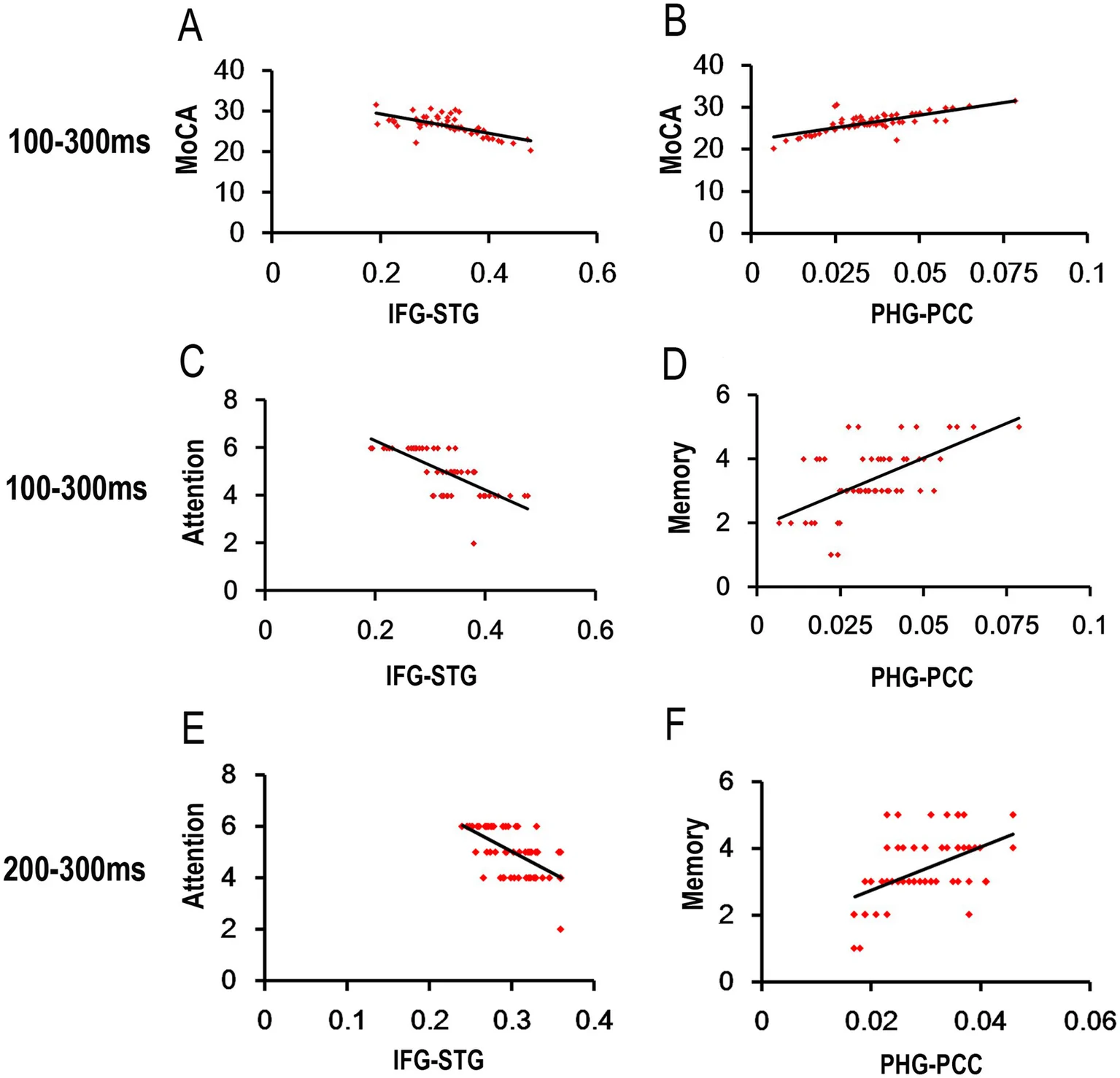
Scatter plots illustrating the associations between left-hemisphere PGC indices and MoCA scores in 60 USNHL patients. **(A, B)** Correlations of left IFG → STG and left PHG → PCC PGC with MoCA total scores in the 100–300 ms post-stimulus window. **(C, D)** Corresponding correlations with MoCA attention and memory subscores in the 100–300 ms window. **(E, F)** Correlations with MoCA attention and memory subscores restricted to the late window (200–300 ms). Scatter plots showing correlations between left-hemisphere PGC indices and MoCA scores in 60 USNHL patients. Panels display associations of left IFG → STG and left PHG → PCC PGC with MoCA total, attention, and memory subscores in the full 100–300 ms window and the late 200–300 ms window. Solid lines represent fitted regression trends.

In the USNHL cohort (*n* = 60), partial Spearman’s rank correlation analysis (adjusted for age, sex, and education) showed that left IFG → STG PGC was negatively correlated with MoCA total score (*r* = −0.59, *p* < 0.001) (Figures 4A) and specifically with the attention subdomain score (*r* = −0.66, *p* < 0.001) (Figures 4C), whereas left PHG → PCC PGC was positively correlated with MoCA total score (*r* = 0.57, *p* < 0.001) (Figure 4B) and specifically with the memory subdomain score (*r* = 0.71, *p* < 0.001) (Figure 4D); no significant correlations were observed between any right-hemisphere PGC pathways and MoCA scores (all *p* > 0.05), and Steiger’s Z test confirmed strict pathway-cognitive domain specificity (all *p* < 0.001) (Supplementary Tables S29, S30, Figure S2 D-E). Late-window correlation analyses (Figures 4E,F) replicated the domain-specific association pattern: IFG → STG connectivity was selectively correlated with MoCA attention subscores (Figure 4 E) (Spearman’s *ρ* = −0.633, FDR-corrected *p* = 0.002), and PHG → PCC with memory subscores (Figures 4F) (*ρ* = 0.477, FDR-corrected *p* = 0.002). No cross-domain correlations survived FDR correction (Supplementary Table S39-5).

6 Independent predictive value of PGC indices for cognitive function

Hierarchical multiple linear regression (all core assumptions satisfied, Supplementary Table S26-2) showed that after controlling for demographic covariates, left IFG → STG and left PHG → PCC PGC together explained 59.2% of the variance in MoCA total score (Δ*R*^2^ = 0.516, *p* < 0.001), wherein left IFG → STG PGC was an independent negative predictor of MoCA total score (*β* = −0.541, *p* < 0.001) and specifically of attention subdomain score (*β* = −0.612, *p* < 0.001), and left PHG → PCC PGC was an independent positive predictor of MoCA total score (*β* = 0.527, *p* < 0.001) and specifically of memory subdomain score (*β* = 0.634, *p* < 0.001); domain-specific analyses confirmed that left IFG → STG PGC only predicted attention function, whereas left PHG → PCC PGC only predicted memory function, and robust M-estimation regression validated the stability of these findings (Supplementary Table S31).

7 Serial mediation analysis

As an exploratory mechanistic analysis, we fitted a unified dual-pathway serial mediation model to examine whether MMN alterations and PGC abnormalities sequentially mediate the association between hearing loss severity and cognitive decline. The model demonstrated excellent fit to data from 60 USNHL patients aged 18–60 years (Supplementary Table S32-1 to S32-3). Hearing loss severity (PTA) exerted a significant negative total effect on MoCA scores. The total indirect effect via the two neural pathways accounted for 34.4% of the total association (*B* = −0.011, *p* < 0.01), whereas the direct PTA–MoCA association did not reach statistical significance after inclusion of the serial mediators (*p* = 0.058; 95% BCa confidence interval includes zero), indicating that the observed PTA–MoCA relationship is predominantly statistically carried by the MMN latency – cortical connectivity mediator pathways rather than a single direct pathway. Domain-specific decomposition revealed two parallel pathways with distinct effect magnitudes. The attention-domain pathway (left IFG → STG PGC) explained 11.9% of the total association, and the memory-domain pathway (left PHG → PCC PGC) accounted for a larger 22.5%. Notably, only MMN latency–mediated serial chains reached statistical significance in both pathways; all MMN amplitude–mediated indirect effects showed non-significant bootstrap confidence intervals crossing zero. This pattern collectively suggests that prolonged MMN latency, rather than reduced MMN amplitude, may serve as the core electrophysiological mediator linking auditory deprivation severity to disrupted frontotemporal attention and medial temporal–parietal memory network connectivity, and subsequent cognitive decline in USNHL patients.

## Discussion

### Changes in cognitive function in patients with USNHL

We found that the MoCA score of patients with USNHL was reduced. The MoCA score can reflect neural activities corresponding to specific cognitive processing mechanisms and is a routine indicator for evaluating cognitive levels in clinical practice (Doan et al., 2021). Therefore, the lower MoCA score in patients with USNHL than in the controls reflects a decline in the cognitive function of patients with USNHL. Using resting-state EEG and sLORETA, Gao et al. (2023) showed that patients with SNHL have reduced connectivity between the superior frontal gyrus and cingulate gyrus, associated with decreased MoCA scores. They hypothesized that impaired auditory input prompts SNHL patients to mobilize cognitive cortices (frontal gyrus for attention regulation; cingulate gyrus for cognitive resource allocation) to process auditory information. This compensation occupies resources dedicated to MoCA-assessed cognitive functions (e.g., memory, attention), potentially contributing to lower cognitive scores. The adjusted total MoCA scores of our USNHL cohort showed a median range of 27 to 28, with a subset of patients presenting with early subtle cognitive decline (adjusted total MoCA score < 26), indicating that USNHL can induce early mild cognitive decline in adults. Stratified analysis by USNHL severity further revealed that patients with severe USNHL had lower MoCA scores. Specifically, patients with severe USNHL exhibited significantly lower attention subdomain scores than those with moderate USNHL did, whereas no significant difference was observed in memory subdomain scores between the two subgroups. This finding suggests that attention function is more susceptible to the severity of auditory deprivation, potentially serving as an earlier and more sensitive marker of cognitive decline than memory function in patients with USNHL. This graded association suggests that the severity of peripheral auditory deprivation may modulate the extent of central neural network reorganization, which in turn maps to the degree of cognitive decline in this population. We subsequently explored the potential mechanism using ERP.

### Changes in MMN in patients with SNHL

We found decreased MMN amplitude in patients with USNHL, which reflects the stability of auditory memory traces in the brain. Pei et al. (2020) demonstrated significantly lower MMN amplitude in patients with AD and amnestic mild cognitive decline than in healthy controls, suggesting that reduced MMN amplitude indicates impaired auditory working memory accuracy. Yang et al. (2023) reported that decreased MMN amplitude in the elderly correlates with changes in multiple cognitive functions (memory, language, attention), noting that abnormal MMN amplitude may reflect overall cognitive decline. We also observed prolonged MMN latency in USNHL patients. This is because MMN latency reflects the central auditory system’s automatic recognition speed for standard and deviant sounds, which depends on the synergy of cognition-related brain regions (frontal lobe, PHG). The frontal lobe is involved in rapid auditory processing and difference detection, while the PHG supports auditory memory and cognitive integration. Early cognitive decline alters the function/structure of these regions, reducing auditory signal processing efficiency and manifesting as prolonged MMN latency (Yang et al., 2023). Thus, our results suggest that USNHL patients exhibit altered central cognitive activity. To explore the underlying mechanism, we analyzed these changes using functional connectivity methods.

### Mutual influence between the STG and IFG in patients with USNHL

We observed reduced functional connectivity from the left STG to the left IFG in patients with SNHL. Furthermore, patients with severe USNHL exhibited significantly weaker STG-to-IFG functional connectivity than those with moderate USNHL. The BA22 region of the STG, an auditory association cortex, is responsible for advanced auditory processing, including semantic association of auditory signals and cross-modal integration (e.g., audition-language connections), and contributes to language perception and the conversion of pure auditory signals to language-related signals (Trebuchon et al., 2021). Using task-state EEG and sLORETA, Madashetty et al. (2024) found significantly reduced neural activity in the STG-containing temporal lobe of age-related hearing loss (ARHL) patients during working memory tasks. They attributed this insufficient activation to impaired STG function as an auditory information processing center, hindering effective auditory signal integration and transmission to other brain regions. Larsen et al. (2024) analyzed brain functional connectivity using the auditory oddball paradigm and dynamic causal modeling, finding reduced STG influence on the IFG in cognitively impaired patients. They noted that effective STG-to-IFG signal input enables the IFG to accurately distinguish expected from actual auditory stimuli, optimizing auditory sequence learning. In cognitively impaired patients, insufficient STG transmission of auditory stimulus deviation information to the IFG impairs the IFG’s ability to generate accurate top-down predictive regulatory signals, disrupting deviant sound identification and reducing individual sensitivity to sensory environment changes (e.g., difficulty adjusting processing strategies for new auditory information). Therefore, our results indicate that the reduced information transmission from the STG to the IFG in patients with USNHL is associated with changes in the advanced cognitive activities of the IFG.

Beyond these task-evoked directional changes, converging resting-state evidence indicates that IFG reorganization in USNHL may be an intrinsic network feature. Zhang et al. (2018) reported disrupted whole-brain connectivity across higher-order cognitive networks in long-term USNHL and attributed enhanced left IFG hub function to cross-modal plasticity, while Qiao et al. (2022) found that IFG ALFF increased with worsening auditory performance in patients with disease duration over 2 years. These at-rest observations, obtained without any auditory task, suggest that patients intrinsically reallocate frontal cognitive resources after losing binaural input. Consistent with this endogenous remodeling, we found that the PGC of the left IFG–STG increased after USNHL. Patients with severe USNHL exhibited significantly higher PGC values in this pathway than those with moderate USNHL. This PGC alteration was negatively correlated with MoCA attention subscale scores, suggesting that the increased IFG–STG connectivity in SNHL patients is closely associated with attention decline. Chen et al. (2022) used event-related EEG and sLORETA, reporting that reduced influence of the temporal auditory cortex on the IFG in patients with USNHL was accompanied by enhanced IFG influence on the temporal auditory cortex. They suggested this enhanced IFG regulation helps the auditory cortex process limited auditory signals more efficiently to maintain basic auditory function; however, excessive IFG inhibition in the auditory cortex may further interfere with deviant auditory signal processing, impair auditory memory formation, and ultimately be associated with reduced speech recognition and delayed speech comprehension. Larsen et al. (2024) reported enhanced IFG influence on the STG in individuals with a high familial risk of schizophrenia and bipolar disorder, suggesting excessive strengthening of this connection may precede cognitive alterations, reflecting excessive brain regulation in pre-attentive processing and potential cognitive network imbalance. Moreover, Mano et al. (2022) reported a negative correlation between frontal lobe functional connectivity and both cognitive and attention scores in patients with Parkinson’s disease–related mild cognitive decline. As a core hub mediating attention, executive function and memory encoding, the frontal lobe relies on efficient functional cooperation with the parietal lobe (information integration) and temporal lobe (language and memory storage) to complete cognitive tasks. Excessively or abnormally enhanced functional connectivity may disrupt normal cognitive processing (e.g., reduced information transmission efficiency, redundant neural signals), ultimately accounting for the negative correlation between elevated frontal lobe connectivity and decreased attention scores. The negative correlation between IFG–STG functional connectivity and both total MoCA scores and its attention subscale scores suggests that impaired IFG–STG connectivity may compromise attention and associated cognitive functions in patients with USNHL.

The time-window decomposition provides further temporal resolution to this bidirectional alteration pattern. Specifically, STG → IFG hypo-connectivity was most prominent in the 100–200 ms early sensory-encoding window, whereas IFG → STG hyper-connectivity emerged selectively in the 200–300 ms late cognitive window. This temporal dissociation is consistent with the interpretation that impaired bottom-up afferent transmission occurs during initial acoustic feature analysis, and that frontal compensatory recruitment appears to be engaged during the subsequent evaluative and attention-regulating stage. Furthermore, within the late 200–300 ms window, the strength of IFG → STG connectivity was selectively and negatively correlated with attention subscale scores, a pattern consistent with the full-window analysis. This staged pattern aligns with evidence that MMN latency prolongation reflects an early pre-attentive auditory processing alteration (Wang et al., 2022) and that auditory cortical processing differences are correlated with downstream cognitive changes in hearing loss (Gao et al., 2023), whereby frontal top-down compensation may become cognitively demanding and relate to reduced attentional performance.

### Relationships between the STG, PHG and PCC in patients with USNHL

We found weakened influence of the left STG–PHG in patients with USNHL. Patients with severe USNHL exhibited significantly weaker STG-to-PHG functional connectivity than those with moderate SNHL did. The STG conveys processed auditory information to the PHG for memory integration and meaning elaboration, endowing auditory information with memory attributes and associative meanings while supporting auditory tasks (e.g., sound recognition, memory retrieval) (Li et al., 2025). Li et al. (2025) reported that congenital hearing impairment significantly reduced auditory cortex (A1/A2) activation in affected children. These findings suggest that peripheral auditory pathway defects impede normal transmission of auditory information to the auditory cortex, reducing information delivery efficiency to the PHG and manifesting as decreased functional connectivity. Chen et al. (2022) reported reduced influence of the temporal auditory cortex on the PHG in patients with USNHL. They suggested that USNHL patients exhibit reduced PHG gray matter volume, decreased PHG neuron number/density, and diminished PHG spontaneous activity, resulting in generally weakened connections between the PHG and other auditory-related brain regions. Thus, the PHG fails to effectively receive and process auditory cortex-transmitted information, further weakening temporal auditory cortex-PHG functional connectivity, which may contribute to memory-related cognitive alteration in USNHL patients.

Converging USNHL-specific resting-state evidence indicates that PCC involvement is an intrinsic network alteration rather than a purely task-evoked phenomenon. Using rs-fMRI, Yang et al. (2014) reported reduced gray matter volume and ALFF in the bilateral PCC of USNHL patients, attributing these changes to the propagation of chronic auditory-cortex deprivation along long-range auditory–PCC projections; Qiao et al. (2022) further observed that PCC ALFF increased with disease duration, suggesting that an initial hypoactive state gives way to progressive plastic remodeling as deprivation persists. We further found reduced influence of the left PHG–PCC in patients with USNHL. Patients with severe USNHL showed significantly lower PGC values in this pathway than those with moderate USNHL. This PGC was positively correlated with MoCA memory subscale scores, suggesting that decreased PHG–PCC functional connectivity is closely associated with memory decline in USNHL patients. The PCC, a core node of the DMN, is mainly involved in memory encoding, storage, and retrieval, and is crucial for cognitive function; the PHG and PCC jointly mediate cognitive processes related to memory integration and self-awareness (Jiang et al., 2023). Li et al. (2025) used resting-state EEG and sLORETA and found weakened PHG–PCC connectivity in children with congenital SNHL. They proposed that auditory deprivation reduces overall functional connectivity of the salience network, central executive network, and DMN, further disrupting external regulatory signals required for PHG–PCC connections and exacerbating connectivity abnormalities. Using resting-state fMRI combined with Granger causality analysis, Jiang et al. (2023) found significantly lower PHG-to-PCC Granger causality values in temporal lobe epilepsy patients with cognitive decline than in healthy controls. They inferred that PHG-to-PCC information transmission is weakened, reflecting impaired information interaction between DMN core nodes that may affect cognitive processes. Using resting-state fMRI, Wei et al. (2025) discovered that when the functional connectivity between the PHG and PCC was weakened, the memory information processed by the PHG could not be effectively transmitted to the PCC for integration, which was associated with impaired memory function. This domain-specific correlation not only corroborates the functional role of the PHG-PCC pathway in episodic memory processing but also provides neurophysiological evidence for memory impairment in patients with USNHL.

Intriguingly, time-window analysis revealed a sequential dissociation along this auditory–memory axis. STG → PHG afferent reduction was most pronounced in the early 100–200 ms sensory window, whereas PHG–PCC connectivity impairment predominated in the 200–300 ms late cognitive window. This temporal offset is compatible with a putative serial propagation framework that the degraded auditory signals may fail to efficiently reach the PHG during early encoding, and this upstream insufficiency could be followed by secondary disruption of the PHG–PCC memory integration circuit during the later consolidation stage. In parallel, PHG → PCC connectivity in the 200–300 ms window was positively and selectively related to memory subscale scores, mirroring the association observed in the full 100–300 ms window. This concordance suggests that the late integration window is functionally relevant for memory performance, and that PHG–PCC circuit dysfunction during this stage may contribute to the memory alterations observed in SNHL patients. Converging evidence has indicated altered effective connectivity between PCC and PHG within the default mode network in cognitively impaired populations (Jiang et al., 2023), and the PHG has been described as a key cognitive-regulatory DMN node across multiple neurological conditions (Wei et al., 2025). The stepwise temporal dissociation thus provides dynamic correlational evidence compatible with a cascading disruption along the auditory–memory axis.

### MMN as a mediator of cognitive decline

Serial mediation analyses further elaborated this potential sequential associative framework. First, the link between hearing loss severity and cognitive decline was accounted for by significant serial indirect pathways involving MMN abnormalities and subsequent left-hemispheric PGC disturbances. This mediation pattern indicates that peripheral auditory deprivation is associated with cognitive differences through a multi-step central neurophysiological cascade. Second, two distinct, domain-specific serial pathways were identified, which map to the cognitive subdomain deficits observed in our cohort. The first pathway centers on attention-related cognitive function: hearing loss severity was linked to cognitive decline through sequential changes in prolonged MMN latency and subsequent enhancement of left IFG → STG connectivity. This aligns with our prior finding that left IFG → STG PGC was specifically associated with MoCA attention subscale scores, and supports the notion that MMN latency prolongation tends to be associated with deficits in both bottom-up and top-down auditory attention (Wang et al., 2022), which triggers compensatory strengthening of left frontotemporal (IFG → STG) auditory-attention network connectivity; however, such maladaptive reorganization excessively occupies cognitive resources and is ultimately linked to attention impairment in SNHL patients (Gao et al., 2023). The second pathway relates to memory-related cognitive function: hearing loss severity was associated with cognitive decline through sequential changes in prolonged MMN latency and subsequent weakening of left PHG → PCC connectivity. This mirrors our observation that left PHG → PCC PGC was selectively correlated with MoCA memory subscale scores, which is consistent with evidence that prolonged MMN latency impairs the rapid encoding of auditory signals and delays their transmission to the PHG (Gao et al., 2023), thereby disrupting the function of the left PHG-PCC circuit, which serves as a critical pathway for auditory memory encoding and integration (Jiang et al., 2023; Wei et al., 2025).

The time-window decomposition lends further temporal plausibility to the proposed serial mediation framework. Specifically, MMN latency prolongation indexes a delay at the earliest sensory-detection stage, which appears to precede the late-window alterations observed in both the IFG → STG attention pathway and the PHG–PCC memory pathway. This temporal ordering—early sensory difference → late circuit alteration → cognitive variation—is consistent with the direction of association assumed by the serial mediation model, and thus provides neurophysiological correlational support beyond purely statistical mediation. This cascade from peripheral auditory difference to central connectivity alteration to cognitive change is compatible with the cognitive reserve disorder framework in hearing loss (Gao et al., 2023) and with evidence that both early and late auditory-evoked potentials differ in populations with auditory-related cognitive decline (Wang et al., 2022).

### Left hemisphere-specific functional connectivity

This study demonstrated that patients with USNHL exhibited aberrant functional connectivity in left-hemisphere brain regions, irrespective of the affected ear side. The potential neural mechanisms underlying this hemisphere-specific alteration may be explained by the following two aspects. First, the human brain is hierarchically organized along a core functional gradient spanning from unimodal sensory cortices to transmodal high-order cognitive networks (Margulies et al., 2016). Recent neuroimaging studies of hearing loss have validated that peripheral auditory deafferentation predominantly induces gradient compression and functional dedifferentiation within left-sided transmodal pathways, thereby resulting in left-lateralized connectivity disruptions that are independent of the lateralization of peripheral hearing impairment (Li et al., 2024). Second, in terms of interhemispheric signal integration, binaural auditory inputs are transmitted and integrated via corpus callosum fibers. The left hemisphere acts as a central convergent hub for binaural auditory-cognitive information processing (Elyamany et al., 2024). Unlike the primary auditory cortex that predominantly processes contralateral auditory signals, high-order left association cortices receive bilateral auditory projections and encode the global integrity of systemic auditory input, rather than sensing unilateral ear-specific hearing deficits. This interhemispheric convergence mechanism elucidates the consistent left-sided functional connectivity abnormalities identified in both left and right USNHL patients. Collectively, the cognitive alterations coupled with USNHL arise from the dysfunction of left transmodal auditory-cognitive networks, which is driven by remolded cortical functional gradients and defective interhemispheric signal transmission.

### Limitations

Although this study provides valuable insights into the functional connectivity of the auditory cortex and cognitive function in patients with USNHL, it exhibits the following limitations. First, the generalizability of findings is restricted. To isolate neural alterations driven by USNHL and minimize age-related neurodegeneration confounds, we only enrolled participants aged 18–60 years. As ARHL and associated cognitive decline predominantly affect adults ≥65 years, our conclusions cannot be directly extrapolated to this clinically most relevant elderly population. Future studies including older cohorts are needed. Second, the sample size is relatively small. Larger samples stratified by disease duration, severity, and laterality (unilateral vs. bilateral SNHL) are warranted in future work. Third, cognitive assessment was limited to the MoCA, a global screening scale with insufficient sensitivity for dissecting subcomponents of auditory episodic memory or executive function, and cannot fully dissociate peripheral auditory input degradation from genuine central cognitive decline. This restricts precise mapping between observed connectivity abnormalities (e.g., PHG–PCC memory pathway, IFG–STG frontotemporal pathway) and specific cognitive phenotypes. Future work should supplement domain-specific tests such as the AVLT and Trail Making Test to improve mechanistic interpretation. Fourth, only sLORETA was employed for cortical source reconstruction prior to PGC analysis, without cross-validation against alternative inverse methods (e.g., MNE/dSPM). Although 256-channel high-density EEG reduces spatial dispersion and inter-regional signal leakage, it cannot eliminate inherent systematic discrepancies among ESI algorithms, which may influence connectivity estimates (Pascarella et al., 2023). Fifth, both serial mediation modeling and partial Granger causality only capture statistical predictive relationships in cross-sectional data, which cannot confirm causal mechanisms. PGC reflects statistical temporal predictability rather than definitive neurophysiological causality, and the identified serial pathways represent correlational statistical chains rather than proven causal cascades. Multimodal converging evidence and longitudinal study designs are needed to confirm inter-regional interactions and validate the temporal plausibility of the proposed mediation framework. Finally, the cross-sectional design precludes establishing causal relationships among hearing loss, connectivity abnormalities, and cognitive decline, including the directionality of associations and potential mediating factors (e.g., tinnitus, social isolation). Longitudinal follow-up is required to address these questions.

## Conclusion

In summary, we conclude that in patients with USNHL, the increased influence of the left IFG on the left STG and the reduced influence of the left PHG on the left PCC are related to attention and memory impairments, respectively, and these directional functional connectivity abnormalities may represent neurophysiological correlates associated with cognitive alterations in this population, pending longitudinal validation.

## Data Availability

The original contributions presented in the study are included in the article/Supplementary material, further inquiries can be directed to the corresponding author.

## Glossary

ACC: Anterior Cingulate Cortex
AD: Alzheimer’s Disease
AG: angular gyrus
BA: Brodmann Area
DCM: Dynamic Causal Modeling
DLPFC: Dorsolateral Prefrontal Cortex
DMN: Default Mode Network
EEG: Electroencephalogram
ERP: Event-Related Potential
FDR: False Discovery Rate
IFG: Inferior Frontal Gyrus
MMN: Mismatch Negativity
MoCA: Montreal Cognitive Assessment
mPFC: medial prefrontal cortex
PCC: Posterior Cingulate Cortex
PHG: Parahippocampal Gyrus
PGC: Partial Granger Causality
PTA: pure-tone audiometry
ROI: Region of Interest
RBF: Radial Basis Function
rs-fMRI: Resting-State Functional Magnetic Resonance Imaging
sLORETA: Standardized Low-Resolution Electromagnetic Tomography
SNHL: Sensorineural Hearing Loss
STG: Superior Temporal Gyrus
USNHL: Unilateral Sensorineural Hearing Loss
V1: Primary Visual Cortex
