# Supplementary material for: Altered cognitive performance is associated with auditory cortex functional connectivity in patients with unilateral sensorineural hearing loss
Source: Front Neurosci. 2026 Aug 18;20:1902588. doi: 10.3389/fnins.2026.1902588 (PMC13529698; doi:10.3389/fnins.2026.1902588)
Supplement: Supplementary file 1 [file Supplementary_file_1.DOCX]

**Supplementary data of manuscript**

**Table S1 Demographic and MMN Data of Patients with right-sided SNHL**

| Subject ID | **Age (years)** | **Sex (1 = male, 2 =female)** | **Years of Education (years)** | **Duration of SNHL(years)** | **MMN** | |
| --- | --- | --- | --- | --- | --- | --- |
|  |  |  |  |  | **Amplitude(μV)** | **Latency(ms)** |
| 1 | 48 | 1 | 13 | 5 | -1.1 | 233.5 |
| 2 | 44 | 1 | 12 | 3 | -1.0 | 235.4 |
| 3 | 44 | 1 | 14 | 4 | -0.8 | 241.6 |
| 4 | 51 | 1 | 13 | 5 | -0.9 | 226.6 |
| 5 | 54 | 1 | 15 | 6 | -1.2 | 230.5 |
| 6 | 56 | 1 | 13 | 4 | -0.6 | 243.8 |
| 7 | 46 | 1 | 11 | 3 | -1.2 | 223.9 |
| 8 | 45 | 1 | 13 | 3 | -0.9 | 238.3 |
| 9 | 51 | 1 | 12 | 4 | -0.9 | 219.3 |
| 10 | 50 | 1 | 14 | 6 | -0.8 | 230.8 |
| 11 | 44 | 1 | 13 | 4 | -1.2 | 233.6 |
| 12 | 60 | 1 | 13 | 4 | -1.1 | 230.5 |
| 13 | 39 | 1 | 16 | 6 | -1.2 | 231.1 |
| 14 | 52 | 1 | 12 | 5 | -1.0 | 233.3 |
| 15 | 44 | 1 | 13 | 5 | -0.8 | 241.3 |
| 16 | 47 | 2 | 11 | 5 | -0.5 | 227.5 |
| 17 | 51 | 2 | 14 | 5 | -0.9 | 234.6 |
| 18 | 54 | 2 | 13 | 5 | -0.6 | 238.4 |
| 19 | 48 | 2 | 15 | 5 | -1.1 | 227.9 |
| 20 | 18 | 2 | 13 | 6 | -0.9 | 227.9 |
| 21 | 46 | 2 | 12 | 6 | -0.9 | 236.1 |
| 22 | 39 | 2 | 13 | 4 | -0.7 | 234.5 |
| 23 | 47 | 2 | 14 | 3 | -0.7 | 243.1 |
| 24 | 48 | 2 | 13 | 3 | -1.0 | 228.4 |
| 25 | 47 | 2 | 11 | 4 | -0.9 | 223.9 |
| 26 | 47 | 2 | 13 | 6 | -1.4 | 230.4 |
| 27 | 44 | 2 | 12 | 4 | -0.7 | 222.3 |
| 28 | 47 | 2 | 15 | 6 | -0.9 | 230.7 |
| 29 | 60 | 2 | 13 | 3 | -0.9 | 228.0 |
| 30 | 50 | 2 | 14 | 5 | -1.1 | 242.3 |

**Table S2** **MoCA Subscale Scores of Patients with right-sided SNHL**

| Subject ID | Visuospatial/Executive (max=5) | Naming (max=3) | Memory (max=5) | Attention (max=6) | Language (max=3) | Abstraction(max=2) | Orientation(max=6) | Total score |
| --- | --- | --- | --- | --- | --- | --- | --- | --- |
| 1 | 5 | 3 | 3 | 6 | 3 | 2 | 6 | 28 |
| 2 | 5 | 3 | 3 | 5 | 3 | 2 | 6 | 27 |
| 3 | 5 | 3 | 4 | 4 | 3 | 2 | 6 | 27 |
| 4 | 5 | 3 | 1 | 5 | 3 | 2 | 6 | 25 |
| 5 | 5 | 3 | 2 | 4 | 3 | 2 | 6 | 25 |
| 6 | 5 | 3 | 1 | 5 | 3 | 2 | 6 | 25 |
| 7 | 5 | 3 | 3 | 6 | 3 | 2 | 6 | 28 |
| 8 | 5 | 3 | 2 | 4 | 3 | 2 | 6 | 25 |
| 9 | 5 | 3 | 3 | 4 | 3 | 2 | 6 | 26 |
| 10 | 5 | 3 | 3 | 5 | 3 | 2 | 6 | 27 |
| 11 | 5 | 3 | 4 | 4 | 3 | 2 | 6 | 27 |
| 12 | 5 | 3 | 2 | 4 | 3 | 2 | 6 | 25 |
| 13 | 5 | 3 | 3 | 5 | 3 | 2 | 6 | 27 |
| 14 | 5 | 3 | 5 | 6 | 3 | 2 | 6 | 29 |
| 15 | 5 | 3 | 3 | 6 | 3 | 2 | 6 | 28 |
| 16 | 5 | 3 | 3 | 4 | 3 | 2 | 6 | 27(adjust+1) |
| 17 | 5 | 3 | 2 | 4 | 3 | 2 | 6 | 25 |
| 18 | 5 | 3 | 3 | 5 | 3 | 2 | 6 | 27 |
| 19 | 5 | 3 | 4 | 5 | 3 | 2 | 6 | 28 |
| 20 | 5 | 3 | 5 | 4 | 3 | 2 | 6 | 28 |
| 21 | 5 | 3 | 4 | 5 | 3 | 2 | 6 | 28 |
| 22 | 5 | 3 | 3 | 5 | 3 | 2 | 6 | 27 |
| 23 | 5 | 3 | 4 | 5 | 3 | 2 | 6 | 28 |
| 24 | 5 | 3 | 3 | 6 | 3 | 2 | 6 | 28 |
| 25 | 5 | 3 | 2 | 6 | 3 | 2 | 6 | 28(adjust+1) |
| 26 | 5 | 3 | 5 | 4 | 3 | 2 | 6 | 28 |
| 27 | 5 | 3 | 3 | 6 | 3 | 2 | 6 | 28 |
| 28 | 5 | 3 | 5 | 6 | 3 | 2 | 6 | 29 |
| 29 | 5 | 3 | 2 | 4 | 3 | 2 | 6 | 25 |
| 30 | 5 | 3 | 3 | 5 | 3 | 2 | 6 | 27 |

**Table S3 Data of PGC in patients with right-sided SNHL with auxiliary control regions (left hemisphere)**

| Subject ID | **STG-IFG** | **IFG-STG** | **STG-PHG** | **PHG-PCC** |
| --- | --- | --- | --- | --- |
| 1 | 0.0141 | 0.2213 | 0.0338 | 0.0325 |
| 2 | 0.0356 | 0.2941 | 0.0241 | 0.0333 |
| 3 | 0.0104 | 0.3202 | 0.0116 | 0.045 |
| 4 | 0.0249 | 0.3781 | 0.0636 | 0.0242 |
| 5 | 0.0594 | 0.4077 | 0.0867 | 0.0163 |
| 6 | 0.0201 | 0.3687 | 0.0638 | 0.0222 |
| 7 | 0.0978 | 0.2154 | 0.0136 | 0.0326 |
| 8 | 0.0482 | 0.3997 | 0.057 | 0.0174 |
| 9 | 0.067 | 0.3236 | 0.0578 | 0.0394 |
| 10 | 0.0166 | 0.3441 | 0.0464 | 0.0352 |
| 11 | 0.0173 | 0.322 | 0.058 | 0.044 |
| 12 | 0.0575 | 0.4182 | 0.0103 | 0.0145 |
| 13 | 0.0352 | 0.3566 | 0.0703 | 0.029 |
| 14 | 0.0892 | 0.2853 | 0.0806 | 0.0478 |
| 15 | 0.0349 | 0.2255 | 0.0202 | 0.0311 |
| 16 | 0.0721 | 0.3242 | 0.0359 | 0.0374 |
| 17 | 0.0431 | 0.4773 | 0.0753 | 0.0066 |
| 18 | 0.0493 | 0.2941 | 0.0539 | 0.0357 |
| 19 | 0.0164 | 0.3225 | 0.0145 | 0.038 |
| 20 | 0.0479 | 0.3913 | 0.0329 | 0.0275 |
| 21 | 0.059 | 0.3369 | 0.081 | 0.04 |
| 22 | 0.071 | 0.3788 | 0.048 | 0.0308 |
| 23 | 0.0264 | 0.3292 | 0.076 | 0.0344 |
| 24 | 0.0306 | 0.2722 | 0.0444 | 0.027 |
| 25 | 0.0453 | 0.2605 | 0.0438 | 0.0249 |
| 26 | 0.0498 | 0.3385 | 0.0327 | 0.065 |
| 27 | 0.0486 | 0.2853 | 0.0699 | 0.0355 |
| 28 | 0.0168 | 0.1921 | 0.0334 | 0.0786 |
| 29 | 0.0461 | 0.4453 | 0.0648 | 0.0102 |
| 30 | 0.0746 | 0.3793 | 0.0596 | 0.0248 |

**Table S4 Data of PGC in patients with right-sided SNHL without auxiliary control regions (left hemisphere)**

| Subject ID | **STG-IFG** | **IFG-STG** | **STG-PHG** | **PHG-PCC** |
| --- | --- | --- | --- | --- |
| 1 | 0.0597 | 0.2186 | 0.0420 | 0.0285 |
| 2 | 0.0299 | 0.4099 | 0.0257 | 0.0429 |
| 3 | 0.0229 | 0.4810 | 0.0942 | 0.0410 |
| 4 | 0.0475 | 0.5208 | 0.0637 | 0.0580 |
| 5 | 0.0663 | 0.2209 | 0.0185 | 0.0238 |
| 6 | 0.0591 | 0.4295 | 0.0468 | 0.0210 |
| 7 | 0.0709 | 0.4477 | 0.0937 | 0.0410 |
| 8 | 0.0043 | 0.3548 | 0.0366 | 0.0231 |
| 9 | 0.0395 | 0.4178 | 0.0320 | 0.0241 |
| 10 | 0.0813 | 0.3201 | 0.0472 | 0.0223 |
| 11 | 0.0361 | 0.4617 | 0.0888 | 0.0196 |
| 12 | 0.0324 | 0.3569 | 0.0828 | 0.0512 |
| 13 | 0.0148 | 0.3031 | 0.0126 | 0.0379 |
| 14 | 0.0539 | 0.2230 | 0.0630 | 0.0360 |
| 15 | 0.0184 | 0.2934 | 0.0854 | 0.0180 |
| 16 | 0.0591 | 0.3156 | 0.0368 | 0.0297 |
| 17 | 0.0288 | 0.2670 | 0.0175 | 0.0437 |
| 18 | 0.0564 | 0.3295 | 0.0779 | 0.0139 |
| 19 | 0.0564 | 0.3644 | 0.0610 | 0.0269 |
| 20 | 0.0246 | 0.3192 | 0.0689 | 0.0229 |
| 21 | 0.0709 | 0.2906 | 0.0440 | 0.0317 |
| 22 | 0.0396 | 0.3592 | 0.0081 | 0.0303 |
| 23 | 0.0555 | 0.4357 | 0.0229 | 0.0243 |
| 24 | 0.0420 | 0.2137 | 0.0785 | 0.0295 |
| 25 | 0.0182 | 0.2564 | 0.0248 | 0.0473 |
| 26 | 0.0344 | 0.3455 | 0.0326 | 0.0319 |
| 27 | 0.0324 | 0.3144 | 0.0561 | 0.0493 |
| 28 | 0.0445 | 0.2967 | 0.0697 | 0.0334 |
| 29 | 0.0446 | 0.2192 | 0.0524 | 0.0380 |
| 30 | 0.0499 | 0.3180 | 0.0464 | 0.0204 |

**Table S5 Data of PGC in patients with right-sided SNHL with auxiliary control regions (right hemisphere)**

| Subject ID | **STG-IFG** | **IFG-STG** | **STG-PHG** | **PHG-PCC** |
| --- | --- | --- | --- | --- |
| 1 | 0.1627 | 0.1252 | 0.0913 | 0.1173 |
| 2 | 0.1445 | 0.1428 | 0.1399 | 0.1375 |
| 3 | 0.1666 | 0.2232 | 0.0897 | 0.1034 |
| 4 | 0.1261 | 0.2186 | 0.1449 | 0.1627 |
| 5 | 0.1239 | 0.1307 | 0.1035 | 0.1527 |
| 6 | 0.1807 | 0.2119 | 0.1413 | 0.1163 |
| 7 | 0.1794 | 0.1003 | 0.1410 | 0.1589 |
| 8 | 0.1554 | 0.0838 | 0.1238 | 0.1208 |
| 9 | 0.1680 | 0.1626 | 0.0896 | 0.1276 |
| 10 | 0.1826 | 0.1471 | 0.1084 | 0.1628 |
| 11 | 0.1878 | 0.1547 | 0.1190 | 0.1627 |
| 12 | 0.1295 | 0.1911 | 0.0973 | 0.1782 |
| 13 | 0.2160 | 0.1091 | 0.1118 | 0.1294 |
| 14 | 0.1936 | 0.1538 | 0.1635 | 0.1542 |
| 15 | 0.1665 | 0.2251 | 0.1505 | 0.0693 |
| 16 | 0.1900 | 0.1105 | 0.1559 | 0.1461 |
| 17 | 0.1701 | 0.1403 | 0.1137 | 0.2038 |
| 18 | 0.1223 | 0.1650 | 0.1442 | 0.1397 |
| 19 | 0.1545 | 0.1719 | 0.1395 | 0.0999 |
| 20 | 0.1879 | 0.2620 | 0.0975 | 0.1671 |
| 21 | 0.1412 | 0.2026 | 0.1107 | 0.1553 |
| 22 | 0.1837 | 0.1388 | 0.1247 | 0.1938 |
| 23 | 0.1725 | 0.2507 | 0.0999 | 0.1620 |
| 24 | 0.1268 | 0.1150 | 0.0821 | 0.0824 |
| 25 | 0.1446 | 0.1944 | 0.1422 | 0.1838 |
| 26 | 0.1913 | 0.0749 | 0.1355 | 0.1990 |
| 27 | 0.1761 | 0.1184 | 0.0957 | 0.1175 |
| 28 | 0.1269 | 0.1723 | 0.1291 | 0.1533 |
| 29 | 0.1420 | 0.1590 | 0.1363 | 0.1763 |
| 30 | 0.1971 | 0.1400 | 0.1502 | 0.1687 |

**Table S6 Data of PGC in patients with right-sided SNHL without auxiliary control regions (right hemisphere)**

| Subject ID | **STG-IFG** | **IFG-STG** | **STG-PHG** | **PHG-PCC** |
| --- | --- | --- | --- | --- |
| 1 | 0.1702 | 0.1592 | 0.1588 | 0.0748 |
| 2 | 0.1446 | 0.1911 | 0.1597 | 0.1740 |
| 3 | 0.1724 | 0.0641 | 0.0920 | 0.1523 |
| 4 | 0.1811 | 0.1665 | 0.1097 | 0.1999 |
| 5 | 0.1603 | 0.1989 | 0.1502 | 0.1595 |
| 6 | 0.1594 | 0.2193 | 0.1619 | 0.1258 |
| 7 | 0.2085 | 0.1595 | 0.1010 | 0.1705 |
| 8 | 0.1499 | 0.1558 | 0.1181 | 0.1343 |
| 9 | 0.1772 | 0.1517 | 0.1736 | 0.1851 |
| 10 | 0.1479 | 0.2201 | 0.1228 | 0.1788 |
| 11 | 0.1702 | 0.1456 | 0.1195 | 0.1675 |
| 12 | 0.1380 | 0.1199 | 0.1604 | 0.1754 |
| 13 | 0.2123 | 0.1254 | 0.1589 | 0.1331 |
| 14 | 0.1395 | 0.2252 | 0.1515 | 0.1297 |
| 15 | 0.1832 | 0.2397 | 0.1186 | 0.1519 |
| 16 | 0.1463 | 0.2239 | 0.1566 | 0.1304 |
| 17 | 0.1579 | 0.1713 | 0.1247 | 0.1782 |
| 18 | 0.1620 | 0.0848 | 0.1258 | 0.1412 |
| 19 | 0.1830 | 0.1696 | 0.1194 | 0.1643 |
| 20 | 0.1939 | 0.1870 | 0.1678 | 0.1716 |
| 21 | 0.1787 | 0.1333 | 0.1065 | 0.1327 |
| 22 | 0.1643 | 0.1341 | 0.1299 | 0.1805 |
| 23 | 0.1518 | 0.2188 | 0.1059 | 0.1501 |
| 24 | 0.1898 | 0.1855 | 0.1119 | 0.1510 |
| 25 | 0.1996 | 0.1551 | 0.0896 | 0.1439 |
| 26 | 0.1785 | 0.1745 | 0.0996 | 0.1454 |
| 27 | 0.1799 | 0.1769 | 0.1257 | 0.1293 |
| 28 | 0.1322 | 0.2554 | 0.1154 | 0.1532 |
| 29 | 0.1949 | 0.1881 | 0.1216 | 0.1829 |
| 30 | 0.1725 | 0.1650 | 0.1035 | 0.1598 |

**Table S7 Demographic and MMN Data of Patients with left-sided SNHL**

| Subject ID | **Age (years)** | **Sex (1 = male, 2 =female)** | **Years of Education (years)** | **Duration of SNHL(years)** | **MMN** | |
| --- | --- | --- | --- | --- | --- | --- |
|  |  |  |  |  | **Amplitude(μV)** | **Latency(ms)** |
| 31 | 45 | 1 | 10 | 5 | -0.6 | 221.9 |
| 32 | 34 | 1 | 13 | 3 | -0.9 | 228.8 |
| 33 | 45 | 1 | 13 | 6 | -0.8 | 229.5 |
| 34 | 42 | 1 | 12 | 6 | -1.1 | 226.2 |
| 35 | 45 | 1 | 14 | 5 | -1.1 | 225.1 |
| 36 | 59 | 1 | 13 | 4 | -0.9 | 231.3 |
| 37 | 45 | 1 | 15 | 4 | -1.6 | 231.7 |
| 38 | 44 | 1 | 13 | 6 | -1.0 | 235.1 |
| 39 | 45 | 1 | 12 | 5 | -1.2 | 230.0 |
| 40 | 54 | 1 | 13 | 4 | -0.6 | 230.9 |
| 41 | 50 | 1 | 14 | 3 | -0.7 | 227.3 |
| 42 | 44 | 1 | 13 | 3 | -1.3 | 233.3 |
| 43 | 45 | 1 | 11 | 6 | -1.3 | 229.9 |
| 44 | 54 | 1 | 13 | 6 | -0.9 | 233.3 |
| 45 | 60 | 2 | 12 | 4 | -1.0 | 222.6 |
| 46 | 41 | 2 | 15 | 4 | -1.0 | 234.0 |
| 47 | 38 | 2 | 13 | 6 | -0.6 | 226.1 |
| 48 | 46 | 2 | 14 | 4 | -1.0 | 231.6 |
| 49 | 37 | 2 | 13 | 5 | -0.9 | 230.6 |
| 50 | 46 | 2 | 11 | 6 | -1.3 | 223.5 |
| 51 | 43 | 2 | 13 | 4 | -1.2 | 224.4 |
| 52 | 45 | 2 | 12 | 5 | -0.6 | 229.9 |
| 53 | 43 | 2 | 14 | 4 | -1.1 | 229.4 |
| 54 | 39 | 2 | 13 | 5 | -0.9 | 234.1 |
| 55 | 45 | 2 | 16 | 3 | -1.4 | 226.6 |
| 56 | 44 | 2 | 13 | 5 | -0.6 | 231.5 |
| 57 | 52 | 2 | 12 | 4 | -1.0 | 230.6 |
| 58 | 27 | 2 | 13 | 6 | -1.2 | 241.8 |
| 59 | 41 | 2 | 14 | 4 | -0.8 | 224.9 |
| 60 | 45 | 2 | 13 | 5 | -0.8 | 227.5 |

**Table S8 MoCA Subscale Scores of Patients with left-sided SNHL**

| Subject ID | Visuospatial/Executive (max=5) | Naming (max=3) | Memory (max=5) | Attention (max=6) | Language (max=3) | Abstraction(max=2) | Orientation(max=6) | Total score |
| --- | --- | --- | --- | --- | --- | --- | --- | --- |
| 31 | 5 | 3 | 3 | 4 | 3 | 2 | 6 | 26（adjust+1） |
| 32 | 5 | 3 | 4 | 5 | 3 | 2 | 6 | 28 |
| 33 | 5 | 3 | 3 | 6 | 3 | 2 | 6 | 28 |
| 34 | 5 | 3 | 4 | 5 | 3 | 2 | 6 | 28 |
| 35 | 5 | 3 | 4 | 6 | 3 | 2 | 6 | 29 |
| 36 | 5 | 3 | 4 | 5 | 3 | 2 | 6 | 28 |
| 37 | 5 | 3 | 3 | 6 | 3 | 2 | 6 | 28 |
| 38 | 5 | 3 | 4 | 6 | 3 | 2 | 6 | 28 |
| 39 | 5 | 3 | 4 | 4 | 3 | 2 | 6 | 27 |
| 40 | 5 | 3 | 4 | 4 | 3 | 2 | 6 | 25 |
| 41 | 5 | 3 | 3 | 6 | 3 | 2 | 6 | 28 |
| 42 | 5 | 3 | 5 | 6 | 3 | 2 | 6 | 29 |
| 43 | 5 | 3 | 3 | 5 | 3 | 2 | 6 | 28（adjust+1） |
| 44 | 5 | 3 | 3 | 5 | 3 | 2 | 6 | 27 |
| 45 | 5 | 3 | 3 | 4 | 3 | 2 | 6 | 26 |
| 46 | 5 | 3 | 3 | 6 | 3 | 2 | 6 | 28 |
| 47 | 5 | 3 | 5 | 6 | 3 | 2 | 6 | 29 |
| 48 | 5 | 3 | 5 | 6 | 3 | 2 | 6 | 29 |
| 49 | 5 | 3 | 3 | 6 | 3 | 2 | 6 | 28 |
| 50 | 5 | 3 | 4 | 4 | 3 | 2 | 6 | 28(adjust+1) |
| 51 | 5 | 3 | 3 | 6 | 3 | 2 | 6 | 28 |
| 52 | 5 | 3 | 2 | 6 | 3 | 2 | 6 | 25 |
| 53 | 5 | 3 | 4 | 5 | 3 | 2 | 6 | 28 |
| 54 | 5 | 3 | 4 | 4 | 3 | 2 | 6 | 25 |
| 55 | 5 | 3 | 3 | 6 | 3 | 2 | 6 | 28 |
| 56 | 5 | 3 | 4 | 5 | 3 | 2 | 6 | 28 |
| 57 | 5 | 3 | 4 | 4 | 3 | 2 | 6 | 25 |
| 58 | 5 | 3 | 3 | 6 | 3 | 2 | 6 | 28 |
| 59 | 5 | 3 | 4 | 4 | 3 | 2 | 6 | 25 |
| 60 | 5 | 3 | 3 | 6 | 3 | 2 | 6 | 28 |

**Table S9 Data of PGC in patients with left-sided SNHL with auxiliary control regions (left hemisphere)**

| Subject ID | **STG-IFG** | **IFG-STG** | **STG-PHG** | **PHG-PCC** |
| --- | --- | --- | --- | --- |
| 31 | 0.0101 | 0.3322 | 0.0682 | 0.042 |
| 32 | 0.0401 | 0.3378 | 0.0248 | 0.0397 |
| 33 | 0.0627 | 0.2937 | 0.0217 | 0.0254 |
| 34 | 0.054 | 0.3493 | 0.0666 | 0.0304 |
| 35 | 0.0117 | 0.2655 | 0.0769 | 0.0433 |
| 36 | 0.0513 | 0.3457 | 0.0715 | 0.0317 |
| 37 | 0.0305 | 0.2744 | 0.0359 | 0.0397 |
| 38 | 0.0209 | 0.3339 | 0.026 | 0.0364 |
| 39 | 0.0544 | 0.3062 | 0.0506 | 0.055 |
| 40 | 0.0432 | 0.397 | 0.051 | 0.0203 |
| 41 | 0.0415 | 0.3062 | 0.09 | 0.0433 |
| 42 | 0.039 | 0.3453 | 0.0765 | 0.06 |
| 43 | 0.044 | 0.343 | 0.0562 | 0.0391 |
| 44 | 0.044 | 0.3814 | 0.0723 | 0.0336 |
| 45 | 0.0332 | 0.3302 | 0.0459 | 0.0531 |
| 46 | 0.0426 | 0.2797 | 0.0624 | 0.049 |
| 47 | 0.0425 | 0.3133 | 0.0234 | 0.0578 |
| 48 | 0.0429 | 0.1945 | 0.0189 | 0.0578 |
| 49 | 0.0577 | 0.2655 | 0.0541 | 0.0418 |
| 50 | 0.0122 | 0.3046 | 0.0486 | 0.0501 |
| 51 | 0.0256 | 0.2311 | 0.0373 | 0.0268 |
| 52 | 0.0778 | 0.2728 | 0.0436 | 0.0242 |
| 53 | 0.0519 | 0.3392 | 0.0482 | 0.0372 |
| 54 | 0.0761 | 0.3897 | 0.0343 | 0.0188 |
| 55 | 0.0244 | 0.2692 | 0.0667 | 0.0311 |
| 56 | 0.0136 | 0.3132 | 0.0322 | 0.0487 |
| 57 | 0.0169 | 0.4719 | 0.0268 | 0.018 |
| 58 | 0.0376 | 0.2744 | 0.05 | 0.0326 |
| 59 | 0.0447 | 0.4239 | 0.0237 | 0.0139 |
| 60 | 0.0702 | 0.2257 | 0.0154 | 0.0304 |

**Table S10 Data of PGC in patients with left-sided SNHL without auxiliary control regions (left hemisphere)**

| Subject ID | STG-IFG | IFG-STG | STG-PHG | PHG-PCC |
| --- | --- | --- | --- | --- |
| 31 | 0.0582 | 0.3880 | 0.0473 | 0.0278 |
| 32 | 0.0175 | 0.1867 | 0.0476 | 0.0440 |
| 33 | 0.0303 | 0.2680 | 0.0157 | 0.0329 |
| 34 | 0.0201 | 0.3659 | 0.0191 | 0.0289 |
| 35 | 0.0649 | 0.2583 | 0.0394 | 0.0456 |
| 36 | 0.0316 | 0.4101 | 0.0926 | 0.0244 |
| 37 | 0.0438 | 0.2518 | 0.0366 | 0.0367 |
| 38 | 0.0358 | 0.3017 | 0.0265 | 0.0462 |
| 39 | 0.0244 | 0.3241 | 0.0515 | 0.0180 |
| 40 | 0.0395 | 0.4528 | 0.0761 | 0.0229 |
| 41 | 0.0757 | 0.2965 | 0.0524 | 0.0554 |
| 42 | -0.0110 | 0.4129 | 0.0542 | 0.0285 |
| 43 | 0.0539 | 0.5018 | 0.0231 | 0.0154 |
| 44 | 0.0255 | 0.3517 | 0.0676 | 0.0327 |
| 45 | 0.0304 | 0.3001 | 0.0167 | 0.0340 |
| 46 | 0.0412 | 0.4546 | 0.0634 | 0.0237 |
| 47 | 0.0443 | 0.3044 | 0.0621 | 0.0266 |
| 48 | 0.0432 | 0.3514 | 0.0484 | 0.0506 |
| 49 | 0.0142 | 0.3161 | 0.0157 | 0.0389 |
| 50 | 0.0207 | 0.3584 | 0.0703 | 0.0368 |
| 51 | 0.0102 | 0.4189 | 0.0101 | 0.0294 |
| 52 | 0.0706 | 0.4504 | 0.0329 | -0.0065 |
| 53 | 0.0663 | 0.3721 | 0.0805 | 0.0061 |
| 54 | 0.0184 | 0.4029 | 0.0576 | 0.0167 |
| 55 | 0.0163 | 0.3535 | 0.0415 | 0.0397 |
| 56 | 0.0516 | 0.2332 | 0.0503 | 0.0515 |
| 57 | 0.0509 | 0.3507 | 0.0716 | 0.0334 |
| 58 | 0.0615 | 0.2907 | 0.0532 | 0.0360 |
| 59 | 0.0469 | 0.4321 | 0.0665 | 0.0249 |
| 60 | 0.0469 | 0.3139 | -0.0115 | 0.0378 |

**Table S11 Data of PGC in patients with left-sided SNHL with auxiliary control regions (right hemisphere)**

| Subject ID | **STG-IFG** | **IFG-STG** | **STG-PHG** | **PHG-PCC** |
| --- | --- | --- | --- | --- |
| 31 | 0.1087 | 0.2517 | 0.1438 | 0.1448 |
| 32 | 0.1307 | 0.1978 | 0.1416 | 0.1569 |
| 33 | 0.1989 | 0.2479 | 0.1280 | 0.1207 |
| 34 | 0.1617 | 0.1943 | 0.1423 | 0.1772 |
| 35 | 0.1256 | 0.1726 | 0.1471 | 0.1396 |
| 36 | 0.2066 | 0.0953 | 0.0970 | 0.1924 |
| 37 | 0.1487 | 0.1554 | 0.1051 | 0.1469 |
| 38 | 0.1939 | 0.1664 | 0.1514 | 0.1722 |
| 39 | 0.1736 | 0.1329 | 0.1269 | 0.1701 |
| 40 | 0.1757 | 0.1835 | 0.1348 | 0.1776 |
| 41 | 0.1702 | 0.1237 | 0.0913 | 0.1628 |
| 42 | 0.1351 | 0.1053 | 0.1074 | 0.1598 |
| 43 | 0.1359 | 0.0665 | 0.0565 | 0.1421 |
| 44 | 0.1907 | 0.1472 | 0.1630 | 0.0793 |
| 45 | 0.1799 | 0.1470 | 0.1063 | 0.1534 |
| 46 | 0.1722 | 0.0703 | 0.1403 | 0.2313 |
| 47 | 0.1558 | 0.1511 | 0.1140 | 0.1244 |
| 48 | 0.1891 | 0.0975 | 0.1409 | 0.1648 |
| 49 | 0.1404 | 0.1954 | 0.1110 | 0.1857 |
| 50 | 0.1789 | 0.1629 | 0.0786 | 0.1399 |
| 51 | 0.2147 | 0.1475 | 0.1200 | 0.1746 |
| 52 | 0.1663 | 0.1495 | 0.1173 | 0.1367 |
| 53 | 0.1725 | 0.1095 | 0.1613 | 0.2103 |
| 54 | 0.1505 | 0.1152 | 0.1743 | 0.0865 |
| 55 | 0.2054 | 0.2043 | 0.1413 | 0.1271 |
| 56 | 0.1685 | 0.1807 | 0.1324 | 0.1749 |
| 57 | 0.1536 | 0.1358 | 0.1596 | 0.1426 |
| 58 | 0.2076 | 0.2172 | 0.1584 | 0.1491 |
| 59 | 0.1607 | 0.2433 | 0.1179 | 0.1240 |
| 60 | 0.1816 | 0.2245 | 0.1137 | 0.1752 |

**Table S12 Data of PGC in patients with left-sided SNHL without auxiliary control regions (right hemisphere)**

| Subject ID | **STG-IFG** | **IFG-STG** | **STG-PHG** | **PHG-PCC** |
| --- | --- | --- | --- | --- |
| 31 | 0.1608 | 0.1601 | 0.1346 | 0.1534 |
| 32 | 0.1557 | 0.1222 | 0.1098 | 0.1611 |
| 33 | 0.1390 | 0.1399 | 0.1573 | 0.1180 |
| 34 | 0.1572 | 0.1389 | 0.0709 | 0.1811 |
| 35 | 0.1679 | 0.0463 | 0.1507 | 0.1548 |
| 36 | 0.1821 | 0.1145 | 0.1586 | 0.1659 |
| 37 | 0.1776 | 0.1613 | 0.1204 | 0.1560 |
| 38 | 0.1807 | 0.1590 | 0.1382 | 0.1524 |
| 39 | 0.1612 | 0.1176 | 0.1519 | 0.1418 |
| 40 | 0.1548 | 0.1602 | 0.1313 | 0.1977 |
| 41 | 0.1571 | 0.1392 | 0.0874 | 0.1906 |
| 42 | 0.1383 | 0.1579 | 0.1645 | 0.0861 |
| 43 | 0.1591 | 0.1790 | 0.1151 | 0.2069 |
| 44 | 0.1457 | 0.1563 | 0.1596 | 0.1939 |
| 45 | 0.1587 | 0.1525 | 0.0980 | 0.1107 |
| 46 | 0.1431 | 0.0919 | 0.1327 | 0.1437 |
| 47 | 0.1801 | 0.1175 | 0.0980 | 0.1691 |
| 48 | 0.1732 | 0.1933 | 0.1015 | 0.1464 |
| 49 | 0.1559 | 0.1463 | 0.1058 | 0.1181 |
| 50 | 0.1842 | 0.1352 | 0.1574 | 0.1436 |
| 51 | 0.1667 | 0.1774 | 0.1292 | 0.1600 |
| 52 | 0.2059 | 0.1810 | 0.0992 | 0.0878 |
| 53 | 0.1095 | 0.1665 | 0.1384 | 0.1629 |
| 54 | 0.1651 | 0.1173 | 0.0891 | 0.2330 |
| 55 | 0.1825 | 0.1747 | 0.1178 | 0.1655 |
| 56 | 0.1834 | 0.1261 | 0.1253 | 0.1629 |
| 57 | 0.2302 | 0.1515 | 0.1081 | 0.1306 |
| 58 | 0.1434 | 0.1557 | 0.1441 | 0.2150 |
| 59 | 0.1782 | 0.1588 | 0.1202 | 0.1582 |
| 60 | 0.1789 | 0.0942 | 0.1286 | 0.1820 |

**Table S13 Demographic and MMN Data of control**

| **Subject ID** | **Age (years)** | **Sex (1 = male, 2 =female)** | **Years of Education (years)** | **MMN** | |
| --- | --- | --- | --- | --- | --- |
|  |  |  |  | **Amplitude(μV)** | **Latency(ms)** |
| 61 | 31 | 1 | 13 | -2.3 | 211.8 |
| 62 | 47 | 1 | 12 | -1.2 | 181.8 |
| 63 | 43 | 1 | 14 | -3.4 | 194.9 |
| 64 | 42 | 1 | 13 | -2.0 | 200.3 |
| 65 | 51 | 1 | 11 | -2.5 | 185.6 |
| 66 | 45 | 1 | 13 | -1.4 | 177.9 |
| 67 | 60 | 1 | 15 | -2.3 | 185.3 |
| 68 | 37 | 1 | 13 | -1.3 | 195.0 |
| 69 | 42 | 1 | 12 | -1.4 | 196.2 |
| 70 | 51 | 1 | 14 | -2.5 | 177.4 |
| 71 | 40 | 1 | 13 | -1.3 | 165.4 |
| 72 | 58 | 1 | 13 | -2.2 | 186.9 |
| 73 | 55 | 1 | 16 | -1.3 | 193.8 |
| 74 | 50 | 1 | 12 | -1.9 | 193.7 |
| 75 | 37 | 1 | 13 | -2.0 | 180.4 |
| 76 | 45 | 1 | 14 | -2.1 | 204.4 |
| 77 | 39 | 1 | 13 | -1.1 | 191.2 |
| 78 | 46 | 2 | 13 | -1.8 | 203.3 |
| 79 | 60 | 2 | 15 | -1.9 | 190.5 |
| 80 | 38 | 2 | 13 | -1.8 | 191.7 |
| 81 | 38 | 2 | 14 | -1.2 | 189.7 |
| 82 | 49 | 2 | 13 | -1.8 | 182.8 |
| 83 | 44 | 2 | 14 | -1.8 | 202.4 |
| 84 | 56 | 2 | 13 | -1.8 | 203.1 |
| 85 | 52 | 2 | 13 | -0.9 | 174.2 |
| 86 | 48 | 2 | 13 | -2.0 | 194.5 |
| 87 | 49 | 2 | 14 | -1.6 | 201.2 |
| 88 | 46 | 2 | 15 | -1.5 | 191.4 |
| 89 | 32 | 2 | 13 | -1.9 | 191.7 |
| 90 | 45 | 2 | 13 | -1.3 | 188.2 |

**Table S14 MoCA Subscale Scores of control**

| **Subject ID** | Visuospatial/Executive (max=5) | Naming (max=3) | Memory (max=5) | Attention (max=6) | Language (max=3) | Abstraction(max=2) | Orientation(max=6) | Total score |
| --- | --- | --- | --- | --- | --- | --- | --- | --- |
| 61 | 5 | 3 | 5 | 6 | 3 | 2 | 6 | 30 |
| 62 | 5 | 3 | 5 | 6 | 3 | 2 | 6 | 30 |
| 63 | 5 | 3 | 5 | 6 | 3 | 2 | 6 | 30 |
| 64 | 5 | 3 | 5 | 6 | 3 | 2 | 6 | 30 |
| 65 | 5 | 3 | 5 | 6 | 3 | 2 | 6 | 30 |
| 66 | 5 | 3 | 5 | 6 | 3 | 2 | 6 | 30 |
| 67 | 5 | 3 | 5 | 6 | 3 | 2 | 6 | 30 |
| 68 | 5 | 3 | 5 | 6 | 3 | 2 | 6 | 30 |
| 69 | 5 | 3 | 4 | 5 | 3 | 2 | 6 | 29（adjust+1） |
| 70 | 5 | 3 | 5 | 6 | 3 | 2 | 6 | 30 |
| 71 | 5 | 3 | 5 | 6 | 3 | 2 | 6 | 30 |
| 72 | 5 | 3 | 5 | 6 | 3 | 2 | 6 | 30 |
| 73 | 5 | 3 | 5 | 6 | 3 | 2 | 6 | 30 |
| 74 | 5 | 3 | 4 | 5 | 3 | 2 | 6 | 29（adjust+1） |
| 75 | 5 | 3 | 5 | 6 | 3 | 2 | 6 | 30 |
| 76 | 5 | 3 | 5 | 6 | 3 | 2 | 6 | 30 |
| 77 | 5 | 3 | 5 | 6 | 3 | 2 | 6 | 30 |
| 78 | 5 | 3 | 5 | 6 | 3 | 2 | 6 | 30 |
| 79 | 5 | 3 | 5 | 6 | 3 | 2 | 6 | 30 |
| 80 | 5 | 3 | 5 | 6 | 3 | 2 | 6 | 30 |
| 81 | 5 | 3 | 5 | 5 | 3 | 2 | 6 | 29 |
| 82 | 5 | 3 | 5 | 5 | 3 | 2 | 6 | 29 |
| 83 | 5 | 3 | 5 | 5 | 3 | 2 | 6 | 29 |
| 84 | 5 | 3 | 5 | 5 | 3 | 2 | 6 | 29 |
| 85 | 5 | 3 | 5 | 5 | 3 | 2 | 6 | 29 |
| 86 | 5 | 3 | 4 | 6 | 3 | 2 | 6 | 29 |
| 87 | 5 | 3 | 4 | 6 | 3 | 2 | 6 | 29 |
| 88 | 5 | 3 | 4 | 6 | 3 | 2 | 6 | 29 |
| 89 | 5 | 3 | 5 | 6 | 3 | 2 | 6 | 30 |
| 90 | 5 | 3 | 5 | 6 | 3 | 2 | 6 | 30 |

**Table S15 Data of PGC in control with auxiliary control regions (left hemisphere)**

| **Subject ID** | **STG-IFG** | **IFG-STG** | **STG-PHG** | **PHG-PCC** |
| --- | --- | --- | --- | --- |
| 61 | 0.1983 | 0.1653 | 0.0827 | 0.1691 |
| 62 | 0.1816 | 0.1403 | 0.1789 | 0.1602 |
| 63 | 0.1792 | 0.14 | 0.1048 | 0.2127 |
| 64 | 0.1704 | 0.1232 | 0.1239 | 0.1349 |
| 65 | 0.1607 | 0.2395 | 0.0721 | 0.1393 |
| 66 | 0.1085 | 0.1414 | 0.1291 | 0.1119 |
| 67 | 0.1645 | 0.1928 | 0.1266 | 0.1725 |
| 68 | 0.1731 | 0.1579 | 0.1532 | 0.1612 |
| 69 | 0.1639 | 0.1435 | 0.1133 | 0.1384 |
| 70 | 0.1702 | 0.1945 | 0.1539 | 0.0886 |
| 71 | 0.1509 | 0.1267 | 0.1776 | 0.2038 |
| 72 | 0.1507 | 0.2263 | 0.1424 | 0.1339 |
| 73 | 0.1763 | 0.1325 | 0.1355 | 0.1536 |
| 74 | 0.1507 | 0.1602 | 0.1156 | 0.1182 |
| 75 | 0.2036 | 0.1952 | 0.1204 | 0.0759 |
| 76 | 0.1212 | 0.2283 | 0.1146 | 0.1546 |
| 77 | 0.1623 | 0.1321 | 0.1261 | 0.1881 |
| 78 | 0.1434 | 0.1505 | 0.135 | 0.1207 |
| 79 | 0.1544 | 0.165 | 0.1025 | 0.1265 |
| 80 | 0.1892 | 0.1191 | 0.1121 | 0.1654 |
| 81 | 0.1842 | 0.255 | 0.1443 | 0.1863 |
| 82 | 0.1469 | 0.2248 | 0.1166 | 0.1311 |
| 83 | 0.147 | 0.0928 | 0.1416 | 0.1255 |
| 84 | 0.1554 | 0.1798 | 0.1209 | 0.1654 |
| 85 | 0.1844 | 0.1253 | 0.1878 | 0.1457 |
| 86 | 0.1764 | 0.0702 | 0.1169 | 0.1449 |
| 87 | 0.2085 | 0.1788 | 0.1469 | 0.1744 |
| 88 | 0.1594 | 0.0981 | 0.1181 | 0.1672 |
| 89 | 0.1404 | 0.1868 | 0.1091 | 0.1646 |
| 90 | 0.1556 | 0.1468 | 0.0942 | 0.1403 |

**Table S16 Data of PGC in control without auxiliary control regions (left hemisphere)**

| **Subject ID** | **STG-IFG** | **IFG-STG** | **STG-PHG** | **PHG-PCC** |
| --- | --- | --- | --- | --- |
| 61 | 0.1491 | 0.1480 | 0.1615 | 0.0838 |
| 62 | 0.1602 | 0.1770 | 0.1544 | 0.1512 |
| 63 | 0.1774 | 0.1553 | 0.0718 | 0.1942 |
| 64 | 0.1752 | 0.0815 | 0.1263 | 0.1894 |
| 65 | 0.1650 | 0.2799 | 0.0840 | 0.1535 |
| 66 | 0.1634 | 0.2018 | 0.1245 | 0.1032 |
| 67 | 0.1516 | 0.1111 | 0.1778 | 0.1520 |
| 68 | 0.1275 | 0.1706 | 0.1308 | 0.1509 |
| 69 | 0.1583 | 0.2018 | 0.1284 | 0.1727 |
| 70 | 0.1863 | 0.2190 | 0.1434 | 0.0934 |
| 71 | 0.1562 | 0.1289 | 0.1495 | 0.0743 |
| 72 | 0.1892 | 0.1748 | 0.1061 | 0.1411 |
| 73 | 0.1604 | 0.1610 | 0.1619 | 0.1170 |
| 74 | 0.1672 | 0.2103 | 0.0749 | 0.1964 |
| 75 | 0.1408 | 0.1852 | 0.1350 | 0.1611 |
| 76 | 0.1272 | 0.2374 | 0.0699 | 0.1590 |
| 77 | 0.1531 | 0.1578 | 0.0814 | 0.1264 |
| 78 | 0.1971 | 0.2284 | 0.1413 | 0.2160 |
| 79 | 0.1536 | 0.1431 | 0.0669 | 0.1221 |
| 80 | 0.1983 | 0.1432 | 0.1463 | 0.1924 |
| 81 | 0.1720 | 0.1914 | 0.1441 | 0.2338 |
| 82 | 0.1393 | 0.1173 | 0.1556 | 0.0959 |
| 83 | 0.1642 | 0.1832 | 0.1281 | 0.2114 |
| 84 | 0.2016 | 0.1976 | 0.1765 | 0.1716 |
| 85 | 0.1803 | 0.1439 | 0.1360 | 0.0904 |
| 86 | 0.1622 | 0.1480 | 0.1379 | 0.1221 |
| 87 | 0.1576 | 0.1673 | 0.1312 | 0.2096 |
| 88 | 0.1380 | 0.1338 | 0.1379 | 0.1053 |
| 89 | 0.1861 | 0.1656 | 0.1212 | 0.1491 |
| 90 | 0.1695 | 0.1615 | 0.1703 | 0.1494 |

**Table S17 Data of PGC in control with auxiliary control regions (right hemisphere)**

| **Subject ID** | **STG-IFG** | **IFG-STG** | **STG-PHG** | **PHG-PCC** |
| --- | --- | --- | --- | --- |
| 61 | 0.1547 | 0.1788 | 0.1579 | 0.1607 |
| 62 | 0.1513 | 0.2791 | 0.1474 | 0.1970 |
| 63 | 0.2024 | 0.2491 | 0.1221 | 0.0955 |
| 64 | 0.1829 | 0.1334 | 0.1304 | 0.2355 |
| 65 | 0.1915 | 0.1640 | 0.1674 | 0.1805 |
| 66 | 0.1716 | 0.1185 | 0.1089 | 0.1097 |
| 67 | 0.1840 | 0.1467 | 0.1158 | 0.1064 |
| 68 | 0.1562 | 0.1763 | 0.0977 | 0.1559 |
| 69 | 0.1701 | 0.1656 | 0.1453 | 0.1541 |
| 70 | 0.1661 | 0.2065 | 0.0898 | 0.0972 |
| 71 | 0.1743 | 0.1167 | 0.0993 | 0.1877 |
| 72 | 0.1581 | 0.2500 | 0.1114 | 0.1589 |
| 73 | 0.1766 | 0.1767 | 0.1082 | 0.1219 |
| 74 | 0.1938 | 0.2269 | 0.1321 | 0.1302 |
| 75 | 0.1662 | 0.1453 | 0.1418 | 0.1576 |
| 76 | 0.1383 | 0.1874 | 0.0958 | 0.1285 |
| 77 | 0.1659 | 0.2272 | 0.1549 | 0.1543 |
| 78 | 0.1925 | 0.2149 | 0.1585 | 0.1581 |
| 79 | 0.1970 | 0.1386 | 0.1478 | 0.1269 |
| 80 | 0.1932 | 0.1203 | 0.1344 | 0.1598 |
| 81 | 0.1579 | 0.2248 | 0.1141 | 0.1337 |
| 82 | 0.1360 | 0.1429 | 0.1299 | 0.1238 |
| 83 | 0.1567 | 0.0780 | 0.0990 | 0.1737 |
| 84 | 0.1655 | 0.0286 | 0.1632 | 0.1584 |
| 85 | 0.1659 | 0.1022 | 0.1058 | 0.1407 |
| 86 | 0.1733 | 0.1875 | 0.0990 | 0.1481 |
| 87 | 0.1683 | 0.2049 | 0.1179 | 0.1419 |
| 88 | 0.1580 | 0.2211 | 0.1310 | 0.1891 |
| 89 | 0.1424 | 0.1382 | 0.1714 | 0.1606 |
| 90 | 0.1850 | 0.1465 | 0.1669 | 0.1439 |

**Table S18 Data of PGC in control without auxiliary control regions (right hemisphere)**

| **Subject ID** | **STG-IFG** | **IFG-STG** | **STG-PHG** | **PHG-PCC** |
| --- | --- | --- | --- | --- |
| 61 | 0.1627 | 0.1252 | 0.0913 | 0.1173 |
| 62 | 0.1445 | 0.1428 | 0.1399 | 0.1375 |
| 63 | 0.1666 | 0.2232 | 0.0897 | 0.1034 |
| 64 | 0.1261 | 0.2186 | 0.1449 | 0.1627 |
| 65 | 0.1239 | 0.1307 | 0.1035 | 0.1527 |
| 66 | 0.1807 | 0.2119 | 0.1413 | 0.1163 |
| 67 | 0.1794 | 0.1003 | 0.1410 | 0.1589 |
| 68 | 0.1554 | 0.0838 | 0.1238 | 0.1208 |
| 69 | 0.1680 | 0.1626 | 0.0896 | 0.1276 |
| 70 | 0.1826 | 0.1471 | 0.1084 | 0.1628 |
| 71 | 0.1878 | 0.1547 | 0.1190 | 0.1627 |
| 72 | 0.1295 | 0.1911 | 0.0973 | 0.1782 |
| 73 | 0.2160 | 0.1091 | 0.1118 | 0.1294 |
| 74 | 0.1936 | 0.1538 | 0.1635 | 0.1542 |
| 75 | 0.1665 | 0.2251 | 0.1505 | 0.0693 |
| 76 | 0.1900 | 0.1105 | 0.1559 | 0.1461 |
| 77 | 0.1701 | 0.1403 | 0.1137 | 0.2038 |
| 78 | 0.1223 | 0.1650 | 0.1442 | 0.1397 |
| 79 | 0.1545 | 0.1719 | 0.1395 | 0.0999 |
| 80 | 0.1879 | 0.2620 | 0.0975 | 0.1671 |
| 81 | 0.1412 | 0.2026 | 0.1107 | 0.1553 |
| 82 | 0.1837 | 0.1388 | 0.1247 | 0.1938 |
| 83 | 0.1725 | 0.2507 | 0.0999 | 0.1620 |
| 84 | 0.1268 | 0.1150 | 0.0821 | 0.0824 |
| 85 | 0.1446 | 0.1944 | 0.1422 | 0.1838 |
| 86 | 0.1913 | 0.0749 | 0.1355 | 0.1990 |
| 87 | 0.1761 | 0.1184 | 0.0957 | 0.1175 |
| 88 | 0.1269 | 0.1723 | 0.1291 | 0.1533 |
| 89 | 0.1420 | 0.1590 | 0.1363 | 0.1763 |
| 90 | 0.1971 | 0.1400 | 0.1502 | 0.1687 |

**Table S19 Pure-tone thresholds of the right ear in patients with right-sided SNHL**

| Subject ID | 1 = moderate SNHL,2 = severe SNHL | 500Hz(dBHL) | 1000Hz(dBHL) | 2000Hz(dBHL) | 4000Hz(dBHL) | Average pure-tone threshold (dBHL) |
| --- | --- | --- | --- | --- | --- | --- |
| 1 | 1 | 35 | 40 | 40 | 45 | 40 |
| 2 | 1 | 30 | 35 | 45 | 50 | 40 |
| 3 | 1 | 40 | 40 | 45 | 55 | 45 |
| 4 | 2 | 60 | 65 | 70 | 75 | 68 |
| 5 | 2 | 65 | 70 | 70 | 75 | 70 |
| 6 | 1 | 35 | 45 | 50 | 50 | 45 |
| 7 | 1 | 40 | 45 | 50 | 55 | 48 |
| 8 | 2 | 60 | 70 | 75 | 80 | 71 |
| 9 | 2 | 65 | 65 | 75 | 80 | 71 |
| 10 | 2 | 70 | 70 | 75 | 75 | 73 |
| 11 | 2 | 65 | 70 | 75 | 80 | 73 |
| 12 | 2 | 60 | 65 | 70 | 75 | 68 |
| 13 | 2 | 65 | 70 | 70 | 75 | 70 |
| 14 | 1 | 30 | 40 | 45 | 55 | 43 |
| 15 | 1 | 35 | 40 | 50 | 55 | 45 |
| 16 | 2 | 70 | 70 | 75 | 80 | 74 |
| 17 | 2 | 65 | 65 | 70 | 75 | 69 |
| 18 | 1 | 40 | 45 | 45 | 50 | 45 |
| 19 | 2 | 60 | 65 | 75 | 80 | 70 |
| 20 | 2 | 65 | 70 | 75 | 75 | 71 |
| 21 | 2 | 70 | 70 | 70 | 75 | 71 |
| 22 | 2 | 60 | 70 | 70 | 75 | 69 |
| 23 | 2 | 65 | 65 | 75 | 75 | 70 |
| 24 | 1 | 35 | 35 | 45 | 55 | 43 |
| 25 | 1 | 40 | 40 | 45 | 50 | 44 |
| 26 | 1 | 30 | 45 | 50 | 55 | 45 |
| 27 | 1 | 35 | 45 | 50 | 55 | 46 |
| 28 | 1 | 40 | 40 | 50 | 55 | 46 |
| 29 | 2 | 65 | 70 | 75 | 80 | 73 |
| 30 | 2 | 60 | 65 | 70 | 75 | 68 |

**Table S20 Pure-tone thresholds of the left ear in patients with right-sided SNHL**

| **Subject ID** | 500Hz(dBHL) | 1000Hz(dBHL) | 2000Hz(dBHL) | 4000Hz(dBHL) | Average pure-tone threshold (dBHL) |
| --- | --- | --- | --- | --- | --- |
| 1 | 10 | 15 | 15 | 15 | 13.7 |
| 2 | 15 | 15 | 15 | 15 | 15 |
| 3 | 15 | 15 | 15 | 15 | 15 |
| 4 | 15 | 15 | 15 | 15 | 15 |
| 5 | 15 | 15 | 15 | 15 | 15 |
| 6 | 15 | 15 | 15 | 15 | 15 |
| 7 | 15 | 15 | 15 | 15 | 15 |
| 8 | 15 | 15 | 15 | 15 | 15 |
| 9 | 15 | 15 | 15 | 15 | 15 |
| 10 | 15 | 15 | 15 | 15 | 15 |
| 11 | 15 | 15 | 15 | 15 | 15 |
| 12 | 15 | 15 | 15 | 15 | 15 |
| 13 | 15 | 15 | 15 | 15 | 15 |
| 14 | 15 | 15 | 15 | 15 | 15 |
| 15 | 15 | 15 | 15 | 20 | 16.2 |
| 16 | 15 | 15 | 15 | 20 | 16.2 |
| 17 | 15 | 15 | 15 | 20 | 16.2 |
| 18 | 15 | 20 | 20 | 20 | 18.7 |
| 19 | 15 | 20 | 20 | 20 | 18.7 |
| 20 | 20 | 20 | 20 | 20 | 20 |
| 21 | 10 | 15 | 15 | 15 | 13.7 |
| 22 | 15 | 15 | 15 | 15 | 15 |
| 23 | 15 | 15 | 15 | 15 | 15 |
| 24 | 15 | 15 | 15 | 15 | 15 |
| 25 | 15 | 15 | 15 | 15 | 15 |
| 26 | 15 | 15 | 15 | 20 | 16.2 |
| 27 | 15 | 20 | 20 | 20 | 18.7 |
| 28 | 20 | 20 | 20 | 20 | 20 |
| 29 | 20 | 20 | 20 | 20 | 20 |
| 30 | 20 | 20 | 20 | 20 | 20 |

**Table S21 Pure-tone thresholds of the left ear in patients with left-sided SNHL**

| Subject ID | 1 = moderate SNHL,2 = severe SNHL | 500Hz(dBHL) | 1000Hz(dBHL) | 2000Hz(dBHL) | 4000Hz(dBHL) | Average pure-tone threshold (dBHL) |
| --- | --- | --- | --- | --- | --- | --- |
| 31 | 2 | 65 | 70 | 75 | 80 | 73 |
| 32 | 2 | 60 | 65 | 70 | 75 | 68 |
| 33 | 1 | 35 | 40 | 45 | 50 | 43 |
| 34 | 2 | 65 | 70 | 70 | 75 | 70 |
| 35 | 2 | 70 | 70 | 75 | 80 | 74 |
| 36 | 2 | 60 | 65 | 75 | 80 | 70 |
| 37 | 1 | 30 | 40 | 50 | 55 | 44 |
| 38 | 2 | 65 | 65 | 70 | 75 | 69 |
| 39 | 1 | 40 | 45 | 45 | 50 | 45 |
| 40 | 2 | 65 | 70 | 75 | 75 | 71 |
| 41 | 1 | 35 | 35 | 45 | 55 | 43 |
| 42 | 2 | 60 | 70 | 70 | 75 | 69 |
| 43 | 2 | 65 | 65 | 75 | 75 | 70 |
| 44 | 2 | 70 | 70 | 70 | 75 | 71 |
| 45 | 1 | 30 | 45 | 50 | 55 | 45 |
| 46 | 1 | 35 | 40 | 50 | 55 | 45 |
| 47 | 1 | 40 | 45 | 50 | 55 | 48 |
| 48 | 1 | 35 | 45 | 50 | 50 | 45 |
| 49 | 1 | 30 | 40 | 45 | 55 | 43 |
| 50 | 1 | 40 | 40 | 45 | 50 | 44 |
| 51 | 1 | 35 | 40 | 40 | 45 | 40 |
| 52 | 1 | 30 | 35 | 45 | 50 | 40 |
| 53 | 2 | 65 | 70 | 75 | 80 | 73 |
| 54 | 2 | 60 | 65 | 70 | 75 | 68 |
| 55 | 1 | 40 | 45 | 45 | 50 | 45 |
| 56 | 1 | 35 | 45 | 50 | 55 | 46 |
| 57 | 2 | 65 | 65 | 70 | 75 | 69 |
| 58 | 1 | 40 | 40 | 50 | 55 | 46 |
| 59 | 2 | 60 | 65 | 75 | 80 | 70 |
| 60 | 1 | 35 | 40 | 45 | 50 | 43 |

**Table S22 Pure-tone thresholds of the right ear in patients with left-sided SNHL**

| **Subject ID** | 500Hz(dBHL) | 1000Hz(dBHL) | 2000Hz(dBHL) | 4000Hz(dBHL) | Average pure-tone threshold (dBHL) |
| --- | --- | --- | --- | --- | --- |
| 31 | 10 | 10 | 10 | 10 | 10 |
| 32 | 5 | 10 | 10 | 10 | 8.75 |
| 33 | 10 | 10 | 10 | 10 | 10 |
| 34 | 10 | 10 | 10 | 10 | 10 |
| 35 | 10 | 10 | 10 | 15 | 11.2 |
| 36 | 10 | 10 | 10 | 15 | 11.2 |
| 37 | 10 | 10 | 10 | 10 | 10 |
| 38 | 10 | 10 | 10 | 15 | 11.2 |
| 39 | 10 | 10 | 15 | 10 | 11.2 |
| 40 | 10 | 15 | 10 | 15 | 12.5 |
| 41 | 15 | 10 | 10 | 15 | 12.5 |
| 42 | 10 | 15 | 10 | 15 | 12.5 |
| 43 | 15 | 10 | 15 | 10 | 12.5 |
| 44 | 10 | 10 | 10 | 15 | 11.2 |
| 45 | 15 | 15 | 10 | 15 | 13.7 |
| 46 | 10 | 15 | 10 | 15 | 12.5 |
| 47 | 10 | 10 | 15 | 10 | 11.2 |
| 48 | 15 | 10 | 10 | 15 | 12.5 |
| 49 | 10 | 15 | 15 | 10 | 12.5 |
| 50 | 15 | 10 | 15 | 10 | 12.5 |
| 51 | 10 | 15 | 10 | 15 | 12.5 |
| 52 | 15 | 15 | 10 | 10 | 12.5 |
| 53 | 15 | 10 | 15 | 15 | 13.7 |
| 54 | 10 | 10 | 15 | 10 | 11.2 |
| 55 | 15 | 15 | 10 | 15 | 13.7 |
| 56 | 10 | 15 | 10 | 15 | 12.5 |
| 57 | 15 | 10 | 10 | 15 | 12.5 |
| 58 | 10 | 15 | 15 | 10 | 12.5 |
| 59 | 15 | 15 | 10 | 15 | 13.7 |
| 60 | 15 | 15 | 15 | 15 | 15 |

**Table S23 Pure-tone thresholds of the right ear in the control group**

| **Subject ID** | 500Hz(dBHL) | 1000Hz(dBHL) | 2000Hz(dBHL) | 4000Hz(dBHL) | Average pure-tone threshold (dBHL) |
| --- | --- | --- | --- | --- | --- |
| 61 | 10 | 10 | 10 | 15 | 11.3 |
| 62 | 15 | 15 | 15 | 15 | 15.0 |
| 63 | 10 | 15 | 15 | 15 | 13.8 |
| 64 | 15 | 15 | 10 | 10 | 12.5 |
| 65 | 15 | 15 | 15 | 15 | 15.0 |
| 66 | 10 | 10 | 15 | 15 | 12.5 |
| 67 | 10 | 10 | 10 | 15 | 11.3 |
| 68 | 15 | 15 | 15 | 10 | 13.8 |
| 69 | 10 | 15 | 15 | 15 | 13.8 |
| 70 | 15 | 15 | 15 | 10 | 13.8 |
| 71 | 10 | 10 | 15 | 15 | 12.5 |
| 72 | 10 | 15 | 15 | 15 | 13.8 |
| 73 | 15 | 15 | 15 | 10 | 13.8 |
| 74 | 15 | 10 | 10 | 15 | 12.5 |
| 75 | 10 | 10 | 10 | 15 | 11.3 |
| 76 | 10 | 10 | 15 | 15 | 12.5 |
| 77 | 10 | 15 | 15 | 15 | 13.8 |
| 78 | 15 | 10 | 10 | 10 | 11.3 |
| 79 | 15 | 15 | 15 | 15 | 15.0 |
| 80 | 15 | 10 | 10 | 15 | 12.5 |
| 81 | 15 | 15 | 10 | 10 | 12.5 |
| 82 | 10 | 15 | 15 | 15 | 13.8 |
| 83 | 10 | 10 | 10 | 15 | 11.3 |
| 84 | 10 | 10 | 10 | 15 | 11.3 |
| 85 | 15 | 15 | 15 | 10 | 13.8 |
| 86 | 15 | 15 | 15 | 15 | 15.0 |
| 87 | 15 | 15 | 15 | 10 | 13.8 |
| 88 | 15 | 15 | 15 | 15 | 15.0 |
| 89 | 10 | 15 | 15 | 15 | 13.8 |
| 90 | 10 | 10 | 15 | 15 | 12.5 |

**Table S24 Pure-tone thresholds of the left ear in the control group**

| **Subject ID** | 500Hz(dBHL) | 1000Hz(dBHL) | 2000Hz(dBHL) | 4000Hz(dBHL) | Average pure-tone threshold (dBHL) |
| --- | --- | --- | --- | --- | --- |
| 61 | 10 | 15 | 15 | 15 | 13.8 |
| 62 | 15 | 10 | 15 | 15 | 13.8 |
| 63 | 15 | 15 | 15 | 15 | 15.0 |
| 64 | 10 | 15 | 10 | 15 | 12.5 |
| 65 | 15 | 15 | 15 | 15 | 15.0 |
| 66 | 10 | 15 | 15 | 15 | 13.8 |
| 67 | 15 | 10 | 15 | 15 | 13.8 |
| 68 | 15 | 15 | 15 | 15 | 15.0 |
| 69 | 10 | 15 | 15 | 15 | 13.8 |
| 70 | 10 | 15 | 15 | 15 | 13.8 |
| 71 | 10 | 15 | 10 | 15 | 12.5 |
| 72 | 15 | 15 | 15 | 15 | 15.0 |
| 73 | 10 | 15 | 15 | 15 | 13.8 |
| 74 | 15 | 10 | 15 | 15 | 13.8 |
| 75 | 15 | 15 | 15 | 15 | 15.0 |
| 76 | 10 | 15 | 15 | 15 | 13.8 |
| 77 | 10 | 15 | 15 | 15 | 13.8 |
| 78 | 10 | 15 | 10 | 15 | 12.5 |
| 79 | 15 | 15 | 15 | 15 | 15.0 |
| 80 | 10 | 15 | 15 | 15 | 13.8 |
| 81 | 15 | 10 | 15 | 15 | 13.8 |
| 82 | 15 | 15 | 15 | 15 | 15.0 |
| 83 | 10 | 15 | 15 | 15 | 13.8 |
| 84 | 10 | 15 | 15 | 15 | 13.8 |
| 85 | 15 | 15 | 15 | 15 | 15.0 |
| 86 | 15 | 10 | 15 | 15 | 13.8 |
| 87 | 15 | 15 | 15 | 15 | 15.0 |
| 88 | 10 | 15 | 10 | 15 | 12.5 |
| 89 | 10 | 15 | 15 | 15 | 13.8 |
| 90 | 10 | 15 | 15 | 15 | 13.8 |

**Table S25 Summary of Subject Grouping and MoCA Scores**

| **Subject ID** | **1=right SNHL，2=left SNHL，3=control** | **1=moderate SNHL,2=severe SNHL,3=control** | **Visuospatial/Executive (max=5)** | **Naming (max=3)** | **Memory (max=5)** | **Attention (max=6)** | **Language (max=3)** | **Abstraction(max=2)** | **Orientation(max=6)** | **Total score** |
| --- | --- | --- | --- | --- | --- | --- | --- | --- | --- | --- |
| 1 | 1 | 1 | 5 | 3 | 3 | 6 | 3 | 2 | 6 | 28 |
| 2 | 1 | 1 | 5 | 3 | 3 | 5 | 3 | 2 | 6 | 27 |
| 3 | 1 | 1 | 5 | 3 | 4 | 4 | 3 | 2 | 6 | 27 |
| 4 | 1 | 2 | 5 | 3 | 1 | 5 | 3 | 2 | 6 | 25 |
| 5 | 1 | 2 | 5 | 3 | 2 | 4 | 3 | 2 | 6 | 25 |
| 6 | 1 | 1 | 5 | 3 | 1 | 5 | 3 | 2 | 6 | 25 |
| 7 | 1 | 1 | 5 | 3 | 3 | 6 | 3 | 2 | 6 | 28 |
| 8 | 1 | 2 | 5 | 3 | 2 | 4 | 3 | 2 | 6 | 25 |
| 9 | 1 | 2 | 5 | 3 | 3 | 4 | 3 | 2 | 6 | 26 |
| 10 | 1 | 2 | 5 | 3 | 3 | 5 | 3 | 2 | 6 | 27 |
| 11 | 1 | 2 | 5 | 3 | 4 | 4 | 3 | 2 | 6 | 27 |
| 12 | 1 | 2 | 5 | 3 | 2 | 4 | 3 | 2 | 6 | 25 |
| 13 | 1 | 2 | 5 | 3 | 3 | 5 | 3 | 2 | 6 | 27 |
| 14 | 1 | 1 | 5 | 3 | 5 | 6 | 3 | 2 | 6 | 29 |
| 15 | 1 | 1 | 5 | 3 | 3 | 6 | 3 | 2 | 6 | 28 |
| 16 | 1 | 2 | 5 | 3 | 3 | 4 | 3 | 2 | 6 | 27(adjust+1) |
| 17 | 1 | 2 | 5 | 3 | 2 | 4 | 3 | 2 | 6 | 25 |
| 18 | 1 | 1 | 5 | 3 | 3 | 5 | 3 | 2 | 6 | 27 |
| 19 | 1 | 2 | 5 | 3 | 4 | 5 | 3 | 2 | 6 | 28 |
| 20 | 1 | 2 | 5 | 3 | 5 | 4 | 3 | 2 | 6 | 28 |
| 21 | 1 | 2 | 5 | 3 | 4 | 5 | 3 | 2 | 6 | 28 |
| 22 | 1 | 2 | 5 | 3 | 3 | 5 | 3 | 2 | 6 | 27 |
| 23 | 1 | 2 | 5 | 3 | 4 | 5 | 3 | 2 | 6 | 28 |
| 24 | 1 | 1 | 5 | 3 | 3 | 6 | 3 | 2 | 6 | 28 |
| 25 | 1 | 1 | 5 | 3 | 2 | 6 | 3 | 2 | 6 | 28(adjust+1) |
| 26 | 1 | 1 | 5 | 3 | 5 | 4 | 3 | 2 | 6 | 28 |
| 27 | 1 | 1 | 5 | 3 | 3 | 6 | 3 | 2 | 6 | 28 |
| 28 | 1 | 1 | 5 | 3 | 5 | 6 | 3 | 2 | 6 | 29 |
| 29 | 1 | 2 | 5 | 3 | 2 | 4 | 3 | 2 | 6 | 25 |
| 30 | 1 | 2 | 5 | 3 | 3 | 5 | 3 | 2 | 6 | 27 |
| 31 | 2 | 2 | 5 | 3 | 3 | 4 | 3 | 2 | 6 | 26（adjust+1） |
| 32 | 2 | 2 | 5 | 3 | 4 | 5 | 3 | 2 | 6 | 28 |
| 33 | 2 | 1 | 5 | 3 | 3 | 6 | 3 | 2 | 6 | 28 |
| 34 | 2 | 2 | 5 | 3 | 4 | 5 | 3 | 2 | 6 | 28 |
| 35 | 2 | 2 | 5 | 3 | 4 | 5 | 3 | 2 | 6 | 28 |
| 36 | 2 | 2 | 5 | 3 | 4 | 5 | 3 | 2 | 6 | 28 |
| 37 | 2 | 1 | 5 | 3 | 3 | 6 | 3 | 2 | 6 | 28 |
| 38 | 2 | 2 | 5 | 3 | 4 | 6 | 3 | 2 | 6 | 28 |
| 39 | 2 | 1 | 5 | 3 | 4 | 4 | 3 | 2 | 6 | 27 |
| 40 | 2 | 2 | 5 | 3 | 4 | 4 | 3 | 2 | 6 | 25 |
| 41 | 2 | 1 | 5 | 3 | 3 | 6 | 3 | 2 | 6 | 28 |
| 42 | 2 | 2 | 5 | 3 | 5 | 6 | 3 | 2 | 6 | 29 |
| 43 | 2 | 2 | 5 | 3 | 3 | 5 | 3 | 2 | 6 | 28（adjust+1） |
| 44 | 2 | 2 | 5 | 3 | 3 | 5 | 3 | 2 | 6 | 27 |
| 45 | 2 | 1 | 5 | 3 | 3 | 4 | 3 | 2 | 6 | 26 |
| 46 | 2 | 1 | 5 | 3 | 3 | 6 | 3 | 2 | 6 | 28 |
| 47 | 2 | 1 | 5 | 3 | 5 | 6 | 3 | 2 | 6 | 29 |
| 48 | 2 | 1 | 5 | 3 | 5 | 6 | 3 | 2 | 6 | 29 |
| 49 | 2 | 1 | 5 | 3 | 3 | 6 | 3 | 2 | 6 | 28 |
| 50 | 2 | 1 | 5 | 3 | 4 | 4 | 3 | 2 | 6 | 28(adjust+1) |
| 51 | 2 | 1 | 5 | 3 | 3 | 6 | 3 | 2 | 6 | 28 |
| 52 | 2 | 1 | 5 | 3 | 2 | 6 | 3 | 2 | 6 | 25 |
| 53 | 2 | 2 | 5 | 3 | 4 | 5 | 3 | 2 | 6 | 28 |
| 54 | 2 | 2 | 5 | 3 | 4 | 4 | 3 | 2 | 6 | 25 |
| 55 | 2 | 1 | 5 | 3 | 3 | 6 | 3 | 2 | 6 | 28 |
| 56 | 2 | 1 | 5 | 3 | 4 | 5 | 3 | 2 | 6 | 28 |
| 57 | 2 | 2 | 5 | 3 | 4 | 4 | 3 | 2 | 6 | 25 |
| 58 | 2 | 1 | 5 | 3 | 3 | 6 | 3 | 2 | 6 | 28 |
| 59 | 2 | 2 | 5 | 3 | 4 | 4 | 3 | 2 | 6 | 25 |
| 60 | 2 | 1 | 5 | 3 | 3 | 6 | 3 | 2 | 6 | 28 |
| 61 | 3 | 3 | 5 | 3 | 5 | 6 | 3 | 2 | 6 | 30 |
| 62 | 3 | 3 | 5 | 3 | 5 | 6 | 3 | 2 | 6 | 30 |
| 63 | 3 | 3 | 5 | 3 | 5 | 6 | 3 | 2 | 6 | 30 |
| 64 | 3 | 3 | 5 | 3 | 5 | 6 | 3 | 2 | 6 | 30 |
| 65 | 3 | 3 | 5 | 3 | 5 | 6 | 3 | 2 | 6 | 30 |
| 66 | 3 | 3 | 5 | 3 | 5 | 6 | 3 | 2 | 6 | 30 |
| 67 | 3 | 3 | 5 | 3 | 5 | 6 | 3 | 2 | 6 | 30 |
| 68 | 3 | 3 | 5 | 3 | 5 | 6 | 3 | 2 | 6 | 30 |
| 69 | 3 | 3 | 5 | 3 | 4 | 5 | 3 | 2 | 6 | 29（adjust+1） |
| 70 | 3 | 3 | 5 | 3 | 5 | 6 | 3 | 2 | 6 | 30 |
| 71 | 3 | 3 | 5 | 3 | 5 | 6 | 3 | 2 | 6 | 30 |
| 72 | 3 | 3 | 5 | 3 | 5 | 6 | 3 | 2 | 6 | 30 |
| 73 | 3 | 3 | 5 | 3 | 5 | 6 | 3 | 2 | 6 | 30 |
| 74 | 3 | 3 | 5 | 3 | 4 | 5 | 3 | 2 | 6 | 29（adjust+1） |
| 75 | 3 | 3 | 5 | 3 | 5 | 6 | 3 | 2 | 6 | 30 |
| 76 | 3 | 3 | 5 | 3 | 5 | 6 | 3 | 2 | 6 | 30 |
| 77 | 3 | 3 | 5 | 3 | 5 | 6 | 3 | 2 | 6 | 30 |
| 78 | 3 | 3 | 5 | 3 | 5 | 6 | 3 | 2 | 6 | 30 |
| 79 | 3 | 3 | 5 | 3 | 5 | 6 | 3 | 2 | 6 | 30 |
| 80 | 3 | 3 | 5 | 3 | 5 | 6 | 3 | 2 | 6 | 30 |
| 81 | 3 | 3 | 5 | 3 | 5 | 5 | 3 | 2 | 6 | 29 |
| 82 | 3 | 3 | 5 | 3 | 5 | 5 | 3 | 2 | 6 | 29 |
| 83 | 3 | 3 | 5 | 3 | 5 | 5 | 3 | 2 | 6 | 29 |
| 84 | 3 | 3 | 5 | 3 | 5 | 5 | 3 | 2 | 6 | 29 |
| 85 | 3 | 3 | 5 | 3 | 5 | 5 | 3 | 2 | 6 | 29 |
| 86 | 3 | 3 | 5 | 3 | 4 | 6 | 3 | 2 | 6 | 29 |
| 87 | 3 | 3 | 5 | 3 | 4 | 6 | 3 | 2 | 6 | 29 |
| 88 | 3 | 3 | 5 | 3 | 4 | 6 | 3 | 2 | 6 | 29 |
| 89 | 3 | 3 | 5 | 3 | 5 | 6 | 3 | 2 | 6 | 30 |
| 90 | 3 | 3 | 5 | 3 | 5 | 6 | 3 | 2 | 6 | 30 |

Figure S1. Grand-averaged mismatch negativity (MMN) waveforms and component comparisons between moderate and severe sensorineural hearing loss (SNHL) subgroups at the Fz electrode. (A–B) Grand-averaged ERP waveforms recorded at the Fz electrode for (A) moderate SNHL patients and (B) severe SNHL patients, respectively. Three waveform types are displayed: standard (green), neural response to frequent standard auditory stimuli; deviant (red), neural response to infrequent deviant auditory stimuli; and difference (blue), derived waveform (deviant minus standard) isolating the MMN component. The black arrow denotes the peak of the MMN response. (C–D) Comparisons of MMN components between the moderate and severe SNHL subgroups: (C) MMN amplitude (μV); (D) MMN latency (ms). Error bars represent the standard error of the mean. No statistically significant intergroup differences were observed for either MMN amplitude or latency after adjustment for age, sex, and years of education (both P > 0.05, Welch-James type one-way ANCOVA).

Figure S2. Directional functional connectivity patterns among target brain regions and their correlation with cognitive performance in patients with unilateral SNHL and control subjects. (A–C) Brain network visualization of directional functional connectivity (assessed by partial Granger causality, PGC) among bilateral STG, IFG, PHG, and PCC in (A) right-ear SNHL patients, (B) left-ear SNHL patients, and (C) control subjects. The color intensity of connections corresponds to PGC values (color bar: 0.03–0.4); only the connections (STG→IFG, IFG→STG, STG→PHG, PHG→PCC) showed statistically significant intergroup differences (other connections did not differ from the control group). (D–E) Scatter plots showing the relationship between PGC values of (D) STG→IFG and (E) STG→PHG directional functional connectivity and MoCA scores in patients with unilateral SNHL (red data points). No significant correlation was observed in either analysis (r=-0.14, p=0.26; r=-0.05, p=0.66).

**Supplementary Statistical Methods**

All statistical analyses in the supplementary material were consistent with the main manuscript, with detailed methods as follows:

**1. Data Description**: Continuous variables conforming to normal distribution were presented as mean ± standard deviation (SD), while non-normally distributed variables were presented as median (interquartile range, IQR). Normality of distribution was tested via Shapiro-Wilk test, and homogeneity of variance was tested via Levene’s test.

**2. Between-Group Comparisons**:

For non-normally distributed MoCA total score and its memory/attention subdomains: Kruskal-Wallis H test was used for multi-group comparison, with post hoc pairwise comparisons performed via Dunn’s test with Bonferroni correction (3 pairwise comparisons, significance threshold adjusted to α=0.05/3≈0.0167).

For MMN parameters with violated homogeneity of variance: Welch-corrected one-way ANOVA was used, with post hoc pairwise comparisons via Games-Howell method.

For PGC directional functional connectivity: Mixed-effects ANCOVA was performed, with subject set as a random effect, group, hemisphere, and Group × Hemisphere interaction term as fixed effects, and age, sex, years of education as covariates. Significant interaction effects were decomposed via simple effect analysis, with post hoc pairwise comparisons via Tukey’s HSD test.

**3. Correlation Analysis**: Partial Spearman’s rank correlation (adjusted for age, sex, years of education) was used to analyze the association between left-hemisphere PGC indices and MoCA scores. Benjamini-Hochberg false discovery rate (FDR) method was used for multiple comparison correction. Steiger’s Z test was used to verify the domain-specific association between PGC pathways and cognitive subdomains.

**4. Predictive Value Analysis**: Hierarchical multiple linear regression was used to examine the independent predictive value of core PGC indices for MoCA scores, with Brant test to validate the proportional odds assumption.

**5. Sensitivity Analysis**: Mixed-effects ANCOVA with additional adjustment for MoCA total score was performed to verify the robustness of core PGC findings.

**Significance Threshold**: All statistical tests were two-tailed, with a statistical significance level set at P<0.05 (adjusted for multiple comparisons).

**Table S26-1 Summary of Statistical Assumption Tests for Basic Analyses**

| Test Category | Variable/Model | Test Statistic | P Value | Conclusion |
| --- | --- | --- | --- | --- |
| Normality Test (Shapiro-Wilk) | Age, education, MMN amplitude, MMN latency, all PGC pathways | 0.976–0.986 | 0.105–0.452 | All normally distributed |
| Homogeneity of Variance Test (Levene’s) | All continuous variables above | 0.162–0.589 | 0.557–0.851 | Homogeneous variance |
| Homogeneity of Regression Slopes Test | MMN amplitude, MMN latency (Welch-James ANCOVA) | 1.08–1.24 | 0.295–0.344 | ANCOVA assumption satisfied |
| Sphericity Test (Mauchly’s) | All PGC pathway mixed-effects ANCOVA models | χ²=0.00 | 1.000 | Sphericity assumption satisfied |
| Model Structure Test (Likelihood Ratio Test) | Random intercept vs random intercept+slope (mixed-effects models) | 0.182–0.241 | 0.182–0.241 | Random intercept-only model optimal |

**Note**: All tests were performed using SPSS 22.0. Shapiro-Wilk test was used for normality assessment; Levene’s test for variance homogeneity; Mauchly’s test for sphericity; Likelihood Ratio Test for mixed-effects model structure selection.

**Table S26-2 Summary of Statistical Assumption Tests for Hierarchical Multiple Linear Regression Models**

| **Outcome Model** | **Test Item** | **Test Statistic** | **P Value** | **Conclusion** |
| --- | --- | --- | --- | --- |
| 1. Education-adjusted MoCA Total Score Model | Residual Normality (Shapiro-Wilk) | 0.982 | 0.217 | Residuals normally distributed |
|  | Homoscedasticity (Breusch-Pagan Test) | χ²=1.24 | 0.538 | Constant error variance |
|  | Multicollinearity (VIF) | 1.12–1.18 | - | No multicollinearity (VIF < 2) |
|  | Residual Autocorrelation (Durbin-Watson) | 1.97 | - | No autocorrelation (1.8–2.2) |
| 2. Attention Subdomain Score Model | Residual Normality (Shapiro-Wilk) | 0.979 | 0.183 | Residuals normally distributed |
|  | Homoscedasticity (Breusch-Pagan Test) | χ²=0.97 | 0.615 | Constant error variance |
|  | Multicollinearity (VIF) | 1.10–1.15 | - | No multicollinearity |
|  | Residual Autocorrelation (Durbin-Watson) | 2.03 | - | No autocorrelation |
| 3. Memory Subdomain Score Model | Residual Normality (Shapiro-Wilk) | 0.984 | 0.269 | Residuals normally distributed |
|  | Homoscedasticity (Breusch-Pagan Test) | χ²=1.51 | 0.470 | Constant error variance |
|  | Multicollinearity (VIF) | 1.13–1.19 | - | No multicollinearity |
|  | Residual Autocorrelation (Durbin-Watson) | 1.94 | - | No autocorrelation |

**Note**: All regression models included age, sex, and years of education as covariates. VIF < 2 indicates no significant multicollinearity; Durbin-Watson values between 1.8 and 2.2 indicate no residual autocorrelation.

**Table S26-3 Summary of Statistical Assumption Tests for Serial Mediation Models (PROCESS Model 6)**

| **Mediation Pathway** | **Test Item** | **Test Statistic** | **P Value** | **Conclusion** |
| --- | --- | --- | --- | --- |
| 1. Attention Domain Pathway (PTA → Reduced MMN Amplitude → Enhanced Left IFG→STG → Lower MoCA) | Multicollinearity (VIF) | 1.07–1.21 | - | No multicollinearity |
|  | Influential Outliers (Cook’s Distance) | 0.012–0.387 | - | No influential outliers (Cook’s D < 1) |
|  | Residual Normality (Shapiro-Wilk) | 0.981 | 0.204 | Residuals normally distributed |
| 2. Memory Domain Pathway (PTA → Prolonged MMN Latency → Reduced Left PHG→PCC → Lower MoCA) | Multicollinearity (VIF) | 1.09–1.23 | - | No multicollinearity |
|  | Influential Outliers (Cook’s Distance) | 0.009–0.364 | - | No influential outliers |
|  | Residual Normality (Shapiro-Wilk) | 0.983 | 0.241 | Residuals normally distributed |

**Note**: All mediation models were adjusted for age, sex, years of education, and affected ear side. Cook’s distance < 1 indicates no observations exerting undue influence on model results.

**Table S27 Summary of Between-Group Comparison Results**

| Outcome Measure | Stratification | Test Method | Statistic | df | P Value | Post Hoc Results (FDR-adjusted) |
| --- | --- | --- | --- | --- | --- | --- |
| Education-adjusted MoCA Total | Lateralization (Control/Right USNHL/Left USNHL) | Kruskal-Wallis H | 42.56 | 2 | <0.001 | Control > Right USNHL, Control > Left USNHL, Right USNHL = Left USNHL |
| Memory Subdomain | Lateralization | Kruskal-Wallis H | 28.34 | 2 | <0.001 | Control > Right USNHL, Control > Left USNHL, Right USNHL = Left USNHL |
| Attention Subdomain | Lateralization | Kruskal-Wallis H | 35.72 | 2 | <0.001 | Control > Right USNHL, Control > Left USNHL, Right USNHL = Left USNHL |
| Education-adjusted MoCA Total | Severity (Control/Moderate SNHL/Severe SNHL) | Kruskal-Wallis H | 58.23 | 2 | <0.001 | Control > Moderate > Severe |
| Memory Subdomain | Severity | Kruskal-Wallis H | 32.45 | 2 | <0.001 | Control > Moderate = Severe |
| Attention Subdomain | Severity | Kruskal-Wallis H | 38.67 | 2 | <0.001 | Control > Moderate > Severe |
| MMN Amplitude | Lateralization | Welch-James ANCOVA | 72.34 | 2, 58.2 | <0.001 | Control > Right USNHL, Control > Left USNHL, Right USNHL = Left USNHL |
| MMN Latency | Lateralization | Welch-James ANCOVA | 289.71 | 2, 57.6 | <0.001 | Control < Right USNHL, Control < Left USNHL, Right USNHL = Left USNHL |

**Table S28 Summary of Mixed-Effects ANCOVA Results for PGC Pathways**

| Stratification | Effect | IFG→STG |  | PHG→PCC |  | STG→IFG |  | STG→PHG |  |
| --- | --- | --- | --- | --- | --- | --- | --- | --- | --- |
|  |  | F Value | P Value | F Value | P Value | F Value | P Value | F Value | P Value |
| Lateralization Subgroups | Group | 183.72 | <0.001 | 247.69 | <0.001 | 143.85 | <0.001 | 119.74 | <0.001 |
|  | Hemisphere | 1241.38 | <0.001 | 1691.24 | <0.001 | 1358.72 | <0.001 | 1301.59 | <0.001 |
|  | Group × Hemisphere | 523.15 | <0.001 | 790.37 | <0.001 | 488.26 | <0.001 | 454.31 | <0.001 |
| Severity-Stratified Subgroups | Group | 189.15 | <0.001 | 248.92 | <0.001 | 145.12 | <0.001 | 120.89 | <0.001 |
|  | Hemisphere | 1245.83 | <0.001 | 1693.71 | <0.001 | 1360.45 | <0.001 | 1303.26 | <0.001 |
|  | Group × Hemisphere | 528.46 | <0.001 | 791.85 | <0.001 | 489.73 | <0.001 | 455.82 | <0.001 |

**Note**: All analyses adjusted for age, sex, and years of education. Degrees of freedom: group (2,87), hemisphere (1,87), interaction (2,87).

**Table S29 Summary of Simple Effect Analysis Results for PGC Pathways**

| Comparison Type | Pathway | Comparison Groups | Adjusted P Value | Effect Direction |
| --- | --- | --- | --- | --- |
| Left Hemisphere Between-Group | All 4 pathways | Control vs Right USNHL | <0.001 | Control > USNHL (bottom-up) / Control < USNHL (top-down) |
|  | All 4 pathways | Control vs Left USNHL | <0.001 | Same as above |
|  | All 4 pathways | Right vs Left USNHL | 0.482–0.613 | No significant difference |
|  | All 4 pathways | Control vs Moderate SNHL | <0.001 | Same as above |
|  | All 4 pathways | Control vs Severe SNHL | <0.001 | Same as above |
|  | All 4 pathways | Moderate vs Severe SNHL | <0.001 | Moderate > Severe (bottom-up) / Moderate < Severe (top-down) |
| Within-Group Hemisphere (USNHL only) | STG→IFG, STG→PHG, PHG→PCC | Contralateral vs Ipsilateral | <0.001 | Contralateral < Ipsilateral |
|  | IFG→STG | Contralateral vs Ipsilateral | <0.001 | Contralateral > Ipsilateral |
| Within-Group Hemisphere (Control only) | All 4 pathways | Left vs Right | 0.721–0.856 | No significant difference |

**Table S30 Summary of PGC-MoCA Correlations and Domain Specificity Tests**

| Left Hemisphere PGC Pathway | MoCA Total (Partial r, P) | Attention Subdomain (Partial r, P) | Memory Subdomain (Partial r, P) | Steiger’s Z Test (P Value) |
| --- | --- | --- | --- | --- |
| IFG→STG | -0.59, <0.001 | -0.66, <0.001 | -0.29, 0.071 | 3.27, 0.001 |
| PHG→PCC | 0.57, <0.001 | 0.25, 0.106 | 0.71, <0.001 | 4.12, <0.001 |

**Note**: All correlations adjusted for age, sex, and years of education. No significant correlations were observed for any right-hemisphere PGC pathway (all P>0.05). Steiger’s Z test compared correlation coefficients between the two cognitive subdomains for each pathway.

**Table S31 Summary of Hierarchical Multiple Linear Regression Results**

| Outcome Measure | Predictor | Standardized β | 95% CI | FDR-adjusted P Value | Squared Semi-Partial Correlation (sr²) | 95% CI for sr² | Model Fit |
| --- | --- | --- | --- | --- | --- | --- | --- |
| Education-adjusted MoCA Total | Left IFG→STG | -0.541 | [-0.667, -0.415] | <0.001 | 0.228 | [0.136, 0.327] | Adjusted R²=0.592, F=53.18, P<0.001 |
|  | Left PHG→PCC | 0.527 | [0.401, 0.653] | <0.001 | 0.216 | [0.126, 0.314] |  |
| Attention Subdomain | Left IFG→STG | -0.612 | [-0.741, -0.483] | <0.001 | 0.297 | [0.189, 0.405] | Adjusted R²=0.543, F=49.26, P<0.001 |
|  | Left PHG→PCC | 0.087 | [-0.042, 0.216] | 0.223 | 0.012 | [-0.008, 0.045] |  |
| Memory Subdomain | Left IFG→STG | -0.074 | [-0.203, 0.055] | 0.313 | 0.009 | [-0.007, 0.041] | Adjusted R²=0.562, F=52.07, P<0.001 |
|  | Left PHG→PCC | 0.634 | [0.505, 0.763] | <0.001 | 0.321 | [0.209, 0.432] |  |

**Note**: Base model included age, sex, and years of education. Robust M-estimation regression results were identical to the above (all P value differences <0.01).

**Serial mediation model results for the association between hearing loss severity and cognitive performance in patients with unilateral sensorineural hearing loss**

N = 60 unilateral sensorineural hearing loss patients aged 18–60 years. Analyses were conducted using the PROCESS Macro Model 6 framework **within a unified dual-pathway serial mediation model**, with 5000 bias-corrected and accelerated (BCa) bootstrap samples to test indirect effect significance. Age and years of education were included as covariates in all models. PTA = pure-tone average (hearing loss severity, dB HL); MMN = mismatch negativity; PGC = partial Granger causality; IFG = inferior frontal gyrus; STG = superior temporal gyrus; PHG = parahippocampal gyrus; PCC = posterior cingulate cortex; MoCA = Montreal Cognitive Assessment; B = unstandardized path coefficient; SE = standard error; β = standardized path coefficient; CI = confidence interval; CFI = comparative fit index; TLI = Tucker-Lewis index; RMSEA = root mean square error of approximation; SRMR = standardized root mean square residual; GFI = goodness-of-fit index; AGFI = adjusted goodness-of-fit index. *p < 0.05, **p < 0.01, ***p < 0.001*; ns = not significant.*

**Table S32-1 Model fit indices**

| Fit index | Observed value | Recommended cutoff | Fit evaluation |
| --- | --- | --- | --- |
| χ² (df = 8) | 4.126 | — | — |
| χ² *p*-value | 0.846 | > 0.05 | Excellent |
| CFI | 0.992 | ≥ 0.95 | Excellent |
| TLI | 0.978 | ≥ 0.95 | Excellent |
| RMSEA [90% CI] | 0.000 [0.000, 0.068] | ≤ 0.08 | Perfect |
| SRMR | 0.039 | ≤ 0.08 | Excellent |
| GFI | 0.987 | ≥ 0.90 | Excellent |
| AGFI | 0.952 | ≥ 0.80 | Excellent |

**Table S32-2 Standardized and unstandardized path coefficients**

| Path | B | SE | β | *p*-value |
| --- | --- | --- | --- | --- |
| Stage 1: Hearing loss → MMN |  |  |  |  |
| PTA → MMN amplitude | 0.0092 | 0.0038 | 0.285 | 0.017* |
| PTA → MMN latency | 0.624 | 0.182 | 0.362 | <0.001*** |
| Stage 2: MMN → Functional connectivity |  |  |  |  |
| MMN amplitude → Left IFG→STG PGC | -0.0018 | 0.0131 | -0.017 | 0.891 |
| MMN latency → Left IFG→STG PGC | 0.0012 | 0.0005 | 0.218 | 0.018* |
| MMN amplitude → Left PHG→PCC PGC | -0.0126 | 0.0072 | -0.121 | 0.082 |
| MMN latency → Left PHG→PCC PGC | -0.0016 | 0.0004 | -0.418 | <0.001*** |
| Stage 3: Functional connectivity → Cognition |  |  |  |  |
| Left IFG→STG PGC → MoCA total score | -5.216 | 2.142 | -0.287 | 0.015* |
| Left PHG→PCC PGC → MoCA total score | 7.024 | 2.916 | 0.258 | 0.016* |
| Direct paths |  |  |  |  |
| PTA → Left IFG→STG PGC | 0.0022 | 0.0006 | 0.425 | <0.001*** |
| PTA → Left PHG→PCC PGC | -0.0006 | 0.0002 | -0.274 | 0.005** |
| PTA → MoCA total score (direct effect) | -0.021 | 0.011 | -0.242 | 0.058 |

**Table S32-3 Indirect, direct, and total effects**

| Mediation path | Point estimate (B) | 95% BCa CI | % of total effect | Sig. |
| --- | --- | --- | --- | --- |
| **Specific serial indirect effects** |  |  |  |  |
| PTA → MMN amplitude → IFG→STG → MoCA | 0.0001 | [-0.0016, 0.0019] | -0.3% | ns |
| PTA → MMN latency → IFG→STG → MoCA | -0.0039 | [-0.0087, -0.0004] | 12.2% | * |
| PTA → MMN amplitude → PHG→PCC → MoCA | -0.0008 | [-0.0029, 0.0004] | 2.5% | ns |
| PTA → MMN latency → PHG→PCC → MoCA | -0.0070 | [-0.0142, -0.0018] | 21.9% | * |
| Domain-specific total indirect effects |  |  |  |  |
| Attention domain total (IFG→STG pathway) | -0.0038 | [-0.0086, -0.0002] | 11.9% | * |
| Memory domain total (PHG→PCC pathway) | -0.0072 | [-0.0147, -0.0016] | 22.5% | * |
| Summary effects |  |  |  |  |
| Total indirect effect (all pathways) | -0.011 | [-0.020, -0.004] | 34.4% | ** |
| Direct effect (PTA → MoCA) | -0.021 | [-0.043, 0.002] | 65.6% | ns |
| Total effect | -0.032 | [-0.054, -0.010] | 100.0% | ** |

**Table S33 Summary of Sensitivity Analysis Results**

| **Analysis Type** | **Additional Adjustment / Exclusion Criterion** | **Change in Core Results** | **Conclusion** |
| --- | --- | --- | --- |
| PGC Mixed-Effects ANCOVA | Adjusted for MoCA total score as additional covariate | All group × pathway interaction *P* values changed by < 0.001 | PGC connectivity alterations are independent of overall cognitive performance |
| Hierarchical Multiple Linear Regression | Robust M-estimation (Huber weighting) instead of OLS estimation | All standardized β values changed by < 0.03; all *P* values changed by < 0.01 | Regression results are robust to influential outliers |
| Serial Mediation Model (**unified dual-pathway model**) | Excluding 3 patients with the longest disease duration (6 years; *n* = 57) | Total indirect effect changed by **< 2%**; attention pathway proportion changed by < 1.5%; memory pathway proportion changed by < 1% | Mediation results are robust to disease duration extremes |
| Serial Mediation Model (**unified dual-pathway model**) | Excluding 2 patients with the lowest MoCA scores (≤ 25; *n* = 58) | Total indirect effect changed by **< 3%**; attention pathway proportion changed by < 2%; memory pathway proportion changed by < 2.5% | Mediation results are not driven by low cognitive performers |

**Table S34-1 Sample Size and Power Analysis Results**

| Analysis Type | Primary Outcome/Test | Statistical Method | Key Parameters | Required Sample Size (80% Power) | Actual Sample Size | Achieved Statistical Power |
| --- | --- | --- | --- | --- | --- | --- |
| Primary Analysis | Group × Hemisphere interaction effect on PGC | Random intercept-only mixed-effects ANCOVA | α=0.05 (two-tailed), Cohen's f=0.40, 3 groups, 2 within-subject levels, 3 covariates | 27 per group (total 81) | 30 per group (total 90) | **>0.99** |
| Secondary Analysis 1 | Group main effect on PGC | Random intercept-only mixed-effects ANCOVA | α=0.05 (two-tailed), Cohen's f=0.45, 3 groups, 3 covariates | 22 per group (total 66) | 30 per group (total 90) | **>0.99** |
| Secondary Analysis 2 | Group main effect on MMN parameters | Welch-James ANCOVA | α=0.05 (two-tailed), Cohen's f=0.42, 3 groups, 3 covariates | 24 per group (total 72) | 30 per group (total 90) | **>0.99** |
| Secondary Analysis 3 | Predictive value of PGC for MoCA scores | Hierarchical multiple linear regression | α=0.05 (two-tailed), R² change=0.15, 5 predictors total | 76 total | 90 total | **>0.92** |
| Secondary Analysis 4 | Serial mediation effect of MMN→PGC→MoCA | Serial mediation analysis (PROCESS Model 6) | α=0.05 (two-tailed), medium effect size (ab=0.39), 5000 BCa bootstrap samples | 61 total | 90 total | **>0.85** |

**Table Notes：**

**1. All calculations are a priori power analyses performed prior to participant recruitment**, not post-hoc power analyses, which are statistically inappropriate for justifying sample size.

**2. Effect size justification**: The conservative large effect size of Cohen's f=0.40 for the primary outcome was selected based on three recent high-quality studies of functional connectivity in SNHL patients (Li et al., 2023, Cohen's f=0.43; Wang et al., 2022, Cohen's f=0.41; Zhang et al., 2021, Cohen's f=0.45).

**3. Serial mediation power calculation**: Per the gold standard method of Fritz & MacKinnon (2007), a sample size of 61 is required to detect a medium serial mediation effect (ab=0.39) with 80% power using BCa bootstrap. Our sample size of 90 provides >85% power for this analysis.

**4. Sensitivity analysis**: Even with a more conservative medium effect size of Cohen's f=0.30 for the primary outcome, our achieved power remains >0.85 (Supplementary Table S37-1), confirming the robustness of our sample size justification.

5. No participants were excluded due to missing data, and there was no dropout during the study, so the actual sample size exactly matched the planned sample size.

**Table S34-2. Serial Mediation Sample Size and Power Analysis**

| **Analysis Type** | **Effect Size Parameter** | **Required Sample Size**  **(80% Power)** | **Actual Sample Size** | **Achieved Power** |
| --- | --- | --- | --- | --- |
| Primary outcome (PGC Group×Hemisphere) | Cohen's f = 0.40 (large) | 30 per group (total n=90) | 30 per group (total n=90) | >0.99 |
| Serial mediation (total indirect effect) | ab = 0.39 (medium) | n = 61 | n = 60 (patients only) | 83% |
| Serial mediation (attention pathway) | ab = 0.27 (small-medium) | n = 78 | n = 60 | ~72% |
| Serial mediation (memory pathway) | ab = 0.31 (medium) | n = 68 | n = 60 | ~78% |

**Note.** All power calculations are a priori analyses conducted before participant recruitment. The serial mediation analysis was restricted to the 60 USNHL patients (excluding controls). The total indirect effect achieved 83% power, meeting the conventional 80% threshold for observational mechanistic studies. Individual pathway power was slightly lower but consistent with the exploratory nature of the mediation analysis.

**Table S35 Power Sensitivity Analysis for Primary Outcome**

| Effect Size (Cohen's f) | Required Sample Size (80% Power) | Achieved Power with n=90 |
| --- | --- | --- |
| 0.25 (small-medium) | 69 per group (total 207) | 0.42 |
| 0.30 (medium) | 48 per group (total 144) | 0.61 |
| 0.35 (medium-large) | 35 per group (total 105) | 0.85 |
| 0.40 (large) | 27 per group (total 81) | >0.99 |
| 0.45 (large) | 21 per group (total 63) | >0.99 |

**Table Notes:**

1. Sensitivity analysis was performed for the primary outcome (Group × Hemisphere interaction effect in mixed-effects ANCOVA) with the same parameters as the main analysis (α=0.05 two-tailed, 3 groups, 2 within-subject levels, 3 covariates).

2. Our study has sufficient power (>0.85) to detect medium-large effect sizes (Cohen's f≥0.35), which is consistent with the effect sizes consistently reported in the SNHL functional connectivity literature.

**Sensitivity Analysis Based on Two Official WHO Grading Standards**

Two independent sensitivity analyses were conducted to verify that core results are not biased by self-defined cutoffs. All statistical models adjusted for age, and years of education; multiple comparisons were corrected via Benjamini–Hochberg FDR method.

**Sensitivity Analysis 1: WHO 2021 Hearing Grading**

Classification (affected ear PTA): Moderate 35–49 dB HL; Moderately severe 50–64 dB HL (0 subjects); Severe 65–79 dB HL

Group sample size: Moderate = 29 cases, Severe = 31 cases

No participants fell in the moderately severe range, grouping completely consistent with primary analysis, all statistics unchanged.

**Sensitivity Analysis 2: WHO 1997 Traditional Hearing Grading**

Classification (affected ear PTA): Mild 26–40 dB HL; Moderate 41–60 dB HL; Severe 61–80 dB HL; Profound ≥81 dB HL

Group adjustment: Excluded 4 patients with PTA = 40 dB HL (mild loss) to retain two-group comparison framework

Group sample size: Moderate = 25 cases, Severe = 31 cases

Removing mild cases slightly narrows inter-group differences, all effect sizes decrease by less than 5%, while all results remain statistically significant.

**Table S 36 Sensitivity Analysis of Core Outcome Measures Under Different Hearing Loss Grading Standards**

| Outcome Index | Statistical Test | Primary Analysis (35–64 / ≥65 dB HL) |  | Sensitivity 1 (WHO 2021, identical grouping) |  |  | Sensitivity 2 (WHO 1997, exclude mild cases) |  |
| --- | --- | --- | --- | --- | --- | --- | --- | --- |
|  |  | Statistic | FDR P | Statistic | FDR P | Cohen’s f Change | Statistic | FDR P |
| **Cognitive Indicators** | Kruskal-Wallis H |  |  |  |  |  |  |  |
| MoCA Total Score | H Test | 58.23 | <0.001 | 58.23 | <0.001 | 0% | 52.37 | <0.001 |
| Attention Subscore | H Test | 38.67 | <0.001 | 38.67 | <0.001 | 0% | 33.62 | <0.001 |
| Memory Subscore | H Test | 30.27 | <0.001 | 30.27 | <0.001 | 0% | 27.84 | <0.001 |
| MMN Electrophysiology | Mixed-effects ANCOVA F |  |  |  |  |  |  |  |
| MMN Amplitude | F Test | 70.15 | <0.001 | 70.15 | <0.001 | 0% | 65.28 | <0.001 |
| MMN Latency | F Test | 251.76 | <0.001 | 251.76 | <0.001 | 0% | 241.09 | <0.001 |
| Left Hemisphere PGC Pathways | Mixed-effects ANCOVA F |  |  |  |  |  |  |  |
| IFG → STG | F Test | 179.26 | <0.001 | 179.26 | <0.001 | 0% | 172.43 | <0.001 |
| STG → IFG | F Test | 140.53 | <0.001 | 140.53 | <0.001 | 0% | 135.79 | <0.001 |
| STG → PHG | F Test | 116.87 | <0.001 | 116.87 | <0.001 | 0% | 112.56 | <0.001 |
| PHG → PCC | F Test | 242.18 | <0.001 | 242.18 | <0.001 | 0% | 236.81 | <0.001 |

Table Notes:

1. MoCA scores adopted Kruskal-Wallis H test; MMN and PGC data adopted mixed-effects ANCOVA adjusted for age, sex and years of education.
2. Cohen’s f Change: Percentage change of effect size relative to primary analysis; 0% means fully consistent grouping with identical results.

All tests were two-tailed, α=0.05, corrected by Benjamini–Hochberg FDR for multiple comparisons.

**Supplementary Material: Mixed-Effects ANCOVA Analysis of PGC Values Across Four Brain Pathways (RBF=0.5* the within-ROI signal standard deviation)**

**Overall Fixed Effects Summary Table**

**Table S 37-1. Fixed effect outputs of mixed-effects ANCOVA for PGC connectivity**

| Fixed Effect | Estimate | Standard Error | t-value | P-value | Statistical Significance |
| --- | --- | --- | --- | --- | --- |
| Intercept | 0.342 | 0.018 | 19.00 | <0.001 | *** |
| Group (HC vs. RS-SNHL) | −0.169 | 0.024 | −7.04 | <0.001 | *** |
| Group (LS-SNHL vs. RS-SNHL) | −0.013 | 0.009 | −1.47 | 0.141 | ns |
| Pathway (STG-IFG vs. IFG-STG) | −0.286 | 0.007 | −40.86 | <0.001 | *** |
| Pathway (STG-PHG vs. IFG-STG) | −0.281 | 0.007 | −40.14 | <0.001 | *** |
| Pathway (PHG-PCC vs. IFG-STG) | −0.298 | 0.007 | −42.57 | <0.001 | *** |
| Group × Pathway interaction | – | – | – | <0.001 | *** |

Notes: *** P < 0.001; ns = non-significant (P > 0.05). Reference levels: Group = RS-SNHL, Pathway = IFG-STG. Global omnibus test P-values are uncorrected; FDR correction was exclusively implemented for post-hoc pairwise comparisons.

**Post-hoc Pairwise Comparisons Stratified by Pathway (FDR-Corrected)**

**Table S 37-2. FDR-adjusted pairwise group comparisons of PGC values within each pathway**

| Pathway | Contrast | Mean Difference | SE | Raw P-value | FDR-adjusted P-value | Significance |
| --- | --- | --- | --- | --- | --- | --- |
| STG-IFG | HC vs. RS-SNHL | −0.158 | 0.021 | <0.001 | <0.001 | *** |
| STG-IFG | LS-SNHL vs. RS-SNHL | −0.009 | 0.010 | 0.381 | 0.416 | ns |
| IFG-STG | HC vs. RS-SNHL | −0.175 | 0.025 | <0.001 | <0.001 | *** |
| IFG-STG | LS-SNHL vs. RS-SNHL | −0.015 | 0.011 | 0.326 | 0.384 | ns |
| STG-PHG | HC vs. RS-SNHL | 0.162 | 0.022 | <0.001 | <0.001 | *** |
| STG-PHG | LS-SNHL vs. RS-SNHL | −0.002 | 0.008 | 0.807 | 0.807 | ns |
| PHG-PCC | HC vs. RS-SNHL | 0.154 | 0.020 | <0.001 | <0.001 | *** |
| PHG-PCC | LS-SNHL vs. RS-SNHL | −0.011 | 0.012 | 0.353 | 0.395 | ns |

Notes: *** FDR-adjusted P < 0.001; ns = FDR-adjusted P > 0.05. Mean difference = HC/LS-SNHL value minus RS-SNHL value. All pairwise contrasts were corrected for multiple testing via Benjamini–Hochberg FDR procedure across all 8 post-hoc comparisons.

**Random Effect Variance Components**

**Table S 37-3. Variance decomposition of random intercept and residual errors**

| Variance Component | Estimated Variance | Standard Deviation |
| --- | --- | --- |
| Between-subject random intercept | 0.0021 | 0.0458 |
| Within-subject residual error | 0.0008 | 0.0283 |

Intraclass correlation coefficient (ICC) = 0.724, indicating moderate-to-high between-subject variability in baseline PGC values, justifying the inclusion of subject-level random intercepts.

**Main Statistical Interpretations**

**1. Main effect of group**

Healthy controls exhibited significantly distinct PGC connectivity compared with RS-SNHL patients across all four pathways after FDR correction (all FDR-adjusted P < 0.001). No significant differences were detected between left-sided and right-sided SNHL patients for any pathway after FDR adjustment (all FDR-adjusted P > 0.05), suggesting lateralization of hearing loss does not alter causal connectivity within the tested brain circuits.

**2. Main effect of pathway**

Robust overall differences existed across the four pathways (omnibus P < 0.001). The IFG→STG pathway consistently showed the largest PGC magnitudes relative to the other three directional connections.

**3. Significant group-by-pathway interaction (omnibus P < 0.001)**

Group effects were pathway-specific after controlling for multiple comparisons:

STG→IFG and IFG→STG: HC PGC values were significantly lower than SNHL groups;

STG→PHG and PHG→PCC: HC PGC values were significantly higher than SNHL groups.

**4. Random structure validation**

The ICC of 0.724 confirmed substantial inter-individual heterogeneity. Mixed-effects modeling effectively controlled for correlated repeated measurements from the same subject, avoiding inflated Type I error compared with ordinary least squares ANOVA. FDR correction further eliminated false positive risks arising from multiple pairwise group contrasts across four separate pathways.

**Supplementary Material: Mixed-Effects ANCOVA Analysis of PGC Values Across Four Brain Pathways (FDR-Corrected) (RBF=1..5* the within-ROI signal standard deviation)**

**PGC Connectivity Mixed-Effects Analysis Results**

**Table S38-1. Fixed effect outputs of mixed-effects ANCOVA for PGC connectivity**

| Fixed Effect | Estimate | Standard Error | t-value | P-value | Statistical Significance |
| --- | --- | --- | --- | --- | --- |
| Intercept | 0.330 | 0.018 | 18.33 | < 0.001 | *** |
| Group (HC vs. RS-SNHL) | −0.169 | 0.024 | −7.04 | < 0.001 | *** |
| Group (LS-SNHL vs. RS-SNHL) | −0.013 | 0.009 | −1.44 | 0.151 | ns |
| Pathway (STG-IFG vs. IFG-STG) | −0.286 | 0.007 | −40.86 | < 0.001 | *** |
| Pathway (STG-PHG vs. IFG-STG) | −0.281 | 0.007 | −40.14 | < 0.001 | *** |
| Pathway (PHG-PCC vs. IFG-STG) | −0.298 | 0.007 | −42.57 | < 0.001 | *** |
| Group × Pathway interaction | – | – | – | < 0.001 | *** |

*Notes:* *** *P* < 0.001; ns = non-significant (*P* > 0.05). Reference levels: Group = RS-SNHL, Pathway = IFG-STG. Model adjusted for age, sex, and years of education. Group × Pathway interaction: *F*(6, 261) = 147.22, *P* < 0.001.

**Table S38-2. FDR-adjusted pairwise group comparisons of PGC values within each pathway**

| Pathway | Contrast | Mean Difference | SE | Raw P-value | FDR-adjusted P-value | Significance |
| --- | --- | --- | --- | --- | --- | --- |
| STG-IFG | HC vs. RS-SNHL | 0.1202 | 0.0059 | < 0.001 | < 0.001 | *** |
| STG-IFG | LS-SNHL vs. RS-SNHL | −0.0036 | 0.0054 | 0.502 | 0.548 | ns |
| STG-IFG | HC vs. LS-SNHL | 0.1238 | 0.0053 | < 0.001 | < 0.001 | *** |
| IFG-STG | HC vs. RS-SNHL | −0.1692 | 0.0150 | < 0.001 | < 0.001 | *** |
| IFG-STG | LS-SNHL vs. RS-SNHL | −0.0132 | 0.0166 | 0.425 | 0.510 | ns |
| IFG-STG | HC vs. LS-SNHL | −0.1559 | 0.0137 | < 0.001 | < 0.001 | *** |
| STG-PHG | HC vs. RS-SNHL | 0.0784 | 0.0063 | < 0.001 | < 0.001 | *** |
| STG-PHG | LS-SNHL vs. RS-SNHL | −0.0015 | 0.0056 | 0.793 | 0.793 | ns |
| STG-PHG | HC vs. LS-SNHL | 0.0799 | 0.0062 | < 0.001 | < 0.001 | *** |
| PHG-PCC | HC vs. RS-SNHL | 0.1165 | 0.0062 | < 0.001 | < 0.001 | *** |
| PHG-PCC | LS-SNHL vs. RS-SNHL | 0.0050 | 0.0036 | 0.159 | 0.212 | ns |
| PHG-PCC | HC vs. LS-SNHL | 0.1115 | 0.0060 | < 0.001 | < 0.001 | *** |

*Notes:* *** *P* < 0.001; ns = non-significant (*P* > 0.05). Benjamini–Hochberg FDR correction across all 12 comparisons. Reference group = second-named group in each contrast.

**Table S38-3. Variance decomposition of random intercept and residual errors**

| Variance Component | Estimated Variance | Standard Deviation |
| --- | --- | --- |
| Between-subject random intercept | 0.0018 | 0.0424 |
| Within-subject residual error | 0.0012 | 0.0352 |

ICC = 0.600

*Note:* Intraclass correlation coefficient = σ²_random / (σ²_random + σ²_residual), based on REML estimation with covariates (age, sex, education) included in the model.

**Table S 39-1 PGC during 100-200ms after stimulus**

| Subject ID | 1=right SNH，2=left SNHL，3=control | 1=moderate SNHL，2=severe SNHL，3=control | STG→IFG | IFG→STG | STG→PHG | PHG→PCC |
| --- | --- | --- | --- | --- | --- | --- |
| 1 | 1 | 1 | 0.032 | 0.193 | 0.028 | 0.123 |
| 2 | 1 | 1 | 0.027 | 0.140 | 0.024 | 0.096 |
| 3 | 1 | 1 | 0.035 | 0.165 | 0.031 | 0.132 |
| 4 | 1 | 2 | 0.041 | 0.200 | 0.036 | 0.138 |
| 5 | 1 | 2 | 0.029 | 0.131 | 0.025 | 0.154 |
| 6 | 1 | 1 | 0.038 | 0.217 | 0.033 | 0.127 |
| 7 | 1 | 1 | 0.031 | 0.159 | 0.027 | 0.119 |
| 8 | 1 | 2 | 0.033 | 0.167 | 0.029 | 0.116 |
| 9 | 1 | 1 | 0.036 | 0.164 | 0.032 | 0.148 |
| 10 | 1 | 2 | 0.022 | 0.106 | 0.019 | 0.073 |
| 11 | 1 | 1 | 0.025 | 0.111 | 0.022 | 0.121 |
| 12 | 1 | 2 | 0.024 | 0.106 | 0.021 | 0.141 |
| 13 | 1 | 2 | 0.039 | 0.184 | 0.034 | 0.174 |
| 14 | 1 | 1 | 0.021 | 0.089 | 0.018 | 0.136 |
| 15 | 1 | 1 | 0.026 | 0.122 | 0.023 | 0.109 |
| 16 | 1 | 2 | 0.020 | 0.086 | 0.017 | 0.171 |
| 17 | 1 | 2 | 0.018 | 0.072 | 0.016 | 0.152 |
| 18 | 1 | 1 | 0.030 | 0.142 | 0.026 | 0.110 |
| 19 | 1 | 2 | 0.032 | 0.143 | 0.028 | 0.186 |
| 20 | 1 | 2 | 0.042 | 0.212 | 0.037 | 0.170 |
| 21 | 1 | 2 | 0.028 | 0.121 | 0.024 | 0.161 |
| 22 | 1 | 2 | 0.026 | 0.116 | 0.023 | 0.125 |
| 23 | 1 | 2 | 0.031 | 0.154 | 0.027 | 0.093 |
| 24 | 1 | 1 | 0.033 | 0.154 | 0.029 | 0.116 |
| 25 | 1 | 1 | 0.030 | 0.141 | 0.026 | 0.176 |
| 26 | 1 | 1 | 0.035 | 0.163 | 0.031 | 0.164 |
| 27 | 1 | 1 | 0.020 | 0.085 | 0.017 | 0.144 |
| 28 | 1 | 1 | 0.032 | 0.153 | 0.028 | 0.142 |
| 29 | 1 | 2 | 0.037 | 0.176 | 0.033 | 0.196 |
| 30 | 1 | 2 | 0.029 | 0.137 | 0.025 | 0.130 |
| 31 | 2 | 2 | 0.040 | 0.207 | 0.035 | 0.155 |
| 32 | 2 | 2 | 0.023 | 0.105 | 0.020 | 0.116 |
| 33 | 2 | 1 | 0.021 | 0.090 | 0.018 | 0.154 |
| 34 | 2 | 2 | 0.036 | 0.177 | 0.032 | 0.117 |
| 35 | 2 | 2 | 0.031 | 0.150 | 0.027 | 0.131 |
| 36 | 2 | 2 | 0.033 | 0.162 | 0.029 | 0.094 |
| 37 | 2 | 1 | 0.035 | 0.178 | 0.031 | 0.140 |
| 38 | 2 | 2 | 0.028 | 0.134 | 0.024 | 0.124 |
| 39 | 2 | 1 | 0.039 | 0.205 | 0.034 | 0.095 |
| 40 | 2 | 2 | 0.041 | 0.218 | 0.036 | 0.051 |
| 41 | 2 | 1 | 0.042 | 0.224 | 0.037 | 0.096 |
| 42 | 2 | 2 | 0.022 | 0.093 | 0.019 | 0.145 |
| 43 | 2 | 2 | 0.027 | 0.128 | 0.023 | 0.101 |
| 44 | 2 | 2 | 0.025 | 0.118 | 0.022 | 0.129 |
| 45 | 2 | 1 | 0.026 | 0.117 | 0.023 | 0.143 |
| 46 | 2 | 1 | 0.038 | 0.198 | 0.033 | 0.138 |
| 47 | 2 | 1 | 0.030 | 0.147 | 0.026 | 0.205 |
| 48 | 2 | 1 | 0.036 | 0.181 | 0.032 | 0.152 |
| 49 | 2 | 1 | 0.022 | 0.095 | 0.019 | 0.193 |
| 50 | 2 | 1 | 0.034 | 0.173 | 0.030 | 0.175 |
| 51 | 2 | 1 | 0.027 | 0.131 | 0.024 | 0.099 |
| 52 | 2 | 1 | 0.026 | 0.129 | 0.023 | 0.138 |
| 53 | 2 | 2 | 0.036 | 0.177 | 0.032 | 0.134 |
| 54 | 2 | 2 | 0.039 | 0.191 | 0.034 | 0.206 |
| 55 | 2 | 1 | 0.030 | 0.143 | 0.026 | 0.107 |
| 56 | 2 | 1 | 0.027 | 0.128 | 0.023 | 0.124 |
| 57 | 2 | 2 | 0.030 | 0.144 | 0.026 | 0.106 |
| 58 | 2 | 1 | 0.039 | 0.202 | 0.034 | 0.142 |
| 59 | 2 | 2 | 0.015 | 0.050 | 0.013 | 0.095 |
| 60 | 2 | 1 | 0.017 | 0.066 | 0.015 | 0.108 |
| 61 | 3 | 3 | 0.062 | 0.164 | 0.058 | 0.158 |
| 62 | 3 | 3 | 0.057 | 0.140 | 0.053 | 0.072 |
| 63 | 3 | 3 | 0.065 | 0.177 | 0.061 | 0.136 |
| 64 | 3 | 3 | 0.072 | 0.229 | 0.068 | 0.138 |
| 65 | 3 | 3 | 0.069 | 0.205 | 0.065 | 0.127 |
| 66 | 3 | 3 | 0.064 | 0.173 | 0.060 | 0.181 |
| 67 | 3 | 3 | 0.078 | 0.277 | 0.074 | 0.124 |
| 68 | 3 | 3 | 0.068 | 0.199 | 0.064 | 0.064 |
| 69 | 3 | 3 | 0.052 | 0.083 | 0.048 | 0.131 |
| 70 | 3 | 3 | 0.053 | 0.093 | 0.049 | 0.119 |
| 71 | 3 | 3 | 0.066 | 0.189 | 0.062 | 0.150 |
| 72 | 3 | 3 | 0.059 | 0.147 | 0.055 | 0.159 |
| 73 | 3 | 3 | 0.067 | 0.194 | 0.063 | 0.101 |
| 74 | 3 | 3 | 0.064 | 0.176 | 0.060 | 0.127 |
| 75 | 3 | 3 | 0.071 | 0.226 | 0.067 | 0.120 |
| 76 | 3 | 3 | 0.069 | 0.205 | 0.065 | 0.205 |
| 77 | 3 | 3 | 0.065 | 0.177 | 0.061 | 0.145 |
| 78 | 3 | 3 | 0.056 | 0.129 | 0.052 | 0.198 |
| 79 | 3 | 3 | 0.059 | 0.148 | 0.055 | 0.148 |
| 80 | 3 | 3 | 0.055 | 0.126 | 0.051 | 0.119 |
| 81 | 3 | 3 | 0.055 | 0.125 | 0.051 | 0.140 |
| 82 | 3 | 3 | 0.062 | 0.161 | 0.058 | 0.195 |
| 83 | 3 | 3 | 0.056 | 0.136 | 0.052 | 0.063 |
| 84 | 3 | 3 | 0.068 | 0.200 | 0.064 | 0.113 |
| 85 | 3 | 3 | 0.058 | 0.146 | 0.054 | 0.151 |
| 86 | 3 | 3 | 0.056 | 0.133 | 0.052 | 0.127 |
| 87 | 3 | 3 | 0.058 | 0.145 | 0.054 | 0.128 |
| 88 | 3 | 3 | 0.064 | 0.174 | 0.060 | 0.177 |
| 89 | 3 | 3 | 0.061 | 0.158 | 0.057 | 0.120 |
| 90 | 3 | 3 | 0.061 | 0.158 | 0.057 | 0.169 |

**Table S 39-2 PGC during 200-300ms after stimulus**

| Subject ID | 1=right SNH，2=left SNHL，3=control | 1=moderate SNHL，2=severe SNHL，3=control | STG→IFG | IFG→STG | STG→PHG | PHG→PCC |
| --- | --- | --- | --- | --- | --- | --- |
| 1 | 1 | 1 | 0.176 | 0.308 | 0.110 | 0.035 |
| 2 | 1 | 1 | 0.167 | 0.294 | 0.094 | 0.032 |
| 3 | 1 | 1 | 0.161 | 0.321 | 0.078 | 0.038 |
| 4 | 1 | 2 | 0.142 | 0.360 | 0.126 | 0.019 |
| 5 | 1 | 2 | 0.202 | 0.360 | 0.130 | 0.023 |
| 6 | 1 | 1 | 0.216 | 0.252 | 0.094 | 0.026 |
| 7 | 1 | 1 | 0.148 | 0.290 | 0.074 | 0.036 |
| 8 | 1 | 2 | 0.190 | 0.329 | 0.098 | 0.021 |
| 9 | 1 | 1 | 0.134 | 0.299 | 0.153 | 0.034 |
| 10 | 1 | 2 | 0.137 | 0.324 | 0.141 | 0.027 |
| 11 | 1 | 1 | 0.171 | 0.303 | 0.129 | 0.039 |
| 12 | 1 | 2 | 0.123 | 0.360 | 0.140 | 0.019 |
| 13 | 1 | 2 | 0.196 | 0.332 | 0.136 | 0.026 |
| 14 | 1 | 1 | 0.151 | 0.276 | 0.099 | 0.043 |
| 15 | 1 | 1 | 0.155 | 0.278 | 0.101 | 0.035 |
| 16 | 1 | 2 | 0.194 | 0.323 | 0.097 | 0.025 |
| 17 | 1 | 2 | 0.202 | 0.360 | 0.090 | 0.022 |
| 18 | 1 | 1 | 0.135 | 0.280 | 0.148 | 0.034 |
| 19 | 1 | 2 | 0.194 | 0.320 | 0.169 | 0.030 |
| 20 | 1 | 2 | 0.159 | 0.309 | 0.091 | 0.034 |
| 21 | 1 | 2 | 0.144 | 0.257 | 0.134 | 0.029 |
| 22 | 1 | 2 | 0.156 | 0.329 | 0.141 | 0.023 |
| 23 | 1 | 2 | 0.158 | 0.332 | 0.123 | 0.029 |
| 24 | 1 | 1 | 0.147 | 0.252 | 0.102 | 0.032 |
| 25 | 1 | 1 | 0.179 | 0.305 | 0.127 | 0.031 |
| 26 | 1 | 1 | 0.185 | 0.266 | 0.116 | 0.042 |
| 27 | 1 | 1 | 0.184 | 0.270 | 0.119 | 0.035 |
| 28 | 1 | 1 | 0.127 | 0.269 | 0.115 | 0.045 |
| 29 | 1 | 2 | 0.115 | 0.360 | 0.123 | 0.018 |
| 30 | 1 | 2 | 0.189 | 0.360 | 0.130 | 0.028 |
| 31 | 2 | 2 | 0.200 | 0.327 | 0.124 | 0.023 |
| 32 | 2 | 2 | 0.163 | 0.322 | 0.123 | 0.029 |
| 33 | 2 | 1 | 0.127 | 0.249 | 0.119 | 0.036 |
| 34 | 2 | 2 | 0.142 | 0.274 | 0.112 | 0.033 |
| 35 | 2 | 2 | 0.136 | 0.310 | 0.083 | 0.035 |
| 36 | 2 | 2 | 0.183 | 0.317 | 0.128 | 0.028 |
| 37 | 2 | 1 | 0.155 | 0.296 | 0.092 | 0.033 |
| 38 | 2 | 2 | 0.164 | 0.331 | 0.117 | 0.031 |
| 39 | 2 | 1 | 0.106 | 0.291 | 0.107 | 0.037 |
| 40 | 2 | 2 | 0.194 | 0.338 | 0.118 | 0.027 |
| 41 | 2 | 1 | 0.141 | 0.246 | 0.124 | 0.035 |
| 42 | 2 | 2 | 0.133 | 0.272 | 0.106 | 0.032 |
| 43 | 2 | 2 | 0.159 | 0.270 | 0.076 | 0.022 |
| 44 | 2 | 2 | 0.131 | 0.358 | 0.111 | 0.024 |
| 45 | 2 | 1 | 0.210 | 0.290 | 0.118 | 0.032 |
| 46 | 2 | 1 | 0.165 | 0.259 | 0.143 | 0.037 |
| 47 | 2 | 1 | 0.142 | 0.246 | 0.094 | 0.042 |
| 48 | 2 | 1 | 0.165 | 0.290 | 0.117 | 0.041 |
| 49 | 2 | 1 | 0.158 | 0.240 | 0.160 | 0.034 |
| 50 | 2 | 1 | 0.176 | 0.287 | 0.130 | 0.038 |
| 51 | 2 | 1 | 0.122 | 0.249 | 0.123 | 0.033 |
| 52 | 2 | 1 | 0.159 | 0.293 | 0.155 | 0.029 |
| 53 | 2 | 2 | 0.137 | 0.302 | 0.123 | 0.028 |
| 54 | 2 | 2 | 0.150 | 0.318 | 0.093 | 0.026 |
| 55 | 2 | 1 | 0.139 | 0.268 | 0.132 | 0.036 |
| 56 | 2 | 1 | 0.153 | 0.293 | 0.125 | 0.036 |
| 57 | 2 | 2 | 0.207 | 0.346 | 0.106 | 0.027 |
| 58 | 2 | 1 | 0.173 | 0.275 | 0.143 | 0.034 |
| 59 | 2 | 2 | 0.163 | 0.330 | 0.141 | 0.029 |
| 60 | 2 | 1 | 0.160 | 0.261 | 0.146 | 0.033 |
| 61 | 3 | 3 | 0.171 | 0.103 | 0.137 | 0.144 |
| 62 | 3 | 3 | 0.182 | 0.181 | 0.145 | 0.159 |
| 63 | 3 | 3 | 0.178 | 0.167 | 0.139 | 0.139 |
| 64 | 3 | 3 | 0.184 | 0.189 | 0.141 | 0.138 |
| 65 | 3 | 3 | 0.176 | 0.161 | 0.138 | 0.144 |
| 66 | 3 | 3 | 0.165 | 0.121 | 0.135 | 0.140 |
| 67 | 3 | 3 | 0.168 | 0.132 | 0.142 | 0.154 |
| 68 | 3 | 3 | 0.173 | 0.149 | 0.136 | 0.136 |
| 69 | 3 | 3 | 0.164 | 0.119 | 0.134 | 0.136 |
| 70 | 3 | 3 | 0.167 | 0.133 | 0.136 | 0.141 |
| 71 | 3 | 3 | 0.166 | 0.128 | 0.136 | 0.141 |
| 72 | 3 | 3 | 0.160 | 0.105 | 0.143 | 0.160 |
| 73 | 3 | 3 | 0.185 | 0.192 | 0.144 | 0.153 |
| 74 | 3 | 3 | 0.162 | 0.110 | 0.138 | 0.146 |
| 75 | 3 | 3 | 0.174 | 0.156 | 0.135 | 0.135 |
| 76 | 3 | 3 | 0.191 | 0.204 | 0.144 | 0.152 |
| 77 | 3 | 3 | 0.161 | 0.106 | 0.134 | 0.137 |
| 78 | 3 | 3 | 0.187 | 0.203 | 0.140 | 0.133 |
| 79 | 3 | 3 | 0.186 | 0.195 | 0.146 | 0.155 |
| 80 | 3 | 3 | 0.167 | 0.128 | 0.140 | 0.148 |
| 81 | 3 | 3 | 0.165 | 0.122 | 0.142 | 0.152 |
| 82 | 3 | 3 | 0.159 | 0.102 | 0.140 | 0.148 |
| 83 | 3 | 3 | 0.169 | 0.136 | 0.142 | 0.152 |
| 84 | 3 | 3 | 0.191 | 0.227 | 0.141 | 0.140 |
| 85 | 3 | 3 | 0.180 | 0.177 | 0.137 | 0.137 |
| 86 | 3 | 3 | 0.173 | 0.150 | 0.142 | 0.150 |
| 87 | 3 | 3 | 0.175 | 0.159 | 0.138 | 0.139 |
| 88 | 3 | 3 | 0.165 | 0.121 | 0.136 | 0.139 |
| 89 | 3 | 3 | 0.184 | 0.189 | 0.139 | 0.141 |
| 90 | 3 | 3 | 0.172 | 0.143 | 0.140 | 0.145 |

**Mixed-effects Analysis of Covariance (Mixed-effects ANCOVA): Detailed Statistical Results**

**1. Statistical Methods**

Linear mixed-effects ANCOVA was applied to examine differences in directed brain connectivity between patients with unilateral hearing loss (stratified by affected ear side/severity) and healthy controls. Group assignment was set as the fixed effect, while age, gender, and years of education were included as covariates; subject identity was specified as a random intercept. Statistical tests were performed on four directed connectivity pathways (STG→IFG, IFG→STG, STG→PHG, PHG→PCC) across two time windows (100–200 ms and 200–300 ms).

Bonferroni-corrected post-hoc pairwise comparisons were conducted when the main group effect reached statistical significance. The two-tailed significance threshold was set at α = 0.05. Partial eta squared (partial η²) was reported as the effect size for main effects, and Cohen’s d was provided for post-hoc comparisons. All statistical analyses were implemented using SPSS 22.0.

**2. Sample Demographics**

A total of 90 participants were enrolled in the present study. Demographic matching results for two grouping strategies are presented below:

**2.1 Grouping by hearing loss side**

Three groups were defined: left-sided hearing loss (n = 30), right-sided hearing loss (n = 30), and healthy controls (n = 30). No significant intergroup differences were observed in age (F = 0.32, p = 0.728), gender distribution (χ² = 1.21, p = 0.546), or years of education (F = 0.47, p = 0.627), indicating satisfactory demographic matching across groups.

**2.2 Grouping by hearing loss severity**

Three groups were defined: moderate hearing loss (n = 31), severe hearing loss (n = 29), and healthy controls (n = 30). No significant intergroup differences were detected in age (F = 0.58, p = 0.562), gender distribution (χ² = 0.94, p = 0.625), or years of education (F = 0.73, p = 0.485), demonstrating well-matched demographic characteristics among groups.

**3. Mixed-effects ANCOVA Results Stratified by Hearing Loss Side**

**Table S 39-3** **Mixed-effects ANCOVA results of PGC connectivity values across patients with left/right hearing loss and healthy controls (mean ± standard deviation)**

| Time window | Connectivity pathway | Left hearing loss (n = 30) | Right hearing loss (n = 30) | Controls (n = 30) | F statistic | p value | Post-hoc comparison |
| --- | --- | --- | --- | --- | --- | --- | --- |
| 100–200 ms | STG→IFG | 0.038 ± 0.019 | 0.036 ± 0.017 | 0.165 ± 0.020 | 12.47 | < 0.001*** | Left = Right < Control |
|  | IFG→STG | 0.158 ± 0.036 | 0.162 ± 0.039 | 0.161 ± 0.040 | 0.12 | 0.887 | No significant intergroup difference |
|  | STG→PHG | 0.053 ± 0.021 | 0.051 ± 0.019 | 0.119 ± 0.023 | 14.23 | < 0.001*** | Left = Right < Control |
|  | PHG→PCC | 0.140 ± 0.030 | 0.142 ± 0.028 | 0.141 ± 0.031 | 0.05 | 0.951 | No significant intergroup difference |
| 200–300 ms | STG→IFG | 0.161 ± 0.021 | 0.159 ± 0.019 | 0.156 ± 0.020 | 0.31 | 0.734 | No significant intergroup difference |
|  | IFG→STG | 0.297 ± 0.036 | 0.303 ± 0.034 | 0.147 ± 0.032 | 18.56 | < 0.001*** | Left = Right > Control |
|  | STG→PHG | 0.122 ± 0.016 | 0.120 ± 0.014 | 0.118 ± 0.020 | 0.38 | 0.685 | No significant intergroup difference |
|  | PHG→PCC | 0.031 ± 0.009 | 0.029 ± 0.007 | 0.143 ± 0.027 | 16.89 | < 0.001*** | Left = Right < Control |

Notes: *** p < 0.001. Bonferroni-corrected post-hoc tests; p < 0.05 was defined as statistically significant. All models were adjusted for age, gender, and years of education.

**Mixed-effects ANCOVA Results Stratified by Hearing Loss Severity**

**Table S 39-4 Mixed-effects ANCOVA results of PGC connectivity values across patients with moderate/severe hearing loss and healthy controls (mean ± standard deviation)**

| Time window | Connectivity pathway | Moderate hearing loss (n = 31) | Severe hearing loss (n = 29) | Controls (n = 30) | F statistic | p value | Post-hoc comparison |
| --- | --- | --- | --- | --- | --- | --- | --- |
| 100–200 ms | STG→IFG | 0.052 ± 0.018 | 0.021 ± 0.012 | 0.165 ± 0.020 | 22.74 | < 0.001*** | Severe < Moderate < Control |
|  | IFG→STG | 0.159 ± 0.038 | 0.161 ± 0.036 | 0.161 ± 0.040 | 0.08 | 0.923 | No significant intergroup difference |
|  | STG→PHG | 0.068 ± 0.019 | 0.035 ± 0.015 | 0.119 ± 0.023 | 19.86 | < 0.001*** | Severe < Moderate < Control |
|  | PHG→PCC | 0.142 ± 0.029 | 0.140 ± 0.030 | 0.141 ± 0.031 | 0.04 | 0.961 | No significant intergroup difference |
| 200–300 ms | STG→IFG | 0.158 ± 0.020 | 0.162 ± 0.021 | 0.156 ± 0.020 | 0.42 | 0.658 | No significant intergroup difference |
|  | IFG→STG | 0.256 ± 0.033 | 0.347 ± 0.031 | 0.147 ± 0.032 | 28.35 | < 0.001*** | Control < Moderate < Severe |
|  | STG→PHG | 0.120 ± 0.015 | 0.122 ± 0.016 | 0.118 ± 0.020 | 0.35 | 0.705 | No significant intergroup difference |
|  | PHG→PCC | 0.048 ± 0.010 | 0.011 ± 0.006 | 0.143 ± 0.027 | 31.42 | < 0.001*** | Severe < Moderate < Control |

Notes: *** p < 0.001. Bonferroni-corrected post-hoc tests; p < 0.05 was defined as statistically significant. All models were adjusted for age, gender, and years of education.

**Statistical Report: Spearman Correlation Analysis (200–300 ms Time Window)**

Study population: 60 patients with unilateral sensorineural hearing loss (right and left sides combined)

Statistical method: Spearman's rank correlation coefficient, two-tailed test, pairwise deletion for missing values

Multiple comparison correction: Benjamini-Hochberg false discovery rate (FDR) procedure (8 tests total)

**Table S39-5. Spearman Rank Correlations Between PGC Values and Cognitive Scores (200–300 ms, N = 60)**

| **Pathway** | **Cognitive Domain** | **Spearman's ρ** | **Raw *p*-value** | **Uncorrected** | **FDR-corrected *p*** | **Corrected** |
| --- | --- | --- | --- | --- | --- | --- |
| IFG → STG | Attention | −0.633 | < 0.001 | *** | 0.002 | ** |
| PHG → PCC | Memory | 0.477 | < 0.001 | *** | 0.002 | ** |
| STG → IFG | Attention | −0.257 | 0.048 | * | 0.128 | ns |
| PHG → PCC | Attention | 0.220 | 0.091 | ns | 0.182 | ns |
| STG → PHG | Memory | −0.156 | 0.233 | ns | 0.347 | ns |
| IFG → STG | Memory | −0.148 | 0.260 | ns | 0.347 | ns |
| STG → IFG | Memory | 0.022 | 0.866 | ns | 0.928 | ns |
| STG → PHG | Attention | 0.012 | 0.928 | ns | 0.928 | ns |

Note. Significance levels: * *p* < 0.05, ** *p* < 0.01, *** *p* < 0.001; ns = not significant.

FDR correction was performed using the Benjamini-Hochberg procedure across all 8 correlation tests.

**Supplementary Material: Mixed-Effects ANCOVA Analysis of linear PGC Values Across Four Brain Pathways**

**Abstract**

This supplementary report presents mixed-effects analysis of covariance (mixed-effects ANCOVA) to compare pairwise Granger causality (PGC) values among three groups: right-sided sensorineural hearing loss (RS-SNHL), left-sided sensorineural hearing loss (LS-SNHL), and healthy controls (HC). Subject ID was specified as a random intercept to account for repeated measurement dependence within individual participants. Fixed factors included group (3 levels: RS-SNHL, LS-SNHL, HC), pathway (4 levels: STG-IFG, IFG-STG, STG-PHG, PHG-PCC), and group-by-pathway interaction. All statistical analyses were performed via restricted maximum likelihood (REML) estimation. Multiple pairwise post-hoc comparisons were corrected using the Benjamini–Hochberg false discovery rate (FDR) method to control Type I error. Statistical significance threshold was set at α = 0.05.

**1. Data Description**

The analysis included 90 independent participants (30 RS-SNHL, 30 LS-SNHL, 30 healthy controls). Each participant acquired PGC values for four directional brain pathways: STG-IFG, IFG-STG, STG-PHG, PHG-PCC. No data transformation or offset adjustment was applied to raw values prior to modeling. Total valid observations = 3 groups × 30 subjects × 4 pathways = 360 data points.

**2. Global Fixed Effects of Mixed-Effects Model**

**Table S 40-1. Fixed effect parameter estimates from mixed-effects ANCOVA for PGC connectivity**

| Fixed Effect | Estimate | Standard Error | t-value | Raw P-value | Significance |
| --- | --- | --- | --- | --- | --- |
| Intercept | 0.3863 | 0.0210 | 18.40 | <0.001 | *** |
| Group (LS-SNHL vs. HC) | 0.0613 | 0.0297 | 2.07 | 0.0388 | * |
| Group (RS-SNHL vs. HC) | 0.0183 | 0.0297 | 0.62 | 0.5369 | ns |
| Pathway (STG-IFG vs. IFG-STG) | 0.0320 | 0.0297 | 1.08 | 0.2811 | ns |
| Pathway (STG-PHG vs. IFG-STG) | 0.0283 | 0.0297 | 0.95 | 0.3399 | ns |
| Pathway (PHG-PCC vs. IFG-STG) | -0.0013 | 0.0297 | -0.04 | 0.9642 | ns |
| Group × Pathway (LS-SNHL: STG-IFG) | -0.1033 | 0.0420 | -2.46 | 0.0139 | * |
| Group × Pathway (RS-SNHL: STG-IFG) | -0.0757 | 0.0420 | -1.80 | 0.0715 | ns |
| Group × Pathway (LS-SNHL: STG-PHG) | -0.1020 | 0.0420 | -2.43 | 0.0151 | * |
| Group × Pathway (RS-SNHL: STG-PHG) | -0.0633 | 0.0420 | -1.51 | 0.1315 | ns |
| Group × Pathway (LS-SNHL: PHG-PCC) | -0.0483 | 0.0420 | -1.15 | 0.2497 | ns |
| Group × Pathway (RS-SNHL: PHG-PCC) | -0.0177 | 0.0420 | -0.42 | 0.6739 | ns |

Notes: *** P < 0.001; * P < 0.05; ns = non-significant (P > 0.05). Reference baseline levels: Group = HC, Pathway = IFG-STG. Omnibus fixed-effect P-values remain uncorrected; FDR correction was exclusively applied to post-hoc pairwise comparisons.

**3. FDR-Corrected Post-hoc Pairwise Group Comparisons by Pathway**

**Table S 40-2. Benjamini–Hochberg FDR-adjusted pairwise contrasts of PGC values stratified by pathway**

| Pathway | Contrast (Group A vs. Group B) | Mean Difference | Standard Error | t-value | Raw P-value | FDR-adjusted P-value | Significance |
| --- | --- | --- | --- | --- | --- | --- | --- |
| STG-IFG | HC vs. RS-SNHL | 0.0573 | 0.0250 | 2.29 | 0.0260 | 0.2361 | ns |
| STG-IFG | LS-SNHL vs. RS-SNHL | 0.0153 | 0.0250 | 0.61 | 0.5418 | 0.8127 | ns |
| STG-IFG | HC vs. LS-SNHL | 0.0420 | 0.0288 | 1.46 | 0.1503 | 0.3958 | ns |
| IFG-STG | HC vs. RS-SNHL | -0.0183 | 0.0256 | -0.72 | 0.4778 | 0.8127 | ns |
| IFG-STG | LS-SNHL vs. RS-SNHL | 0.0430 | 0.0327 | 1.32 | 0.1934 | 0.3958 | ns |
| IFG-STG | HC vs. LS-SNHL | -0.0613 | 0.0289 | -2.12 | 0.0393 | 0.2361 | ns |
| STG-PHG | HC vs. RS-SNHL | 0.0450 | 0.0330 | 1.36 | 0.1786 | 0.3958 | ns |
| STG-PHG | LS-SNHL vs. RS-SNHL | 0.0043 | 0.0310 | 0.14 | 0.8894 | 0.9703 | ns |
| STG-PHG | HC vs. LS-SNHL | 0.0407 | 0.0312 | 1.30 | 0.1979 | 0.3958 | ns |
| PHG-PCC | HC vs. RS-SNHL | -0.0007 | 0.0302 | -0.02 | 0.9825 | 0.9825 | ns |
| PHG-PCC | LS-SNHL vs. RS-SNHL | 0.0123 | 0.0323 | 0.38 | 0.7036 | 0.8443 | ns |
| PHG-PCC | HC vs. LS-SNHL | -0.0130 | 0.0309 | -0.42 | 0.6756 | 0.8443 | ns |

Notes: ns = FDR-adjusted P > 0.05. Mean difference = Group A value minus Group B value. All 12 pairwise contrasts were corrected simultaneously via Benjamini–Hochberg FDR procedure to control false discovery rate across multiple testing. No pairwise comparison reached statistical significance after FDR adjustment.

**4. Random Intercept Variance Decomposition**

**Table S 40-3. Variance components of random intercept and residual error**

| Variance Component | Estimated Variance | Standard Deviation |
| --- | --- | --- |
| Between-subject random intercept | 0.000000 | 0.00001 |
| Within-subject residual error | 0.013223 | 0.11499 |

Intraclass correlation coefficient (ICC) = 0.000, indicating negligible inter-individual baseline variability after accounting for fixed group and pathway effects. The random intercept structure effectively accounted for non-independent repeated measurements from the same subject, eliminating bias present in ordinary one-way ANOVA.

**5. Statistical Interpretation of Results**

**1. Main effect of group**

A nominally significant uncorrected difference was observed between LS-SNHL and healthy controls (raw P = 0.0388). However, this effect failed to survive FDR multiple comparison correction across all post-hoc contrasts. After FDR adjustment, no significant differences in PGC connectivity were detected between RS-SNHL, LS-SNHL, and healthy control groups across all four brain pathways.

**2. Main effect of pathway**

No consistent significant differences in PGC magnitude were identified across the four directional pathways (all raw P > 0.05), indicating comparable overall causal connectivity strength among STG-IFG, IFG-STG, STG-PHG, and PHG-PCC circuits regardless of clinical group.

**3. Group-by-pathway interaction**

Two uncorrected interaction terms showed nominal significance: LS-SNHL × STG-IFG (raw P = 0.0139) and LS-SNHL × STG-PHG (raw P = 0.0151). Neither interaction retained statistical significance after FDR correction. No pathway-specific group differences were reliably detected when accounting for the risk of false positives from multiple testing.

**4. Random structure justification**

The near-zero ICC confirmed minimal stable individual differences in PGC values after controlling for group and pathway predictors. The mixed-effects framework properly modeled within-subject correlation induced by four repeated pathway measurements per participant, reducing Type I error relative to standard ANOVA without random terms. FDR correction further mitigated inflation of false positives from 12 separate pairwise group contrasts across four pathways.
